# Loss of the *USP22* deubiquitylase confers resistance to chemotherapy in small cell lung cancer

**DOI:** 10.1038/s41467-026-75117-2

**Published:** 2026-07-08

**Authors:** Scott Best, Daniel S. Hippe, Eli Grunblatt, Jackson Fatherree, Pritha Chanana, Feinan Wu, Richard Ivey, Jacob J. Kennedy, David Sokolov, Ali Ibrahim, Haodong Xu, Raymond J. Monnat, Lucas B. Sullivan, Patrick Paddison, Amanda G. Paulovich, David MacPherson

**Affiliations:** 1 Human Biology Division, Fred Hutchinson Cancer Center, Seattle, WA USA; 2 Molecular and Cellular Biology Program, University of Washington, Seattle, WA USA; 3 Clinical Research Division, Fred Hutchinson Cancer Center, Seattle, WA USA; 4 Genomics and Bioinformatics Shared Resource, Fred Hutchinson Cancer Center, Seattle, WA USA; 5 Translational Science and Therapeutics Division, Fred Hutchinson Cancer Center, Seattle, WA USA; 6 Department of Pathology, Wake Forest University School of Medicine, Winston-Salem, USA; 7 Departments of Lab Medicine and Pathology, University of Washington, Seattle, WA USA; 8 Department of Biochemistry, University of Washington, Seattle, WA USA; 9 Basic Sciences Division, Fred Hutchinson Cancer Center, Seattle, WA USA; 10 Department of Genome Sciences, University of Washington, Seattle, WA USA; 11 Public Health Sciences Division, Fred Hutchinson Cancer Center, Seattle, WA USA

**Keywords:** Small-cell lung cancer, Cancer genetics, Cancer therapeutic resistance

## Abstract

Small cell lung cancer (SCLC) responds exceptionally well to cytotoxic chemotherapy. However, relapse with the emergence of chemoresistant disease is rapid and accompanied by poor treatment outcomes. To understand the genetic basis of chemoresistance in SCLC, we apply in vivo CRISPR deletion screening to patient-derived xenograft (PDX) models. Top screen hits include genes encoding components of the transcriptional co-activator SAGA (Spt-Ada-Gcn5 acetyltransferase) complex. We demonstrate that deletion of the SAGA deubiquitylase *USP22* confers cisplatin-etoposide resistance in two chemosensitive PDX models, and that restoring expression in a PDX model harboring homozygous truncating mutation of *USP22* re-sensitizes tumors to chemotherapy. USP22 loss increases gene body histone H2AK119 monoubiquitylation at key regulators of neuronal differentiation and suppresses neural and neuroendocrine gene expression including targets of ASCL1. Chemoresistance following USP22 loss reflects attenuated DNA damage-driven phosphorylation events and apoptosis, in conjunction with increased expression of glycolysis and hypoxia-related genes. Glycolysis program upregulation may reflect a targetable vulnerability, as inhibition of GLUT1 re-sensitizes USP22-null tumors to chemotherapy.

## Abstract (web summary)

While small cell lung cancer (SCLC) typically responds well to chemotherapy, resistance often develops rapidly with limited therapeutic options. Here, using an in vivo CRISPR deletion screen in patient derived models of SCLC, the authors identify the SAGA deubiquitylase USP22 as a driver of cisplatin resistance in SCLC.

## Introduction

Small cell lung cancer (SCLC) is a highly lethal, neuroendocrine lung carcinoma characterized by early metastatic spread with initial sensitivity to chemotherapy that is typically followed within months by relapse with chemoresistant disease^1,2^. This rapid transition to chemoresistant disease leads to poor treatment outcomes. The addition of anti-PDL1 antibodies to platinum/etoposide (cis-eto) chemotherapy in 2019 led to durable responses in only a small subset of patients^3,4^. A better understanding of the origins of treatment failure and how to improve outcomes has been hampered by few genomic analyses of SCLC following treatment, and few good preclinical models in which to understand the factors that drive relapse and chemoresistance.

This unmet need for genomic information from relapsed patients is just beginning to be addressed^5,6^. For example, a study of 30 chemo-refractory SCLC patient samples found that recurrent mutations in regulators of WNT signaling were associated with chemotherapy relapse^6^. SCLC PDX models have been shown to faithfully capture and perpetuate SCLC drug sensitivity and resistance phenotypes observed in patients^7–9^, in contrast to SCLC cell lines derived from chemo-naïve vs relapsed patients^10^. SCLC PDX models have also been used to derive cisplatin-etoposide-resistant derivatives, where the analysis of these chemoresistant PDX variants identified EZH2-mediated epigenetic silencing of *SLFN11*^7^ as critical for sensitivity to DNA damaging agents^11,12^. Our group has shown that overexpression of *MYCN* and *MYCL* could switch highly chemosensitive PDX models of SCLC to therapy resistance^13^, while an independent analysis of SCLC PDX models and patient samples found that extra-chromosomal *MYC* family ecDNA amplification arises with the initiation of chemotherapy treatment and contributes to resistance^14^. These observations support the use of PDX models as clinically relevant models to investigate mechanisms of SCLC chemoresistance.

Intratumoral heterogeneity and plasticity may contribute to SCLC chemoresistance, as evidenced by increased heterogeneity and enrichment of unique gene expression patterns post-relapse from longitudinal single-cell profiling of SCLC circulating tumor cells taken before and after chemotherapy^9^. SCLC exhibits heterogeneity based on expression of lineage-regulating transcription factors ASCL1, NEUROD1 and POU2F3^15–19^ and a subset of SCLC has been associated with an immune/inflamed tumor phenotype with reduced sensitivity to topotecan chemotherapy^20,21^^,^. Heterogeneity of expression of neuroendocrine and *MYC* family genes has also been documented^15–17,22–24^. Genetically engineered mouse models of SCLC have further revealed the co-existence of neuroendocrine cells and chemoresistant non-neuroendocrine NOTCH-expressing cells^22^. These observations provide clues to the origins of chemoresistance in SCLC and emphasize the importance of better understanding the mechanisms responsible in order to improve treatment outcomes for SCLC patients.

In order to address this challenge, we recently developed a strategy to identify genetic changes that confer SCLC chemoresistance by in vivo functional genetic screening of chemosensitive SCLC PDX models^25^. An important initial hit was *KEAP1*, a master negative regulator of the NRF2 oxidative stress response pathway^26,27^. We further demonstrated that knockout of *KEAP1* conferred chemoresistance in a chemosensitive PDX model^25^.

In this work, we demonstrate that SAGA complex members identified in CRISPR deletion screens drive SCLC chemoresistance and interrogate mechanisms by which loss of the SAGA complex deubiquitylase USP22 alters SCLC chemotherapeutic response.

## Results

### CRISPR deletion screens reveal SAGA complex members as candidate drivers of SCLC chemoresistance

**Fig. 1:**
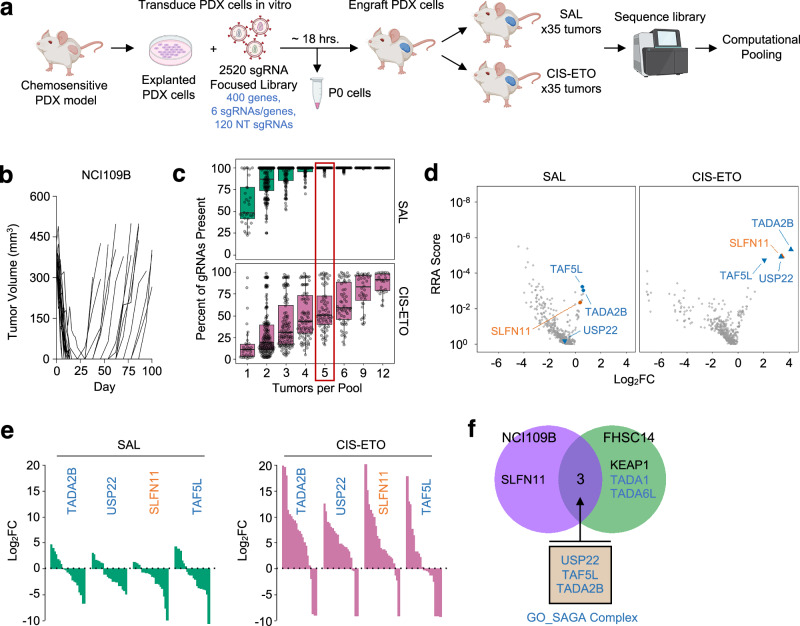
In vivo CRISPR-Cas9 deletion screens identify drivers of cisplatin-etoposide resistance in SCLC PDX models. **a** Schematic of the in vivo CRISPR-Cas9 deletion screen conducted using a custom focused 2520 sgRNA focused library to identify loss-of-function drivers of cis-eto resistance in SCLC PDX models. Created in BioRender. Best, S. (2026) https://BioRender.com/22p9q6j. **b** NCI109B PDX models were screened with the focused gRNA library and treated with 2 weekly cycles of saline or cis-eto. The tumor volume kinetics for the largest of two tumors implanted into each mouse is plotted to show tumor regression and relapse in response to cis-eto treatment. **c** NCI109B tumors screened with the focused gRNA library underwent computational pooling^25^. The percentage of focused library sgRNAs present per pool is shown. Five tumors per pool (*n* = 7 pools per treatment condition) was selected for subsequent analyses based on the high sgRNA coverage achieved in the saline group. Boxes indicate 25th percentile (lower bound), median (center line), and 75th percentile (upper bound) of data. Whiskers extend to minima or maxima data point values or up to 1.5 times the interquartile range (IQR) from box bounds. **d** Volcano plots of MAGeCK analyses results from NCI109B tumors screened with the focused sgRNA library. Genes with FDR < 0.05 or FDR 0.05-0.20 are displayed with right-side up triangles or upside-down triangles, respectively. Genes labeled in blue are SAGA complex members enriched in the CIS-ETO vs P0 condition with at least an FDR < 0.20. **e** Waterfall plots showing treatment vs P0 Log_2_FC of the top 3 sgRNAs per gene targeting *TADA2B*, *USP22*, *SLFN11*, and *TAF5L* for all NCI109B tumor pools. Each vertical bar is one sgRNA from one pooled tumor. **f** Overlap of genes with at least 3 of their 6 gRNAs enriched (Log_2_FC > 2, FDR < 0.05) from the NCI109B and FHSC14^25^ focused CRISPR-Cas9 deletion screens. Source Data is provided in the Source Data File.

We recently described a custom CRISPR-KO sgRNA library targeting 400 genes with 6 gRNAs/gene and 120 non-targeting control gRNAs to use to identify loss-of-function drivers of SCLC PDX cisplatin-etoposide (cis-eto) resistance^25^. The sgRNA library included altered genes identified by SCLC genomic studies^15,17^ or implicated in SCLC chemoresistance such as *SLFN11* and WNT pathway members^6,7^. We also included genes that displayed sgRNA enrichment after cis-eto chemotherapy selection in a pilot genome-scale CRISPR deletion screen that used the Human CRISPR Knockout Pooled Library (GeCKO v2)^28^ in the FHSC14 SCLC PDX model. Pilot screen data (Supplementary Fig. 1a, b, Supplementary Data 1) indicated that a pooled in vivo PDX whole genome screen with control and cis-eto-treated arms was feasible, but not well enough powered to identify statistically significant hits despite identifying multiple members of the SAGA complex (Supplementary Fig. 1). We thus included sgRNAs targeting this complex and 123 additional top-scoring candidate genes in a more focused screen library (Fig. 1a, Supplementary Data 2) to identify loss-of-function drivers of cis-eto chemoresistance in the SCLC PDX models (Fig. 1a).

We employed an SCLC PDX model derived from the NCI Patient Derived Models Repository, model 594431-109-B, abbreviated as NCI109B, which exhibits exquisite sensitivity to chemotherapy as two weekly cycles of chemotherapy typically leads to complete tumor regression (Fig. 1b). Screens were performed by dissociating NCI109B tumors into single-cell suspensions for transduction with concentrated lentivirus containing the focused CRISPR-KO sgRNA library. After 18 h ex vivo, transduced cells were divided for subcutaneous re-implantation into the flanks of NSG mice (1 × 10^6^ cells per injection) or held in culture for 72 h as a transduced P_0_ reference population. Once tumors reached 300 mm^3^, mice were randomly assigned to a cis-eto or saline control treatment group. Chemotherapy treatment consisted of two weekly cycles of cis-eto that resulted in complete tumor regression. Tumors that recurred off treatment were collected after re-growth to pre-treatment volume (Fig. 1b). A total of 35 saline-treated tumors and 35 cis-eto tumors were sequenced, where individual tumors in the saline group contained ~50% of all gRNAs present (Fig. 1c). In order to identify consistently enriched sgRNAs, we computationally pooled read counts from 7 pooled batches of randomly assigned tumors (5 independent tumors/pool/treatment group) such that the pooled libraries included > 95% of the saline tumor input library (Fig. 1c). These pooled library data were then analyzed using MAGeCK^29^. In cis-eto-treated tumors, *SLFN11*, *USP22*, *TAF5L*, *TADA2B* had at least 3 of 6 of their corresponding sgRNAs enriched (Log_2_FC > 2, FDR < 0.05) relative to P_0_ tumors, with *SLFN11*, *USP22*, and *TADA2B* having a gene-level positive enrichment FDR < 0.05 and *TAF5L* FDR < 0.1 (Fig. 1d, e, Supplementary Data 3-4). In saline-treated control tumors these genes had $\le \,$1 corresponding sgRNAs enriched. Prior reports had demonstrated that epigenetic suppression of *SLFN11* conferred chemoresistance in SCLC PDX models^7^, a finding that supports the ability of our screening strategy to identify known drivers of SCLC chemoresistance. The other top screen hits, *USP22*, *TADA2B*, and *TAF5L*, encode members of the SAGA complex, a transcriptional co-activator that regulates gene expression through histone acetyltransferase and deubiquitylase activities^30–32^. Of note, *USP22* and *TADA1* were the top two hits in our pilot genome-wide study (Supplementary Fig. 1a), though neither had previously been associated with chemoresistance in any cancer type.

The recognition of SCLC biological heterogeneity has led to proposed SCLC subtypes based on the activation of key transcription factors such as ASCL1 (SCLC-A) and NEUROD1 (SCLC-N). NCI109B, the PDX model used in this study, expresses high levels of NEUROD1^33^. In order to replicate and extend our functional screening results, we compared NCI109B results with our previous screen performed in the SCLC PDX model FHSC14^25^ that has distinct biological features including high levels of ASCL1^34^. Among the top screen hits in FHSC14 was *KEAP1*, a master regulator of the NRF2 anti-oxidative stress response pathway^26,27^ for which we demonstrated that loss-of-function could drive chemoresistance^25^. FHSC14 cis-eto-treated tumors were also enriched in sgRNAs targeting SAGA complex members *USP22*, *TAF5L*, *TADA2B*, *TAF6L*, and *TADA1*. Both FHSC14 and NCI109B cis-eto-treated tumors were enriched for sgRNAs that targeted *USP22*, *TAF5L*, and *TADA2B* (Fig. 1f), where NCI109B was lineage transcription factor NEUROD1-active and FHSC14 ASCL1-high^25^. These results suggested the suppression of SAGA components might broadly promote chemoresistance in SCLC across different transcription factor-defined subtypes.

### *USP22* deletion confers SCLC PDX model chemoresistance

**Fig. 2:**
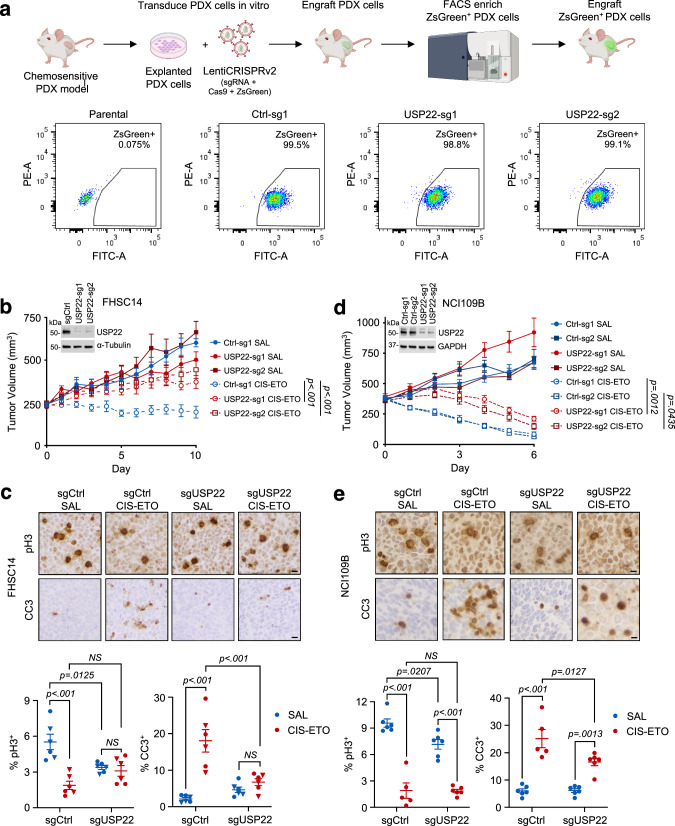
Generation of isogenic PDX models reveal *USP22* deletion as a driver of chemoresistance in SCLC. **a** Schematic of isogenic PDX model generation with lentiviral CRISPR-Cas9 mediated deletion of *USP22*. FACS plots show purity of transduced FHSC14 cells as assessed by ZsGreen expression relative to parental cells. Created in BioRender. Best, S. (2026) https://BioRender.com/22p9q6j. **b** Tumor volume over time growth curves of isogenic FHSC14 sgCtrl, USP22-sg1, and USP22-sg2 models treated with saline or cis-eto over a 10-day period. Data are means ± SEM; *n* = 5 animals per group, *n* = 4 animals per group for USP22-sg1 CIS-ETO and USP22-sg2 CIS-ETO. Top-left western blot shows USP22 protein loss in both USP22-sg1 and USP22-sg2 tumors relative to sgCtrl. **c** IHC analyses and representative images for pH3 and CC3 in FHSC14 Ctrl-sg1/2 and USP22-sg1/2 tumors treated for 48 h with saline or cis-eto. Data are means ± SEM; *n* = 6 tumors per group. Triangles depict data from Ctrl-sg1 and USP22-sg1 tumors while circles depict Ctrl-sg2 and USP22-sg2 tumors. Scale bar, 10 µm. **d** Tumor volume growth curves of isogenic NCI109B Ctrl-sg1, Ctrl-sg2, USP22-sg1, and USP22-sg2 models treated with saline or cis-eto over a 6-day period. Data are means ± SEM; *n* = 7 animals per group, *n* = 6 animals for Ctrl-sg2 CIS-ETO, *n* = 5 animals for Ctrl-sg2 SAL. Top-left western blot shows USP22 protein loss in USP22-sg1/2 relative to Ctrl-sg1/2 tumors. **e** IHC analyses and representative images for pH3 and CC3 in NCI09B sgCtrl and sgUSP22 tumors treated for 48 h with saline or cis-eto. Data are means ± SEM; *n* = 6 tumors per group, *n* = 5 tumors for sgCtrl CIS-ETO. Scale bar, 10 µm. Statistical analyses in (**b**,** d**), were performed by 2-way ANOVA with Tukey’s multiple comparisons test. Statistical analyses in (**c**,** e**), were performed by 1-way ANOVA with Tukey’s multiple comparisons test. Adjusted ANOVA *p* values are shown on figures; NS non-significant. Source Data is provided in the Source Data File.

In order to further investigate the role of SAGA in regulating the response to chemotherapy, we focused first on *USP22*, the top hit from our functional screen. This gene encodes a deubiquitinating subunit of SAGA that catalyzes removal of mono-ubiquitin from histone H2AK119ub that is associated with Polycomb-mediated gene silencing^35–37^. USP22 can also deubiquitinate histone H2BK120ub to promote transcriptional elongation^31,38–40^, as well as deubiquitinate several other non-histone targets^41–43^. We generated isogenic FHSC14 PDX models with CRISPR-Cas9 mediated deletion of *USP22* (Fig. 2a) by explanting and dissociating tumors prior to ex vivo lentivirus transduction to express either of two USP22 gRNAs (USP22-sg1/2) or one of two non-targeting gRNAs (Ctrl-sg1/2). The sgRNAs for these experiments were cloned into a lentiCRISPRv2 vector^44^ containing a ZsGreen selection marker (see Supplementary Data 5 for sgRNA sequences used). Eighteen hours after transduction, cells were re-implanted in the flanks of NSG mice for tumor regrowth after which we used fluorescence-activated cell sorting (FACS) to purify ZsGreen-positive cells. These were re-implanted and underwent subsequent rounds of sorting to form flank tumors that were > 98% ZsGreen positive (Fig. 2a, Supplementary Fig. 2a), with *USP22* deletion confirmed by western blotting (Fig. 2b).

To determine how *USP22* deletion influenced chemosensitivity, we generated flank tumors by expanding FHSC14 USP22-sg1/2 and sgCtrl cells in NSG mice, then treated mice with tumors that had reached ~250 mm^3^ with a 10-day regimen of cis-eto or saline together with daily measurements of tumor volume. Both USP22-sg1 and USP22-sg2 –expressing tumors were treatment resistant and continued to grow at similar rates, in contrast to sgCtrl expressing tumors that stopped growing or regressed in response to cis-eto treatment (Fig. 2b, Supplementary Fig. 2b). In separate experiments designed to assess the effect of *USP22* deletion on the short-term response to cis-eto, we collected FHSC14 Ctrl-sg1/2 and USP22-sg1/2-expressing tumors 48 h after treatment. In FHSC14 Ctrl-sg1/2 tumors, cis-eto treatment significantly reduced the proportion of mitotic cells while concurrently strongly induced apoptosis, as assessed respectively by phospho-histone H3 S10 (pH3) and cleaved-caspase-3 (CC3) staining (Fig. 2c). In contrast, the proportion of pH3 and CC3-positive cells at 48 h did not change in USP22-sg1/2 tumors treated with cis-eto.

To further validate and extend these observations, we deleted *USP22* in PDX model NCI109B (Supplementary Fig. 2c) where we had identified sgRNAs targeting *USP22* were enriched in functional screens (Fig. 1d–f). Ctrl-sg1/2-expressing NCI109B tumors again decreased in volume more rapidly relative to USP22-sg1/2 tumors after cis-eto treatment (Fig. 2d, Supplementary Fig. 2d) with reduced cell proliferation and increased apoptosis at 48 hrs in both sgCtrl and sgUSP22-expressing tumors (Fig. 2e). Again, larger reductions were observed in sgCtrl versus sgUSP22. These results indicate that *USP22* deletion can promote chemoresistance in two independent SCLC PDX models, with a stronger effect in FHSC14.

### USP22 re-expression sensitizes a USP22-null SCLC PDX model to chemotherapy

**Fig. 3:**
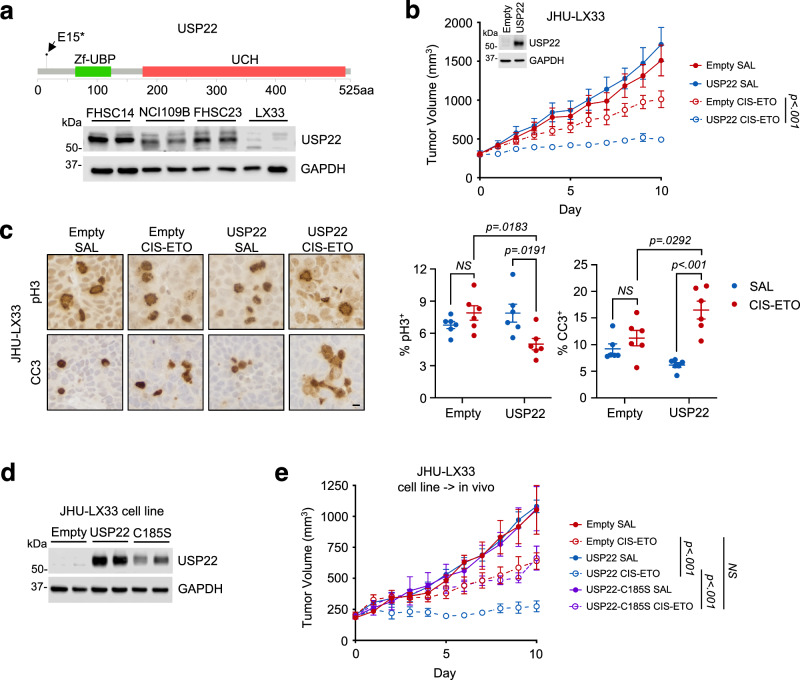
Restoration of USP22 resensitizes a USP22-null PDX model to chemotherapy. **a** Schematic of human USP22 protein showing an E15* truncating mutation present in the JHU-LX33 PDX model. Western blot shows USP22 protein in JHU-LX33 significantly depleted relative to three other SCLC PDX models. **b** Tumor volume growth curves of isogenic JHU-LX33 Empty-PLVX and USP22-PLVX models treated with saline or cis-eto over a 10-day period. Data are means ± SEM; *n* = 8 animals per group. Top-left western blot shows USP22 protein add-back in USP22-PLVX relative to Empty-PLVX tumors. Each lane is an independent tumor (*n* = 2 tumors per group), with each blot representative from two independent experiments. **c** IHC analysis and representative images for pH3 and CC3 in JHU-LX33 Empty-PLVX and USP22-PLVX tumors treated with saline or cis-eto over a course of 10 days. Data are means ± SEM; *n* = 6 tumors per group. Scale bar, 10 µm. **d** Western blot showing USP22 expression in JHU-LX33 Empty-PLVX, USP22-PLVX, and catalytic dead USP22-C815S-PLVX tumors. Each lane is an independent tumor (*n* = 2 tumors per group), with each blot representative from two independent experiments. **e** Isogenic JHU-LX33 Empty-PLVX, USP22-PLVX, and USP22-C815S-PLVX cell lines were injected into the flanks of NSG mice. Tumor volume growth curves of isogenic models treated with saline or cis-eto over a 10-day period. Data are means ± SEM; *n* = 5 animals per group. Statistical analyses in (**b**, **e**) were performed by 2-way ANOVA with Sidak’s (**b**) multiple comparisons test or Tukey’s (**e**) multiple comparisons test. Statistical analyses in **c** were performed by 1-way ANOVA with Tukey’s multiple comparisons test. Adjusted ANOVA *p* values are shown on figures; NS non-significant. Source Data is provided in the Source Data File.

*USP22* has not previously been described as a target of inactivating mutations in SCLC; however, examination of genomic whole exome data from a bank of PDX models revealed that SCLC PDX model JHU-LX33 harbors a homozygous E15* nonsense mutation in *USP22* (Fig. 3a, see Supplementary Data 6 showing this and other reported USP22 mutations in SCLC). Western blot analyses using tissue from this model confirmed loss of USP22 protein (Fig. 3a). In contrast to the highly chemosensitive models used for our screens, JHU-LX33 is only modestly sensitive to chemotherapy (Fig. 3b). In order to test whether *USP22* inactivation in this PDX model contributes to reduced chemotherapy response, we engineered isogenic JHU-LX33 derivatives that re-expressed either a *USP22* cDNA (USP22-PLVX) or an empty vector (Empty-PLVX) expressing a ZsGreen selection marker (Supplementary Fig. 3a). Empty vector JHU-LX33 tumors lacking USP22 were markedly chemoresistant, with robust growth despite a 10 day course of cis-eto treatment (Fig. 3b, Supplementary Fig. 3b), whereas tumors re-expressing USP22 displayed slower growth together with reduced pH3 and increased CC3 staining in response to cis-eto treatment (Fig. 3b, c). In contrast, the same treatment regimen did not cause significant changes in pH3 or CC3 staining in tumors derived from empty vector JHU-LX33 cells.

### USP22 deubiquitylase function is required for chemotherapeutic response

USP22 functions as a deubiquitylase in the SAGA complex. In order to determine whether this catalytic activity is required for SCLC chemotherapeutic response, we extended the engineered JHU-LX33 PDX model to compare the cis-eto sensitivity of tumors that expressed a USP22 catalytic dead mutant, USP22-C185S^45,46^ cDNA, wild-type USP22 cDNA, or the empty vector PLVX-ZsGreen selection marker (Fig. 3d). Isogenic JHU-LX33 tumors in NSG mice that re-expressed USP22 grew significantly slower when treated with cis-eto that control empty vector tumors (Fig. 3e, Supplementary Fig. 3c). In contrast, tumors expressing catalytically dead USP22-C185S were resistant to cis-eto and grew at rates comparable to empty vector control tumors. These results indicate that USP22 is an important determinant of SCLC chemosensitivity, and that loss of USP22 or its deubiquitylase function promotes chemoresistance in PDX models. These results further support the findings from our initial loss-of-function genetic screens.

### Global proteomic analyses reveal USP22 roles in SCLC

**Fig. 4:**
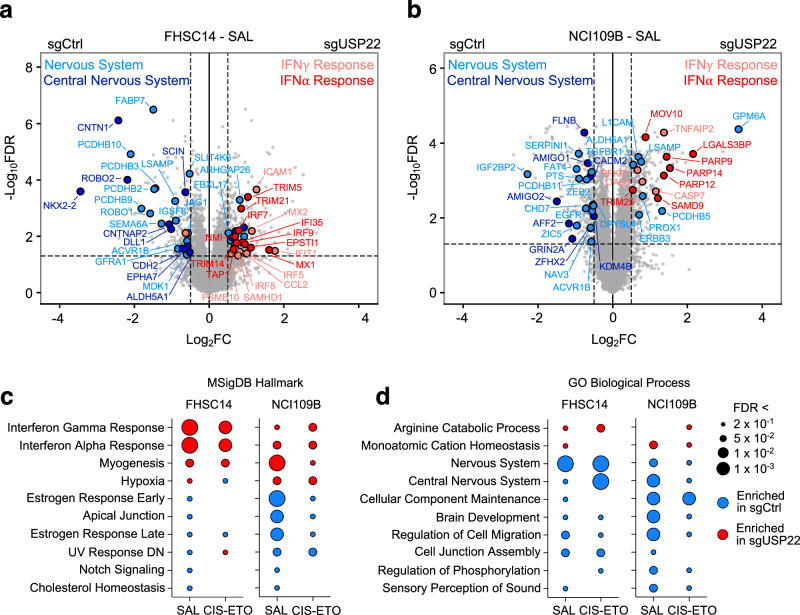
Global proteomic analyses of SCLC PDX models with *USP22* deletion. Volcano plots of global proteomics data from saline-treated FHSC14 USP22-sg1/2 vs Ctrl-sg1/2 tumors (*n* = 2 tumors per sgRNA for a total of *n* = 4 tumors per group) (**a**) and NCI109B sgUSP22 vs sgCtrl tumors (*n* = 3 tumors per group) (**b**). Differentially expressed upregulated (Log_2_FC > 0.5, FDR < 0.05) or downregulated (Log_2_FC < −0.5, FDR < 0.05) proteins belonging to the indicated Gene Ontology Biological Process 2023 and MSigDB Hallmark 2020 pathways are labeled in blue and red, respectively. **c**, **d** Upregulated or downregulated proteins in FHSC14 and NCI109B sgUSP22 vs sgCtrl tumors treated for 48 h with saline or cis-eto were independently queried for pathway enrichment using Enrichr. The top commonly enriched or depleted pathways from MSigDB Hallmark (**c**) and Gene Ontology Biological Process (**d**) gene set databases from both PDX models with *USP22* deletion are shown. Blue circles indicate pathways enriched in sgCtrl and red circles indicate pathways enriched in sgUSP22 tumors. Point size scaled to FDR.

In order to gain additional mechanistic insight into USP22 expression and activity as important determinants of SCLC chemosensitivity, we performed global proteomic analyses. These compared isogenic FHSC14 USP22-sg1/2 vs sgCtrl-sg1/2 and NCI109B sgUSP22 vs sgCtrl tumors that had been treated for 48 h with saline or with cis-eto (Fig. 4a, b, Supplementary Fig. 4a, b). Upregulated (Log_2_FC > 0.5, FDR < 0.05) or downregulated (Log_2_FC < −0.5, FDR < 0.05) proteins in sgUSP22 tumors from each PDX model (Supplementary Data 7-8) were queried for MSigDB Hallmark and GO Biological Process pathway enrichment using Enrichr^47,48^ (Fig. 4c, d, Supplementary Fig. 4c–f). Top pathways commonly enriched in sgUSP22 relative to sgCtrl tumors in both PDX models included Interferon Alpha Response, Interferon Gamma Response, Myogenesis, and Hypoxia (Fig. 4c, d). In *USP22-*deleted FHSC14 tumors, protein sets including interferon response pathways, inflammatory response, regulation of T-cell activation, allograft rejection, and IL2/STAT5 signaling were among the top 10 most significantly enriched Hallmark pathways (Supplementary Fig. 4c). These findings are consistent with multiple studies in human cell lines and mouse models that identified USP22 as a negative regulator of type I-III interferon signaling and interferon-stimulated genes (ISGs)^49–53^. Pathways commonly depleted in FHSC14 and NCI109B sgUSP22 models included cell-cell junction and cell migration, and neuronal pathways such as Nervous System Development, Central Nervous System and Brain Development (Fig. 4c, d). Neuronal gene sets were amongst the top depleted pathways in both *USP22* deletion PDX models (Supplementary Fig. 4c–f) where top downregulated proteins in FHSC14 sgUSP22 tumors included neuronal pathway proteins such as NKX2-2, ROBO1, ROBO2, SEMA6A, FABP7, and PCDHB10 (Fig. 4a).

### USP22 regulates transcription of neuronal/neuroendocrine and DNA damage associated genes

**Fig. 5:**
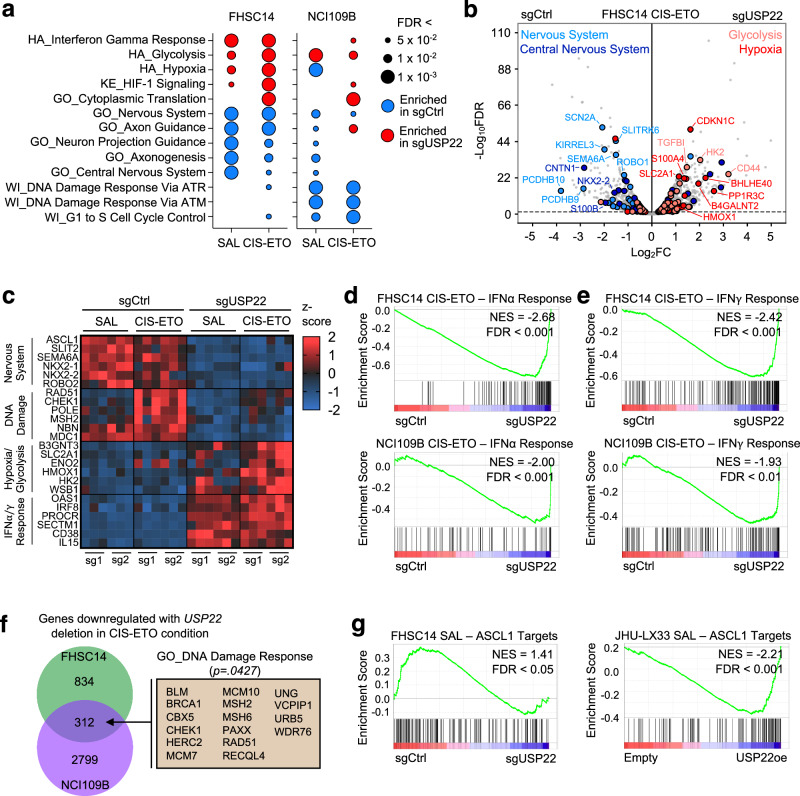
RNA-seq analysis of SCLC PDX models with *USP22* perturbation. **a** Differentially expressed upregulated or downregulated genes (FDR < 0.05) from RNA-seq data in FHSC14 USP22-sg1/2 vs Ctrl-sg1/2 (*n* = 3 tumors per sgRNA for a total of *n* = 6 tumors per group) and NCI109B sgUSP22 vs sgCtrl tumors models (*n* = 5 tumors per group) treated for 48 h with saline or cis-eto were independently queried for pathway enrichment using Enrichr. Pathways from Gene Ontology Biological Process 2023 (GO), MSigDB Hallmark 2020 (HA), KEGG 2021 Human (KE), WikiPathways 2024 Human (WI) gene set databases commonly enriched in or depleted in at least 2 out of 3 PDX models with respect to USP22 status are displayed (JHU-LX33 USP22-PLVX vs Empty-PLVX PDX pathway analysis data in Supplementary Fig. 5a) (see Supplementary Data 9–12 for all enriched or depleted pathways). Point size scaled to FDR. **b** Volcano plot of RNA-seq differential gene expression analysis from 48 h cis-eto-treated FHSC14 USP22-sg1/2 vs Ctrl-sg1/2 models. Genes belonging to the indicated pathways Gene Ontology Biological Process 2023 and MSigDB Hallmark 2020 pathways are displayed in blue and red, respectively. **c** Heatmap of RNA-seq data for select FHSC14 USP22-sg1/2 vs Ctrl-sg1/2 differentially expressed genes from the indicated pathways. Each column is an individual tumor. GSEA analyses of Hallmark Interferon Alpha Response (**d**) and Interferon Gamma Response (**e**) pathways in FHSC14 and NCI109B sgUSP22 vs sgCtrl tumors treated for 48 h with cis-eto. **f** Enrichr pathway analysis of genes commonly downregulated in FHSC14 and NCI109B sgUSP22 tumors treated for 48 h with cis-eto. **g** GSEA analyses of custom ASCL1 Targets gene set^61^ in FHSC14 sgUSP22 vs sgCtrl and JHU-LX33 USP22-PLVX vs Empty-PLVX. Adjusted Enrichr analysis *p* values are shown on figures.

The SAGA complex in which USP22 participates plays a key role in gene transcription. We thus complemented our proteomic analyses with RNA-seq analyses of FHSC14 USP22-sg1/2 vs sgCtrl-sg1/2 and NCI109B sgUSP22 vs sgCtrl tumors after 48 h of either cis-eto or saline control treatment (Fig. 5a–c). In addition, we transcriptionally profiled JHU-LX33 USP22-PLVX vs Empty-PLVX tumors that had been treated for 10 days with either cis-eto or a saline control (Supplementary Fig. 5a, b). Differentially expressed genes were queried using Enrichr to identify common pathways altered as a function of *USP22* status in at least 2 out of 3 PDX models tested (FDR < 0.05; Fig. 5a–c, Supplementary Fig. 5a). Supplementary Data 9–12 provide the complete list of all significantly enriched or depleted pathways. Consistent with our proteomic analyses, the Interferon Gamma Response pathway gene expression was upregulated in *USP22*-deleted FHSC14 and NCI109B tumors (Fig. 5a, c). Gene set enrichment analysis (GSEA)^54^ also confirmed strong enrichment of the Interferon Alpha Response (Fig. 5d) and Interferon Gamma Response pathways (Fig. 5e) in both cis-eto-treated USP22 sgRNA-expressing PDX models. USP22 loss also increased activation of pathways related to hypoxia and glycolysis (Fig. 5a–c, Supplementary Fig. 5a, b) and suppressed expression of DNA damage response transcripts (Fig. 5a, f). Multiple pathways related to nervous system development were also consistently suppressed upon USP22 loss (Fig. 5a–c, Supplementary Fig. 5a, b).

Among FHSC14 genes downregulated following USP22 loss was *ASCL1*, a transcription factor and master regulator of SCLC neuroendocrine transcriptional programs, together with other regulators of neuronal differentiation and development including *INSM1*, *NXK2-1*, *NKX2-2*, *ROBO2*, and *SEMA6A*^55–60^ (Fig. 5c). GSEA further confirmed suppression of ASCL1 target genes^61^ in FHSC14 sgUSP22 versus sgCtrl tumors, and upregulation of ASCL1 target genes in JHU-LX33 USP22-expressing tumors relative to empty vector control (USP22 null) tumors (Fig. 5g). Of note, resistance to platinum-based therapies in SCLC has been associated with reduced ASCL1 expression, enrichment of ASCL1-low cells in relapsed samples, and neuroendocrine to non-neuroendocrine fate switching^6,9,20,22^.

Some phenotypes resulting from USP22 perturbation have been proposed to be mediated by deubiquitylation of non-histone substrates^41–43^. Thus, we mined our global proteomics and RNA-seq datasets to identify cases with depletion at the protein level upon USP22-deletion in PDX models but not the RNA level (Supplementary Fig. 6a–c, Supplementary Data 13–14). While this analysis did not yield hits that could readily be linked to chemoresistance, interestingly, we observed SAGA deubiquitylase components ATXN7L3 and ATXN7L2 were depleted at the protein level with no change at the RNA level in the NCI109B model (Supplementary Fig. 6b). These data suggest a destabilization effect of the SAGA deubiquitylase module with loss of USP22. Furthermore, USP27X, a related deubiquitylase that competes with USP22 to bind with ATXN7L3 and ENY2 to form DUB modules independent SAGA^62^, was also commonly depleted at the protein level in both USP22-null FHSC14 and NCI109B PDX models (Supplementary Fig. 6c).

### USP22 regulates neuronal gene expression by modulating histone H2A ubiquitylation in SCLC

Within SAGA, USP22 is thought to regulate gene expression in part via deubiquitylation of ub-H2A K119 (H2AK119ub) and ub-H2B K120 (H2BK120ub)^30,37^. Transcriptional repression of specific genes is mediated through recruitment of Polycomb repressive complexes 1 and 2 (PRC1/2) to deposit H2AK119ub and H3K27me3, respectively^63^ where these histone marks overlap at genomic loci due to co-recruitment of PRC1 and PRC2^64,65^. USP22 can reverse repression of these genes by deubiquitylation of H2AK119ub^30^, which displaces PRC2 with the loss of H3K27me3 deposition^66^. Thus, failure to deubiquitinate H2AK119ub may result in improper gene silencing.

**Fig. 6:**
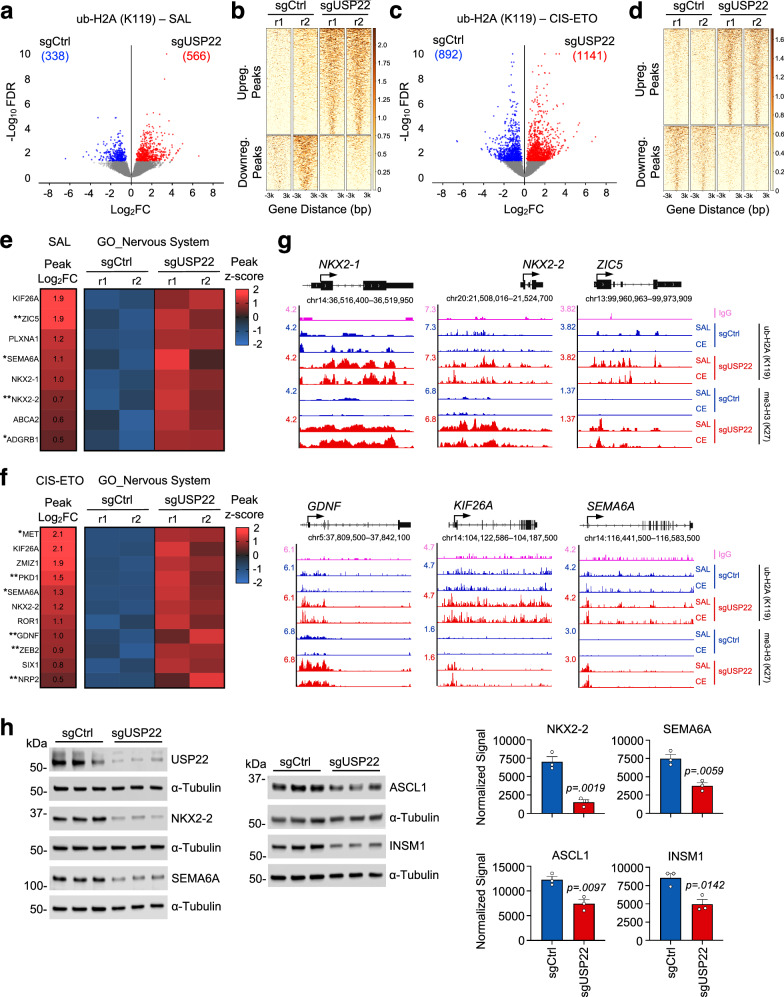
USP22 regulates histone H2A ubiquitylation at neuronal genes in SCLC. **a**, **b** Volcano plot of CUT&Tag data for H2AK119ub differential peak analysis from in FHSC14 sgUSP22 vs sgCtrl models (*n* = 2 tumors per group). The numbers of differentially enriched peaks (FDR < 0.05) in sgUSP22 (red) and in sgCtrl (blue) are shown (**a**). Heatmaps depicting upregulated and downregulated H2AK119ub peaks. Heatmaps show data 3 kb upstream and downstream of peak center (**b**). Volcano plots (**c**) and heatmaps (**d**) of CUT&Tag data for H2AK119ub differential peak analysis in FHSC14 sgUSP22 vs sgCtrl models treated for 48 h with cis-eto. **e**, **f** Heatmaps of FHSC14 sgUSP22 vs sgCtrl H2AK119ub peak Log_2_FC and z-scores for transcriptionally downregulated genes with increased H2AK119ub within the GO Central Nervous System Development (no asterisk), GO Nervous System Development, (*) or both pathways (**). **g** IGV images of FHSC14 sgUSP22 vs sgCtrl CUT&Tag H2AK119ub and H3K27me3 peak data for select Central Nervous System Development and Nervous System Development genes in (**e** and **f**). **h** Western blot analyses of FHSC14 sgUSP22 vs sgCtrl tumors. Each lane is an independent tumor (*n* = 3 tumors per group), with each blot representative from two independent experiments. Quantification of protein signal normalized to loading control is shown for each condition. Data are means ± SEM. Statistical analyses in **h** were performed by unpaired student’s two-sided *t* test. Unadjusted *t* test *p* values are shown on figures. Source Data is provided in the Source Data File.

To identify molecular mechanisms regulating transcriptional changes upon *USP22* deletion, we performed Cleavage Under Targets and Tagmentation (CUT&Tag)^67^ for H2AK119ub and H3K27me3. Following a 48-h treatment with saline or cis-eto, FHSC14 sgCtrl and sgUSP22 tumors were dissociated, viable cells sorted by FACS then subjected to CUT&Tag. In saline-treated sgUSP22 tumors, 566 H2AK119ub peaks (324 unique protein-coding genes) were gained while 338 peaks (160 unique protein-coding genes) were lost relative to sgCtrl tumors (Fig. 6a, b) (Supplementary Data 15). This increase in H2AK119ub peaks in *USP22*-null tumors is consistent with USP22 acting as an H2AK119 deubiquitinase, although secondary effects of USP22 loss may also contribute. We found that global levels of H2AK119 ubiquitylation were unchanged in 3 PDX models with *USP22* deletion, as assessed by western blot (Supplementary Fig. 7a–c). A total of 1141 H2AK119 peaks 652 unique protein-coding genes were gained, while 892 peaks (413 unique protein-coding genes) were lost in sgUSP22 tumors treated with cis-eto relative to sgCtrl tumors (Fig. 6c, d). In saline and cis-eto-treated sgUSP22 tumors combined, 86 out of 722 unique protein-coding genes with increased H2AK119ub were concurrently transcriptionally downregulated (Supplementary Data 16). Of these 86 genes, 16 belonged to the Nervous System Development or Central Nervous System Development pathways (Fig. 6e, f). Among these 16 neuronal genes in sgUSP22 tumors, H2AK119ub increased, and RNA expression decreased for *NXK2-1*, *NKX2-2*, *ZIC5*, *GDNF*, *KIF26A*, and *SEMA6A*. We also observed increases in H3K27me3 peaks at these loci relative to sgCtrl tumors (Fig. 6g), consistent with strong epigenetic repression. Western blotting of NKX2-2 and SEMA6A in FHSC14 tumors confirmed decreased protein expression in sgUSP22 relative to sgCtrl tumors (Fig. 6h). In accordance with RNA downregulation of ASCL1 targets in *USP22*-null tumors (Fig. 5d), we observed a decrease in ASCL1 and INSM1 protein levels as assessed by western blotting and IHC in *USP22*-deleted FHSC14 tumors (Fig. 6h, Supplementary Fig. 8a–d). Furthermore, in the NEUROD1-high expressing PDX model NCI109B, both NEUROD1 and INSM1 protein levels were decreased in tumors with *USP22* deletion (Supplementary Fig. 8b, e). Taken together, these data suggest that USP22 may directly regulate the expression of neuronal genes in SCLC at the epigenetic level by removing ubiquitin from H2AK119. Furthermore, deletion of *USP22* resulted in increased H2AK119ub and H3K27me3 at these genomic loci, correlating with transcriptional repression of neuronal genes in SCLC.

### SCLC PDX models with *USP22* deletion exhibit blunted DNA damage signaling in response to chemotherapy

In our RNA-seq analyses, we observed suppression of multiple DNA damage response pathways in FHSC14 and NCI109B sgUSP22 tumors prior to or after 48 h of cis-eto treatment relative to control sgCtrl tumors (Fig. 5a, c, f). Among the differentially expressed genes identified were identified 312 genes downregulated upon USP22 deletion that were shared between cis-eto-treated FHSC14 and NCI109B (Fig. 5f). Pathway analysis on these genes revealed the DNA IR Damage and Cellular Response via ATR (WikiPathway, WP4016) transcripts, such as *CHEK1*, *RAD51*, *BRCA1*, *MSH2*, that were suppressed in both USP22 KO FHSC14 and NCI109B (Fig. 5f). In FHSC14 control tumors, transcript levels of multiple DNA damage response (DDR) genes increased robustly upon cis-eto treatment versus suppressed expression of these DNA damage response genes after treatment in FHSC14 sgUSP22 tumors (Fig. 5c).

**Fig. 7:**
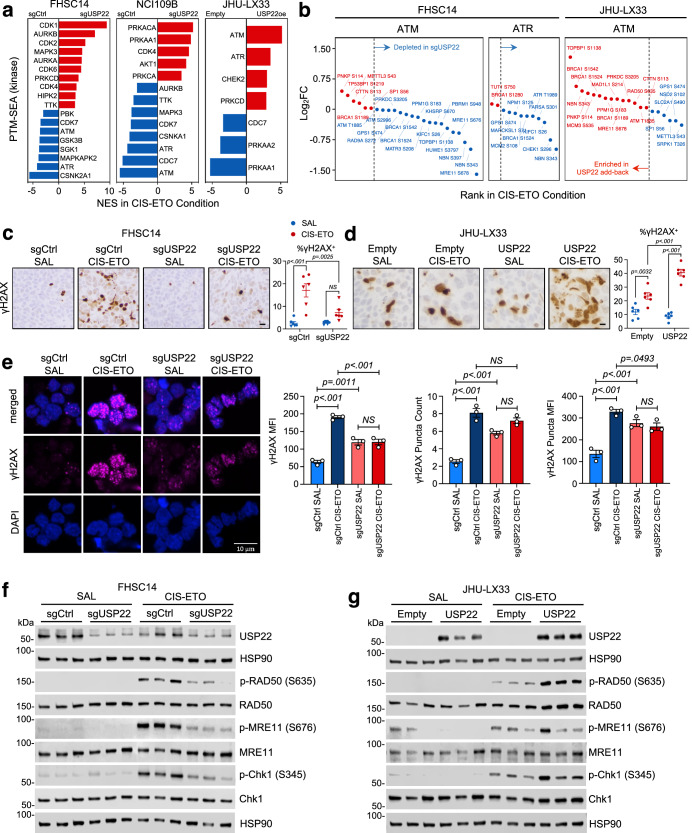
*USP22* deletion results in decreased neuronal protein expression and blunted cis-eto-induced DNA damage signaling in SCLC PDX models. **a** Phospho-proteomics analyses of FHSC14 USP22-sg1/2 vs Ctrl-sg1/2 (*n* = 2 tumors per sgRNA, *n* = 4 tumors per group), NCI109B sgUSP22 vs sgCtrl (*n* = 3 tumors per group), and JHU-LX33 USP22-PLVX vs Empty-PLVX models (*n* = 4 tumors per group) treated for 48 h with cis-eto and analyzed by PTM signature enrichment analysis (PTM-SEA). Shown are significant kinase signatures (FDR < 0.05) comparing control to USP22-perturbed tumors for each model. **b** ATM/ATR kinase signature phosphosite Log_2_ fold changes for cis-eto treated FHSC14 and JHU-LX33 with respect to USP22 status. IHC analyses and representative images for γH2AX in FHSC14 sgCtrl vs sgUSP22 tumors treated for 48 h with saline or cis-eto (**c**), or in JHU-LX33 Empty-PLVX and USP22-PLVX tumors treated for 10 days with saline or cis-eto (**d**). Data are means ± SEM, *n* = 6 tumors per group. Scale bar, 10 µm. **e** IF quantification and representative images of γH2AX and DAPI for explanted cells from FHSC14 sgCtrl and sgUSP22 tumors treated in vivo with saline or cis-eto for 48 h. Data are means ± SEM; *n* = 3 tumors per group. Scale bar, 10 µm. Western blot analyses of FHSC14 sgCtrl vs sgUSP22 tumors (**f**) or JHU-LX33 Empty-PLVX vs USP22-PLVX tumors (**g**). Each lane is an independent tumor (*n* = 3 tumors per condition), with each blot representative from three independent experiments. Statistical analyses in (**c–e**) were performed by 1-way ANOVA with Tukey’s multiple comparisons test. Adjusted ANOVA *p* values are shown on figures; NS non-significant. Source Data is provided in the Source Data File.

Activation and maintenance of the DNA damage response is mediated through rapid and dynamic phosphorylation cascades involving DNA damage sensors, signal transducers, repair machinery, and cell cycle checkpoint kinases^68^. Both FHSC14 and NCI109B sgUSP22 models exhibited reduced expression of DDR genes relative to sgCtrl models treated with cis-eto (Fig. 5a–c). Phosphorylation events are critical for DDR signaling. Thus, we next performed phospho-proteomics profiling of tumors after 48 hrs of cis-eto or control treatment of FHSC14 USP22-sg1/2 vs Ctrl-sg1/2, NCI109B sgUSP22 vs sgCtrl and JHU-LX33 USP22-PLVX vs Empty-PLVX (Supplementary Data 17–19). Genes whose phosphorylation sites were enriched (Log_2_FC > 0.5, FDR < 0.05) or depleted (Log_2_FC < −0.5, FDR < 0.05) in cis-eto-treated sgUSP22 vs sgCtrl tumors were queried for pathway enrichment using Enrichr. After cis-eto treatment, multiple DDR pathways were depleted in *USP22*-deleted FHSC14 and NCI109B models (Supplementary Fig. 9a, b). DDR pathways were conversely enriched in upon USP22 restoration in JHU-LX33 (Supplementary Fig. 9c). Phosphosite-specific signature enrichment analysis (PTM-SEA)^69^ of all three models allowed the inference of kinase activity based on known substrate phosphorylation. In the cis-eto-treated samples of FHSC14 and NCI109B, both ATM and ATR exhibited reduced inferred activity upon USP22 loss, with increased activity following USP22 addback to JHU-LX33 (Fig. 7a, b, Supplementary Fig. 9d, e). These data indicate that USP22 loss can reduce DDR phospho-signaling upon cis-eto treatment.

H2AX is phosphorylated at Serine 139 (γH2AX) at DNA break sites by ATM and ATR, serving to recruit DNA repair factors^70–72^. We performed γH2AX IHC analyses on FHSC14 and NCI109B USP22-sg1/2 vs Ctrl-sg1/2 treated for 48 h with cis-eto or saline as well as JHU-LX33 USP22 add-back vs empty vector control treated for 10 days. While cis-eto increased the number of γH2AX^+^ cells in Ctrl-sg1/2 tumors, the impact of cis-eto on γH2AX^+^ cells was significantly reduced in both USP22-sg1/2 FHSC14 (Fig. 7c) and NCI109B (Supplementary Fig. 9f) models. In the JHU-LX33 model, both empty vector control and USP22 add-back tumors exhibited an increase in γH2AX^+^ cells in response to cis-eto; however, this increase was reduced in the empty vector control tumors relative to the USP22 add-back tumors (Fig. 7d). To further assess the impact of USP22 loss on γH2AX foci formation, we performed immunofluorescence (IF) analysis on FHSC14 sgCtrl and sgUSP22 tumors cells treated in vivo with saline or cis-eto for 48 h. In sgCtrl tumors, cis-eto led to a robust increase in number of *γ*H2AX puncta per cell, puncta MFI and total MFI per cell relative to saline treatment (Fig. 7e, Supplementary Fig. 9g). The impact of cis-eto on γH2AX was significantly reduced in sgUSP22 tumors. In line with decreased cis-eto-induced γH2AX puncta, western blot analyses in FHSC14 reveal decreased phosphorylation of downstream ATM/ATR kinase DDR targets, including phospho-RAD50 (S635), phospho-MRE11 (S676), and phospho-Chk1 (S345) in cis-eto sgUSP22 tumors relative to cis-eto-treated sgCtrl tumors (Fig. 7f). Basal protein levels of RAD50, MRE11, and Chk1 remain unchanged across genotypes and treatment conditions. Again, adding back USP22 in JHU-LX33 increased the abundance of cis-eto-induced phosphorylation of these 3 DDR proteins (Fig. 7g). Taken together, these data show USP22-null SCLC tumors exhibit strikingly reduced DNA damage responses to chemotherapy.

### Treatment-dependent upregulation of glycolytic gene expression in response to USP22 loss: a targetable therapeutic vulnerability

**Fig. 8:**
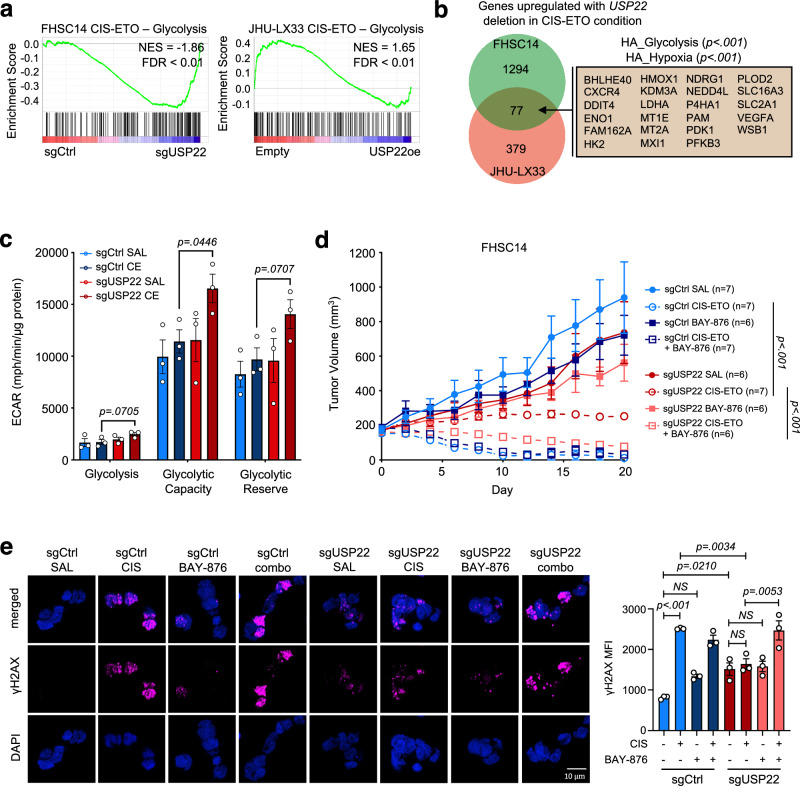
Glycolytic gene expression upregulated in response to cis-eto treatment in PDX models with *USP22* deletion: a targetable therapeutic vulnerability. **a** GSEA analyses of Hallmark Glycolysis gene set in FHSC14 sgUSP22 vs sgCtrl tumors treated for 48 h with cis-eto and JHU-LX33 USP22-PLVX vs Empty-PLVX tumors treated for 10 days with cis-eto. **b** Enrichr pathway analyses of genes upregulated in both FHSC14 sgUSP22 and JHU-LX33 Empty-PLVX (USP22-null) tumors relative to control tumors in the cis-eto conditions. **c** Seahorse glycolytic stress test assay data for explanted tumor cells from FHSC14 sgCtrl and sgUSP22 models treated in vivo with saline or cis-eto for 48 h. Data are means ± SEM; *n* = 3 tumors per group. **d** Tumor volume over time growth curves of isogenic FHSC14 sgCtrl and sgUSP22 models treated with saline, cis-eto, GLUT1 inhibitor BAY-876, or combination over a 20-day period. Data are means ± SEM; exact number (n) of animals per group is shown on figure. **e** IF quantification and representative images of *γ*H2AX and DAPI from FHSC14 sgCtrl and sgUSP22 tumor cells treated in vitro with saline, 1 μM cisplatin, 0.1 μM BAY-876, or combination for 48 h. Data are means ± SEM; *n* = 3 tumors per group. Scale bar, 10 µm. Statistical analyses in (**c**) were performed by unpaired student’s two-sided t-test. Statistical analyses in (**d**) were performed by 2-way ANOVA with Tukey’s multiple comparisons test. Statistical analyses in (**e**) were performed by 1-way ANOVA with Tukey’s multiple comparisons test. Unadjusted *t* test *p* values, adjusted ANOVA *p* values and adjusted Enrichr analysis *p* values are shown on figures; NS non-significant. Source Data is provided in the Source Data File.

Gene expression analyses of the *USP22*-deleted PDX models FHSC14 and NCI109B identified upregulation of glycolysis and hypoxia/HIF-1 pathways upon cis-eto treatment, and the absence of these responses in USP22-expressing JHU-LX33 tumors (Fig. 5a–c, Supplementary Fig. 5a, b). GSEA further revealed enrichment of Glycolysis pathways in cis-eto-treated FHSC14 sgUSP22 and JHU-LX33 empty vector control (*USP22*-null) tumors relative to USP22-expressing tumors (Fig. 8a). Pathway analysis on 77 common genes upregulated in cis-eto-treated *USP22*-null FHSC14 and JHU-LX33 models further revealed strong enrichment of Glycolysis and Hypoxia-related transcripts (Fig. 8b). Key genes upregulated upon USP22 loss in FHSC14 and JHU-LX33 models included the GLUT1 glucose transporter encoded by *SLC2A1*, and hexokinase 2 encoded by *HK2* that catalyzes the conversion of glucose to glucose-6-phosphate (Fig. 5b, c, Fig. 8b, Supplementary Fig. 5b).

To determine whether upregulation of glycolytic gene expression in USP22-null PDX models alters glycolytic metabolism, we treated FHSC14 sgCtrl and sgUSP22 tumors with saline or cis-eto for 48 h (*n* = 3 tumors per treatment condition), dissociated these tumors into single-cell suspensions and subjected the cells to a Seahorse glycolytic stress test assay. Glycolytic metrics were unchanged between sgCtrl vs. sgUSP22 tumors at baseline (Fig. 8, Supplementary Fig. 10a). Cis-eto treatment did not alter glycolytic metrics in sgCtrl tumors but led to a significant increase in glycolytic capacity in *USP22*-null tumors while increases in glycolytic reserve in sgUSP22 tumors trended towards significance (Fig. 8c, Supplementary Fig. 10a). Taking together, gene expression and functional glycolytic stress results, support loss of USP22 in SCLC PDX models activating glycolytic metabolic programs during chemotherapy treatment.

Given the role for glycolysis in driving platinum resistance in other tumor types^73–79^, we next asked if inhibiting glycolysis would re-sensitize *USP22*-null SCLC models to chemotherapy. To test this hypothesis, we treated FHSC14 sgCtrl and sgUSP22 tumors with saline, cis-eto, the GLUT1 inhibitor BAY-876^80^, or combinations over a 3 week time course (Fig. 8d, Supplementary Fig. 10b). Both FHSC14 sgCtrl and sgUSP22 tumors grew at comparable rates during saline or BAY-876 treatment. Cis-eto treatment led to near complete ablation of sgCtrl tumors, while sgUSP22 tumors exhibited partial resistance and continued to grow for 10 days with static growth for another 10 days. In contrast, BAY-876 treatment restored cis-eto sensitivity to sgUSP22 tumors, where tumor volume significantly decreased with tumor growth rates closely mimicking sgCtrl control tumors treated with cis-eto alone (Fig. 8d, Supplementary Fig. 10b). These results suggest that inhibiting glycolytic metabolism may represent a targetable vulnerability in USP22-null PDX models leading to the restoration of chemotherapeutic sensitivity.

To understand how suppression of glycolysis can re-sensitize USP22-null SCLC PDX models to chemotherapy, we performed γH2AX IF analysis on dissociated FHSC14 sgCtrl and sgUSP22 tumor cells treated with vehicle, cisplatin, BAY-876, or combination in vitro for 48 h. Consistent with our in vivo experiments, in vitro treatment with cisplatin led to a robust increase in *γ*H2AX cell MFI in sgCtrl but not sgUSP22 cells (Fig. 8e, Supplementary Fig. 10c). However, combining cisplatin with GLUT-1 inhibitor BAY-876 increased cisplatin-induced *γ*H2AX signal in sgUSP22 cells to levels similar to that of combination-treated sgCtrl cells (Fig. 8e). Taken together, these data show that suppression of glycolysis by targeting GLUT1 can increase chemotherapy-induced DNA damage signaling and re-sensitize *USP22*-null SCLC tumors to chemotherapy.

**Fig. 9:**
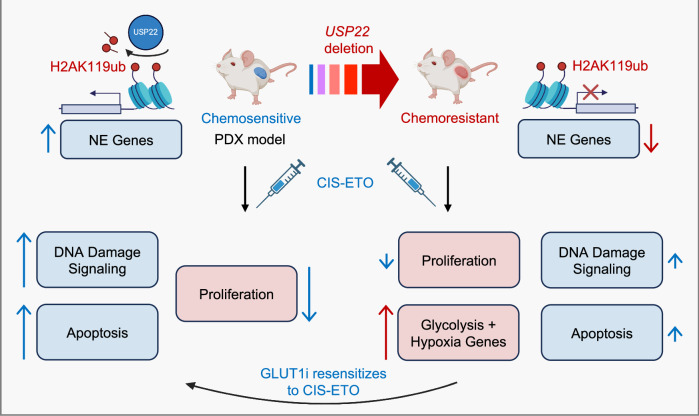
Schematic diagram showing the effects of USP22 loss on the cellular response to CIS-ETO treatment in SCLC PDX models. Created in BioRender. Best, S. (2026) https://BioRender.com/22p9q6j.

The results from this study collectively demonstrate that loss of the USP22 deubiquitylase profoundly alters transcription and protein phosphorylation across PDX models of SCLC, with suppression of neuronal/neuroendocrine programs, activation of glycolytic programs, and the suppression of DNA damage-driven protein phosphorylation responses leading to continued tumor growth through chemotherapy (Fig. 9).

## Discussion

We have developed and used in vivo CRISPR-Cas9-based functional deletion screens in chemosensitive SCLC PDX models to identify genes that govern the response to the SCLC standard-of-care chemotherapy. The reproducible enrichment of sgRNAs targeting multiple members of the SAGA complex in cis-eto-treated SCLC PDX tumors led us to investigate the importance of the SAGA complex USP22 deubiquitylase in SCLC chemoresistance. We perturbed USP22 expression in three SCLC PDX models where deletion of *USP22* switched chemosensitive FHSC14 and NCI109B PDX models to become chemoresistant. In contrast, re-expressing USP22 in the *USP22*-null, chemoresistant JHU-LX33 PDX model partially re-sensitized the tumors to chemotherapy. Of note, a point mutant of USP22 that abolished deubiquitylase activity failed to re-sensitize *USP22*-null chemoresistant JHU-LX33 tumors.

Subsequent functional analyses of USP22 further supported the notion that USP22 deubiquitylation activity within SAGA is an important determinant of SCLC chemotherapeutic response in major transcriptional subsets of SCLC represented by ASCL1-high (FHSC14) and NEUROD1-high (NCI109B and JHU-LX33) PDX models. Other mouse models support the role of USP22 tumor suppressor activity in different tumor types, where deletion of *Usp22* sensitized models of colon cancer^81^ and myeloid leukemia^82^ with accelerated tumorigenesis. While we did not find evidence of a growth advantage in the absence of chemotherapy upon *USP22* deletion, the observation of a homozygous *USP22* inactivating mutation in the PDX model JHU-LX33, derived from chemonaïve SCLC^83^ suggests possible tumor suppressor function even in the absence of chemotherapy. The vast majority of whole genome and exome sequencing data in SCLC are from untreated samples, and it remains to be determined whether SAGA complex members are more frequently inactivated in treatment relapsed SCLC. Of note, USP22 and other SAGA complex members are not included in most targeted capture panels that have revealed new alterations in therapy relapsed SCLC^84^. More detailed genomic analyses of SCLC post-treatment patient samples will be important to determine if mutations or deletion of *USP22* or other SAGA components are enriched in chemotherapy-relapsed SCLC.

Our results provide clues to the mechanisms by which USP22 as a SAGA complex deubiquitylase modulates gene and protein expression, tumor growth, and therapeutic response. SAGA is known to have modular activities that regulate gene activation and transcription. For example, USP22 removes ubiquitin at the histone site H2AK119^30^, a repressive mark deposited by PRC1 that is often co-localized with H3K27me3 to promote gene silencing^63–66^. USP22 can also remove ubiquitin from H2BK120^37^ to promote transcription elongation^31,40^. In addition to the NEUROD1-expressing PDX model NCI109B, SAGA complex members including *USP22* were top hits in functional screens in the ASCL1-high model FHSC14^25^. We observed a stronger magnitude of resistance to chemotherapy upon USP22 deletion in the FHSC14 ASCL1-high model over the NEUROD1-high NCI109B model, however, whether this relates to ASCL1 vs NEUROD1 expression or to other biological differences between these models remains to be determined. Despite differences in the magnitude of the effects in the different PDX models tested, *USP22* deletion appears to be able to contribute to chemoresistance across different SCLC subtypes.

Several consequences of *USP22* deletion in SCLC PDX models may contribute to an impaired response to chemotherapy. For example, deleting *USP22* in FHSC14 and NCI109B PDX models suppressed cis-eto-induced apoptosis, an effect that could be reversed by re-expressing USP22 in the USP22-null JHU-LX33 PDX model. USP22 loss also reduced the transcriptional upregulation of DDR repair genes in response to chemotherapy and the phosphorylation of canonical ATM/ATR kinase targets. Activation of ATM/ATR can induce cell death through p53-dependent and independent mechanisms, and suppression of these downstream pathways upon USP22 loss may decrease cis-eto-induced cell death, as we observed in treated PDX models.

SCLC exhibit differing levels of activation of neuroendocrine transcriptional programs. In murine SCLC GEMM models and cell lines, low-neuroendocrine cells generated through constitutive NOTCH activation were less sensitive to platinum chemotherapy relative to neuroendocrine cells^22^. In circulating tumor cell-derived xenograft models of SCLC, development of platinum resistance correlated with a decrease in ASCL1-expressing cells^9^. Furthermore, studies of tumor samples taken from relapsed SCLC patients show reduced ASCL1 expression and enrichment of ASCL1-low subtypes relative to treatment-naïve samples^6,20^. In our study, RNA and protein levels for markers of neuroendocrine differentiation ASCL1 and INSM1 were reduced by *USP22* deletion in FHSC14, and ASCL1-target gene sets were depleted in USP22-null FHSC14 and JHU-LX33 models. Reduced expression of neuronal genes at the transcriptomic and proteomic level was associated with USP22 loss in one ASCL1-high PDX model FHSC14 and two NEUROD1-high models NCI109B and JHU-LX33. A potential mechanistic explanation for these effects is provided by the observation that *USP22* deletion increased H2AK119ub levels in association with increased H3K27me3 deposition in gene bodies, leading to the decreased expression of neuronal and neuroendocrine differentiation programs. However, we note that with constitutive *USP22* deletion it can be challenging to discern direct from indirect effects of USP22 loss. Future studies to link USP22 genomic occupancy to sites of altered H2A-K119ub and testing the impact of modulating H2A-K119ub on observed phenotypes would help to overcome this limitation to the study. One additional intriguing finding was increased expression of glycolysis and hypoxia-related genes, where activation of these metabolic programs has been linked to platinum resistance in multiple tumor types^73–79^. Prior work revealed that inhibition of glycolysis with 2-deoxy-D-glucose in combination with cisplatin could overcome platinum resistance in head and neck cancer cells^76^, gastric cancer cells^74^, and neuroblastoma cells^85^. We found that activation of glycolytic programs at the transcriptional level in chemoresistant *USP22*-null SCLC models was a targetable vulnerability, where inhibition of GLUT1 increased cis-eto-induced *γ*H2AX phosphorylation re-sensitized tumors to chemotherapy. Identifying the precise mechanisms by which USP22 regulates glycolysis will be important to further develop this potential therapeutic opportunity.

Our results identify the SAGA complex USP22 deubiquitylase as an important determinant of chemotherapy responsiveness in SCLC. Our global proteomic, phospho-proteomic, transcriptomic, and histone modification analyses further reveal multiple histone and non-histone targets of USP22, and detail pleiotropic consequences that follow *USP22* deletion. These include upregulation of interferon and glycolytic pathways, suppression of neuronal and ASCL1 targets, and suppressed DNA damage responses and apoptosis. We found further glycolytic program activation was functionally important and may provide a therapeutic opportunity in SCLC: GLUT1 inhibition was able to resensitize USP22-deleted SCLC PDX tumors to cis-eto chemotherapy. Our results thus provide a rich data set and useful PDX models for further investigating the functional roles and mechanistic pathways that regulate chemotherapeutic response in SCLC.

## Methods

### Ethics statement

The animal procedures conducted in this study were approved by the Institutional Animal Care and Use Committee (IUCUC) at Fred Hutch Cancer Center (protocol #50783).

### Mice

Male and female NOD *scid* gamma mice (NSG, JAX strain 005557) immunodeficient mice ages 8–24 weeks were obtained from Translational Research Model Services at Fred Hutch Cancer Center (FHCC). Mice were housed with no more than 5 animals per cage in a temperature and humidity controlled environment with a standard 12-h dark/light cycle. Sample sizes for each experiment are provided in the Figures or Figure Legends. Sex was not considered in the study design, and experiments were performed using either female or male mice, with each individual experiment performed in a single sex. Mice were randomly assigned to treatment groups until sample sizes per group were met. The maximum permitted tumor burden for mouse studies was no greater than an average measurement of 2 cm tumor length x 2 cm tumor width. No mice exceeded the maximum permitted tumor burden at any point during these studies.

### Patient derived xenograft (PDX) models

The following PDX models were used in this study: FHSC14, NCI109B, and JHU-LX33. NCI109B was obtained from the NCI Patient-Derived Models Repository (distribution lot 594431-109-B). JHU-LX33 was provided by Dr. Charles Rudin (Memorial Sloan Kettering). All PDX models were propagated by mixing cells with Matrigel at a 1:1 v/v ratio and injected subcutaneously in the flanks of NSG mice. To generate single-gene perturbed PDX models, tumors were dissociated into single-cell suspensions, digested in 1 mg/mL collagenase type IV at 37 °C for 30 min, cleared of red blood cells using ACK Lysing Buffer (ThermoFisher, A1049201), and infected ex vivo with concentrated lentivirus containing lentiCRISPRv2-ZsGreen vectors with Ctrl-sg1, Ctrl-sg2, USP22-sg1, or USP22-sg2 gRNAs (see Supplementary Data 5 for sgRNA sequences) or lentivirus containing Empty, USP22, or USP22-C185S PLVX ZsGreen vectors in the presence of 4 μg/mL polybrene. PDX models were cultured in DMEM-F12 (Gibco, 11320-033) supplemented with 10% FBS, 1% Pen-Strep (Gibco, 15140-122), and 1% Insulin-Transferring Selenium (Gibco, 41400-045). No more than 18 h after infection, cells were washed and injected into NSG mice flanks. Once regrown, tumors were again dissociated into single-cell suspension and successfully transduced ZsGreen^+^ cells were purified via FACS and re-implanted into mice with rounds of reimplantation and cell sorting until the vast majority of cells were ZsGreen + . *USP22* deletion in FHSC14, NCI109B or USP22 overexpression in JHU-LX33 was confirmed by western blot.

### Drug treatments

For mouse drug treatment experiments, flank tumor dimensions were measured with calipers daily or every other day and tumor volume was calculated by volume=(major axis)×(minor axis)^2^/2. Saline (VetOne, 510224) was administered by intraperitoneal (IP) injection once a week. Cisplatin (5 mg/kg, Millipore Sigma, 479306) + etoposide (10 mg/kg, Millipore Sigma, E1383) treatments were administered by IP injections on a weekly cycle with the following schedule: Day 1 = cisplatin + etoposide, Day 2 = etoposide, Day 3 = etoposide, Days 4–7 = drug holiday. BAY-876 (3 mg/kg, MedChemExpress, HY-100017) was administered by oral gavage daily.

### Cell lines

293TN producer cells (System Bioscience, LV900A-1) were used for lentivirus production. Cells were cultured in DMEM containing 10% FBS and 1% Pen-Strep (DMEM-10) in a humidified incubator at 37 °C, 95% air, and 5% CO_2_.

### Lentivirus production and concentration

To produce concentrated lentivirus, 293TN cells were seeded onto 0.01% poly-L-lysine (Sigma Aldrich, P4832) coated 500 cm^2^ plates (Corning, 431111) at a density of 15×10^6^ cells per plate. A transfection mix containing 137.5 μg transfer vector, 137.5 μg psPAX2 (Addgene, 12260), and 90 μg pMD2.G (Addgene, 12259), 1.14 mL 2 M CaCl_2_ (Quality Biological, 351-130-721), and 4.75 mL 2X HBS buffer in 90 mL DMEM-10 was applied to cells once reaching 75% confluency. 16 h post-transfection, media was changed to Viral Producing Media (VPM): DMEM, 1% FBS, 1% Pen-Strep, 1% 100 mM Sodium Pyruvate (Gibco, 11360-070), 1% Sodium Bicarbonate 7.5% solution (Corning, 25-035-Cl), 1% 0.5 M Sodium Butyrate solution, and 2 μg/mL insulin human recombinant zinc (Gibco 12585-014). 64 h post-transfection, lentivirus-containing supernatant was filtered through 0.45 μm Millipore low-protein binding filter units (Millipore Sigma, S2HVU02RE) and concentrated via centrifugation at 3300 x *g* for 30–45 min at 4 °C using Millipore Centricon 70 Plus cartridges (ThermoFisher, UFC710008).

### Western blot

Protein was extracted from snap-frozen tumor tissue by mechanical disruption and homogenization using the Agile Tissue Grinder (Thomas Scientific, ACT-AG3080) in RIPA buffer (Cell Signaling, 9806S) supplemented with Halt Protease and Phosphatase Inhibitor Cocktail (ThermoFisher, 78444). Protein lysates were cleared by centrifugation and quantification was performed using Pierce BCA Protein Assay Kit (ThermoFisher, 23227). For Western blot of histones, acid histone extraction was performed by incubating nuclei in 0.4 N H_2_SO_4_ overnight and precipitating the solubilized histones by adding trichloroacetic acid to a final concentration of 33%^86^. Western blotting was performed by loading 10 μg protein western mix in 4–20% Mini-PROTEAN TGX Precast Protein Gels (BioRad, 4561096). Protein was transferred onto nitrocellulose membranes (GE Healthcare Life Sciences, 10600002), blocked in 5% NFDM in TBS with 0.1% Tween-20 (Millipore Sigma, 655206), and incubated at 4 °C overnight in primary antibody. The next morning, membranes were incubated in 1:2000 anti-rabbit IgG HRP-linked (Cell Signaling, 7074) or 1:2000 anti-mouse IgG HRP-linked (Cell Signaling, 7076) and imaged on a LI-COR Odyssey Fc. The following antibodies were used for western blotting: 1:500 anti-USP22 (Novus Biologicals, NBP1-49644), 1:1000 anti-NKX-2 (Proteintech, 13013-1-AP), 1:1000 anti-SEMA6A (ThermoFisher, PA5-81009), 1:500 anti-ASCL1 (BD Biosciences, 556604), 1:1000 anti-INSM1 (Santa Cruz, sc-271408), 1:1000 anti-phospho-RAD50 S635 (Cell Signaling, 14223), 1:1000 anti-RAD50 (Cell Signaling, 3427), 1:1000 anti-phospho-MRE11 S676 (Cell Signaling, 4859), 1:1000 anti-MRE11 (Cell Signaling, 4847), 1:1000 anti-phospho-Chk1 S345 (Cell Signaling, 2348), 1:1000 anti-Chk1 (Cell Signaling, 2360), 1:1000 anti-ubiquityl-histone H2A K119 (Cell Signaling, 8240), 1:1000 anti-histone H2A (Cell Signaling, 12349), 1:1000 anti-histone H3 (Cell Signaling, 4499), 1:1000 anti-HSP90 (Santa Cruz, sc-13119), 1:1000 anti-GAPDH (Santa Cruz, sc-32233), 1:1000 anti-alpha-Tubulin (Cell Signaling, 3873). Uncropped and unprocessed blots are provided as a Source Data File.

### Immunohistochemistry (IHC)

PDX tumors were fixed in 10% neutral-buffered formalin for 72 h and sectioned at 4 μM thickness into paraffin blocks and slides. Slides were deparaffinized in xylene and tissue sections were hydrated in an ethanol-water series. Antigen retrieval was performed in pre-heated citrate buffer (10 mM citric acid, 0.05% Tween-20, pH 6.0), and tissue sections were blocked first in 3.5% hydrogen peroxide and 10% methanol and then in 5% goat serum. Tissue sections incubated in primary antibody overnight at 4 °C in a humidified chamber. The next morning, tissue sections were incubated in biotinylated goat anti-rabbit IgG secondary antibody (Vector Laboratories, BP-9100-50) and then in VectaStain ABC kit (Vector Laboratories, PK-4000) reagents. Tissue sections were developed using DAB Substrate Kit Peroxidase (Vector Laboratories, SK-4100) and subjected to dehydration, clearing, and mounting. At least two images were captured per tissue section using a Nikon Ni-UI microscope and quantified manually at 400X magnification using ImageJ (Fiji). H-scoring was quantified manually using ImageJ. Each data point represents an independent tumor and is an average of two images per tumor. The following antibodies were used for IHC: 1:200 anti-phospho-histone H3 S10 (Millipore, 06-570), 1:100 anti-cleaved caspase-3 N175 (Cell Signaling, 9661), 1:200 anti-ASCL1 (Cell Signaling, 10585), 1:400 anti-NEUROD1 (Cell Signaling, 59418), 1:100 anti-INSM1 (Santa Cruz, sc-271408), 1:100 anti-SYP (Cell Signaling, CST-36406), 1:500 CHGA (Novus Biologicals, NB120-15160), and 1:100 anti-phospho-histone H2A.X S139 (Cell Signaling, 9718).

### Immunofluorescence

PDX tumors were dissociated into single-cell suspensions and incubated on 0.01% poly-L-lysine coated coverslips for 4 h at 37 °C following 48 h drug treatments in vivo or in vitro (*n* = 3 tumors per treatment condition). Adhered cells were fixed in 4% paraformaldehyde for 10 min, permeabilized in 0.2% Triton X-100 for 10 min, and blocked in 1% BSA with 22.52 mg/mL glycine for 30 min. Cells were incubated in 1:200 anti-phospho-histone H2A.X S139 (Cell Signaling, 80312) overnight at 4 °C. Cells were washed and incubated in 1:1000 anti-mouse AlexaFluor 647 (ThermoFisher, A-31571) for 1 h at room temperature. Cells were washed and counterstained in 1μg/mL DAPI (Invitrogen, 62248) for 5 min. Cells were washed a final time, mounted onto slides with Fluoromount-G (Invitrogen, 00-4958-02), and sealed with Prolong Coverslip Sealant (Invitrogen, P56128). Slides were imaged using a Andor Spinning Disk Dragonfly confocal microscope at ×630 magnification under oil immersion. At least 5-10 images (single z-plane images) were captured per slide depending on cell density. Images were analyzed using automated Image J software to quantify γH2AX cell MFI, γH2AX puncta count, and γH2AX MFI within puncta on a per basis in nuclei (DAPI). γH2AX raw signal was pseudocolored to magenta for visualization.

### Seahorse assay

Following saline or cis-eto treatment for 48 h, FHSC14 PDX tumors were dissociated into single-cell suspensions and plated on a Seahorse XF96 cell culture microplate (Agilent, 101085-004) coated with 0.01% poly-L-lysine at 2×10^5^ viable cells per 100 μL density for 4 h at 37 °C. Cells were washed, resuspended in Seahorse media [base DMEM media (Millipore, D5030) supplemented with 1 mM sodium pyruvate. 2 mM L-glutamine, and 5 mM HEPES, pH 7.4] and incubated at 37 °C in a non-CO_2_ incubator for 1 h prior to start of assay. Glycolytic stress test was performed by measuring extracellular acidification rates (ECAR) using a Seahorse XF96 Analyzer prior to and after sequential addition of glucose (10 mM final), oligomycin (2 μM final), and 2-DG (50 mM final). BCA assay (ThermoFisher, 23227) was used to standardize ECAR measurements to total protein content per well. The following equations were used to calculate glycolytic metrics as described by Report Generator User Guide - Agilent Seahorse XF Glycolytic Stress Test (Agilent, 103020-100) manufacturer’s protocol: Glycolysis = (ECAR_max_ before oligomycin injection)—(ECAR_min_ before glucose injection); Glycolytic Capacity = (ECAR_max_ after oligomycin injection)—(ECAR_min_ before glucose injection); Glycolytic Reserve = (Glycolytic Capacity)—(Glycolysis). The data shown are the average of 3 independent tumors (biological replicates) per condition plated with technical replicates.

### CRISPR knockout screening in vivo

#### Tumor processing, library transduction, and drug treatment

The focused SCLC library^25^ used in this study contained guide RNAs targeting 400 genes, with 6 gRNAs/gene cloned into lentiCRISPRv2 (Addgene, 52961), that were either altered/mutated in SCLC studies, implicated in SCLC chemoresistance, or hits in a previous pilot whole genome screen, and also included 120 non-targeting gRNAs. PDX tumors were dissociated into single-cell suspensions and transduced with concentrated lentivirus containing a custom focused 2520 sgRNA CRISPR knockout library or the Human CRISPR Knockout Pooled Library (GeCKO v2)^28^ in the presence of 8 μg/mL polybrene. Eighteen hours after transduction, a subset of cells were cultured for 48 more hours and saved as an initial timepoint (P0) comparator while the rest were washed and injected subcutaneously in both flanks of NSG mice at 1 × 10^6^ cells per flank. When tumors reached 300–350 mm^3^, mice were assigned to SAL or CIS-ETO treatment groups, where SAL tumors were immediately harvested, and CIS-ETO tumors were treated with 2 weekly cycles of cis-eto for the NCI109B PDX model until tumor ablation or 3 weekly cycles of cis-eto for FHSC14 and harvested upon regrowth to initial pre-treatment size. Harvested tumors were snap-frozen using dry ice and stored at −80 °C.

#### CRISPRko screen library preparation and sequencing

Genomic DNA containing the focused SCLC library was isolated^87^ from snap-frozen PDX tumors. Eight μg gDNA per tumor (8 × 1 μg reactions) underwent 16 PCR cycles using PLC-Seq-F (GAGGGCCTATTTCCCATGATTCCTTCA) and PLC-Seq-R (AACTTCTCGGGGACTGTGG) primers to amplify the focused SCLC gRNA library sequences to preserve library complexity. 10 μL of this first PCR product underwent a second set of 16 PCR cycles to add Truseq Single Index Library Illumina sequencing adaptors and unique barcodes to identify individual samples. The 230 base pair final PCR product was gel purified. CRISPR libraries prepared from the Focused SCLC screen samples or pilot whole genome^88^ screen samples were quantified, pooled, and sequenced on an Illumina HiSeq2500 high throughput flow cell at 11.5 pM with 57 base pair single-end reads.

#### CRISPRko screen analyses

Differential abundance analysis between treatment groups and the P0 group in pilot and focused CRISPR knockout screens was performed using MAGeCK^29^ version 0.5.7 (pilot genome scale screen) and version 0.5.9 (focused library screen). Samples underwent pooling to increase the number of gRNAs present and overall gRNA coverage per sample. In the pilot genome scale screen, DNA from 11 cis-eto- and 9 saline-treated tumors were pooled into a single DNA pool per group prior to library preparation and sequencing. In the focused library screen, individual tumors underwent computational pooling by randomly assigned tumors to pools and tumors for each computational pool were treated as technical replicates. gRNA counts were median normalized after pooling. Gene-level robust rank aggregation (RRA) scores were generated by MAGeCK. Gene-level and sgRNA-level p-values were corrected using Benjamini-Hochberg FDR procedure.

### RNA-seq

RNA was isolated from snap-frozen PDX tumors by mechanical dissociation and homogenization in TRIzol reagent (ThermoFisher, 15596026) and mRNA was enriched using the NEBNext Poly (A) mRNA Magnetic Isolation Module (New England Biolabs, E7490) kit. RNA libraries were prepared using the NEBNext Ultra II RNA Library Prep Kit for Illumina (New England BioLabs, E7770) and sequenced on an Illumina NextSeq P3 with 50 base pair single-end reads. Reads were aligned to human genome assembly hg38 and GENCODE gene annotation v38 as well as mouse genome assembly mm10 and GENCODE gene annotation vM23 using STAR v2.7.7a^89^ with 2-pass mapping. Reads of mouse origin were removed using R package XenofilteR^90^. Gene expression quantification was performed using FeatureCounts in Subread 2.0.0 and genes with low expression were excluded using edgeR function filterByExpr with min.count = 10 and min.total.count = 15. Following TMM normalization^91^, differentially expressed genes between sample groups were identified using Bioconductor package edgeR 3.34.1^92^ and were subjected to significance testing using GLM LRT method and Benjamini-Hochberg p-value correction. Up- or downregulated protein-coding genes with FDR < 0.05 were queried for pathway enrichment analysis using Enrichr^47,48^ (see Supplementary Data 9-12 for all significantly enriched or depleted pathways). Gene Set Enrichment Analysis (GSEA)^54^ was performed using version 4.3.3. The ASCL1 Targets gene set comprised a list of 141 ASCL1 target genes that were shared between mouse and human chromatin occupancy analyses^61^.

### CUT&Tag

Following saline or cis-eto treatments for 48 h, FHSC14 PDX tumors were dissociated and underwent FACS sorting to enrich viable cells. 50,000 cells per reaction were processed for CUT&Tag^67^ and sequenced on an Illumina NextSeq P3 with 50/50 base pair paired-end reads. The following antibodies were used for CUT&Tag: 1:100 ubiquityl-histone H2A K119 (Cell Signaling, 8240), 1:100 tri-methyl-histone H3 K27 (Cell Signaling, 9733), 1:100 normal Rabbit IgG (Cell Signaling, 2729). The nf-core cutandrun pipeline^93^ was followed for quality trimming using TrimGalore and alignment to GRCh38 reference genome using Bowtie2. MACS2^94,95^ was used to call broad peaks with and without IgG controls and an intersection of the two was considered the final peak set for each sample. For quality control, a combination of Samtools^96^ and the bedtools suite^97^ was used to calculate fragment length and number of reads in peaks. Peak annotation generated using Homer^98^ and Gencode v38 annotations. Read counts for each peak were generated using Subread featureCounts^99^, tabulated and passed to Bioconductor edgeR for differential peaks (FDR < 0.05) expressed between groups of interest (Supplementary Data 15). Heatmaps illustrating significantly up and down-regulated peaks were generated using deepTools^100^. Peak gene tracks for were viewed using Integrative Genomics Viewer (IGV) version 2.18.4^101^.

### Global Proteomics and phospho-proteomics mass spectrometry and data analysis

#### Protein lysate preparation

Frozen tumors (mean mass = 117 mg, range = 78–135 mg) were cryo-pulverized with a Covaris cryoPREP CP02 Impactor, and protein was solubilized in 1 mL urea lysis buffer (6 M urea, 25 mM Tris, pH 8.0, 1 mM EDTA, 1 mM EGTA, 1% Sigma Phosphatase Cocktail 2, 1% Sigma Phosphatase Cocktail 3, and 1% Sigma Protease Inhibitor Cocktail). Samples were vortexed at maximum speed for 15 s, and lysates were transferred to 1.7 mL screw-top microfuge tubes. Samples were subjected to 5 min of sonication using Qsonica Q800R3 sonicator. Samples were then cleared by centrifugation (20,000 RCR, 10 min at 4 °C) and transferred to cryovials (NUNC, Cat. #363401) for storage in the vapor phase in an LN2 tank. Protein concentrations of the clarified lysates were measured by Micro BCA assay (ThermoFisher Scientific, Cat. #23235).

#### Protein digestion

Lysates were diluted to 2.0 mg/mL using lysis buffer and 125 μL of lysate (250 μg of protein) was reduced with the addition of 8 μL of 500 mM TCEP (ThermoFisher) for 30 min at 37 °C with shaking, followed by alkylation with the addition of 18 μL of 500 mM iodoacetamide (Sigma) in the dark at room temperature for 30 min. Lysates were then diluted with 0.85 mL 200 mM Tris (pH 8.0). Lys-C (Wako) was dissolved in 25 mM Tris (pH 8.0) at 400 μg/mL and added to lysates at 1:50 (enzyme:protein) ratio by mass and incubated for 2 h at 37 °C with shaking. Trypsin was then added at a 1:100 trypsin:protein ratio and incubated overnight at 37 °C with shaking. After 16 h, the reaction was quenched with formic acid to a final concentration 1% by volume. Samples were desalted using Oasis HLB 96-well plates (Waters) and a positive pressure manifold (Waters). The plate wells were washed with 3 × 400 μL of 50% MeCN/0.1% FA and then equilibrated with 4 × 400 μL of 0.1% FA. The digests were applied to the wells, then washed with 4 × 400 μL 0.1% FA before being eluted drop by drop with 3 × 400 μL of 50% MeCN/0.1% FA. The eluates were lyophilized, followed by storage at −80 °C until use.

#### Isobaric labeling

Individual trypsin digestion samples were labeled with the TMTpro™ 16plex isobaric label reagent set (TMT; ThermoFisher Scientific, Cat. #A44520, Lot #YA357799). Desalted peptides were resuspended in 500 μL of 100 mM HEPES (pH 8.5). TMT reagents were resuspended in 200 μL MeCN and 100 μL was transferred to the peptide sample. Samples were incubated at room temperature for 1 h with mixing. Labeling reactions were quenched by the addition of 50 μL of hydroxylamine (Sigma, Cat. # 438227) diluted to 5% in H2O and incubated for 15 min at room temperature with mixing. The independent labeling reactions were then pooled together and lyophilized. The labeled peptides were desalted as above and then lyophilized and stored at −80 °C.

#### Basic (high pH) reverse phase (RP) liquid chromatography

The labeled, pooled, and desalted tryptic digests were fractionated by high-pH reverse phase (RP) liquid chromatography on an LC system consisting of an Agilent 1200 HPLC (Agilent, Santa Clara, CA) with mobile phases of 5 mM NH_4_HCO_3_, pH 10 (A) and 5 mM NH_4_HCO_3_ in 90% MeCN, pH 10 (B). The peptides were separated by a 4.6 mm × 250 mm Zorbax Extend- C18, 3.5 μm, column (Agilent Cat. #770953-902) over 96 min at a flow rate of 1.0 mL/min by the following timetable: hold 0% B for 9 min, gradient from 0 to 10% B for 4 min, 10 to 28.5% B for 50 min, 28.5–34% B for 5.5 min, 34 to 60% B for 13 min, hold at 60% B for 8.5 min, 60–0% B for 1 min, re-equilibrate at 0% B for 5 min. 1-min fractions were collected from 0 to 96 min by the shortest path by row in a 1 mL deep well plate (Thermo Cat. #95040450). The high pH RP fractions were concatenated into 24 samples by every other plate column (e.g., sample 1 contained fractions from wells A1, C1, E1, etc.). 95% of every 12th fraction of the 24 samples was combined (1,13; 2,14;…) to generate 12 samples, which were dried down and stored at −80 °C prior to phosphopeptide enrichment. The remaining 5% was dried down, re-suspended in 0.1% FA, 3% MeCN, and frozen at −80 °C until analysis.

#### Immobilized metal affinity chromatography (IMAC)

For discovery LC-MS/MS analysis, fractionated samples were enriched for phosphopeptides by immobilized metal affinity chromatography (IMAC). Enrichment was performed using Ni-NTA-agarose beads (Qiagen, Cat. #36113) stripped with EDTA and incubated in a 10 mM FeCl3 solution to prepare Fe3 + -NTA-agarose beads. Peptide samples were reconstituted in 200 μL of 0.1% TFA in 80% MeCN and incubated for 30 min with 100 µL of the 5% bead suspension, mixing at 1400 rpm at room temperature. After incubation, the beads were washed 3 times with 300 µL of 0.1% TFA in 80% MeCN. Phosphorylated peptides were eluted from the beads using 200 μL of 70% MeCN, 1% ammonium hydroxide for 1 min with agitation at room temperature (do not exceed 5 min). Samples were transferred into a fresh tube containing 60 μL of 10% FA, dried down and re-suspended in 0.1% FA, 3% MeCN. The samples were frozen at −80 °C until analysis.

#### Nano-liquid chromatography-tandem mass spectrometry

Fractionated samples both global proteome samples and phosphopeptide-enriched samples were analyzed by LC-MS/MS on an Easy-nLC 1000 (Thermo Scientific) coupled to an Orbitrap™ Eclipse™ Tribrid™ mass spectrometer (Thermo Scientific) operated in positive ion mode with a FAIMS Pro™ Interface. The LC system consisted of a fused-silica nanospray needle (PicoTip™ emitter, 50 µm ID x 27 cm, New Objective) packed in-house with ReproSil-Pur 120 C18-AQ (Dr. Maisch) and a trap (IntegraFrit™ Capillary, 100 µm ID x 2 cm, New Objective) packed in-house with Magic C18-AQ with mobile phases of 0.1% FA in water (A) and 0.1% FA in 80% MeCN (B). The peptide sample was diluted in 20 µL of 0.1% FA, 2% MeCN and 8.5 µL was loaded onto the column and separated over 150 min at a flow rate of 300 nL/min with a gradient from 4 to 9% B for 2 min, 9 to 25% B for 80 min, 25 to 44% B for 60 min, 44–63% B for 8 min, hold 63% B for 1 min, 63 to 90% B for 11 min, hold 90% B for 1 min. A spray voltage of 2300 V was applied to the nanospray tip. FAIMS MS/MS analysis occurred over a 3 s cycle time consisting of 1 full scan MS from 400-1600 m/z at resolution 120,000 followed by data dependent MS/MS scans cycling through FAIMS CV of −40, −60 and −80 using HCD activation at resolution 50,000 with 38% normalized collision energy of the most abundant ions. Selected ions were dynamically excluded for 60 s after a repeat count of 1.

#### Shotgun mass spectrometry data analysis

Raw MS/MS spectra from the analysis were searched twice using MSFragger v3.8^102^ first against the reviewed Human Universal Protein Resource (UniProt) sequence database downloaded on 11/1/2023 and then against a database combining both Human and Mouse UniProt sequence databases (both downloaded on 11/1/2023). Both databases were appended with reverse sequences and common contaminants. MS and MS/MS mass calibration, MS/MS spectral deisotoping, and parameter optimization were enabled^103^. For the analysis of whole proteome data, cysteine carbamidomethylation ( + 57.0215), TMT labeling ( + 304.20715) of lysine and the peptide N-terminal were specified as fixed modifications and methionine oxidation ( + 15.9949) was specified as a variable modification. For phosphopeptide enriched data, phosphorylation ( + 79.9663) of serine, threonine, and tyrosine residues also included were specified as a variable modification. The search was restricted to tryptic peptides, allowing up to two missed cleavage sites. The post-processing of the search results was done using the Philosopher toolkit version v5.0.0. MSFragger output files (in pepXML format) were processed using PeptideProphet^104^ (with the high–mass accuracy binning and semi-parametric mixture modeling options) to compute the posterior probability of correct identification for each peptide to spectrum match (PSM). In the phosphopeptide-enriched dataset, PeptideProphet files were additionally processed using PTMProphet^105^ to localize the phosphorylation sites. The resulting pepXML files from PeptideProphet (or PTMProphet) were then processed together to assemble peptides into proteins (protein inference) and to create a combined file (in protXML format) of high confidence protein groups. The combined protXML file were further processed using the Philosopher filter command as follows. Each peptide was assigned either as a unique peptide to a particular protein group or set as a razor peptide to a single protein group with the most peptide evidence. The protein groups assembled by ProteinProphet^106^ were filtered to 1% protein-level False Discovery Rate (FDR) using the best peptide approach (allowing both unique and razor peptides) and applying the picked FDR target-decoy strategy. The PSM lists were filtered using a sequential FDR strategy, retaining only those PSMs with PeptideProphet probability of 0.9 or higher (which in these data corresponded to less than 1% PSM-level FDR) and mapped to proteins that also passed the global 1% protein-level FDR filter. For each PSM that passed these filters, the corresponding precursor ion MS1 intensity was extracted using the Philosopher label-free quantification module, using 10 ppm mass tolerance and 0.4 min retention time window for extracted ion chromatogram peak tracing. For all PSMs corresponding to a TMT-labeled peptide, 16 TMT reporter ion intensities were extracted from the MS/MS scans (using 0.002 Da window). The precursor ion purity scores were calculated using the intensity of the sequenced precursor ion and that of other interfering ions observed in MS1 data (within a 0.7 Da isolation window). All supporting information for each PSM, including the accession numbers and names of the protein/gene selected based on the protein inference approach with razor peptide assignment and quantification information (MS1 precursor-ion intensity and the TMT reporter ion intensities), was summarized in the output PSM.tsv files. To generate summary reports on different levels (gene, peptide, and protein for global and phosphopeptide enriched data; additional modification site report for phosphopeptide data), all PSM.tsv files were processed together using TMT-Integrator^107^ using default parameters described previously^108^. In generating the site-level reports (phosphopeptide-enriched data), sites with PTMProphet computed localization probability equal to or greater than 0.75 were considered as confidently localized.

#### Global proteomics and phospho-proteomics analyses

The proteins and phospho-sites that were quantified in at least 50% of samples were included in the analysis for each PDX model. The tandem mass tags (TMT) values were median-normalized and log2-transformed before analysis. Missing values were imputed using the DreamAI algorithm, which is an ensemble of six commonly used imputation methods that was optimized specifically for proteomics datasets^109^. Log2-transformed relative abundances were compared between treatment groups (cis-eto vs saline) and genotype (sgCtrl vs sgUSP22 for FHSC14 and NCI109B tumors and Empty-PLVX vs USP22-PLVX for JHU-LX33 tumors) using linear regression models with treatment group, genotype, and treatment x genotype interaction terms. Multiple testing was accounted for using the Benjamini-Hochberg FDR procedure.

#### PTM signature enrichment analysis

Post-translational modification signature enrichment analysis (PTM-SEA) was conducted using ssGSEA2.0 as implemented in the ssGSEA2 R package^69^. The PTMsigDB version 2.0.0 database^69^ was used to map phospho-sites to kinases. The relative abundances of each phosphorylated protein were compared between cis-eto and saline groups as described in the Phospho-proteomics Analysis section, the results of which were used for PTM-SEA calculations. Specifically, the ranking metric for each phospho-site was calculated as −10log_10_(p-value) multiplied by the sign of the log2-fold change estimate. Kinases in the PTMsigDB under the KINASE-PSP category with at least 5 phospho-sites that overlapped with the phospho-proteomics results were included in the analysis. Kinase-level enrichment was quantified using a normalized enrichment score (NES) and the corresponding *p* value was calculated using permutation tests (1000 permutations). Kinase-level *p* values were adjusted using the Benjamini-Hochberg FDR procedure. The specific parameters used for the ssGSEA2 package were: statistic = “area.under.RES”, correl.type = “rank”, sample.norm.type = “none”, weight = 0.75, nperm = 1000, and min.overlap = 5.

### Statistical analysis

All statistical tests and number of biological replicates per group are included in the figure legends or figure display items. Data are presented as mean ± standard error of measure (SEM). Statistical analyses were performed using GraphPad Prism software. Statistical tests were performed by unpaired two-sided student’s *t* test, 1-way ANOVA followed by Tukey’s multiple comparison test, or 2-way ANOVA followed by Tukey’s multiple comparison test where indicated. Unadjusted *t* test *p* values and adjusted ANOVA p-values are shown on figures when *p* < 0.05; NS non-significant.

### Reporting summary

Further information on research design is available in the Nature Portfolio Reporting Summary linked to this article.

## Supplementary information

Supplementary Information

Description of Additional Supplementary Files

Supplementary Data

Reporting Summary

Transparent Peer Review file

## Source data

Source Data

## Data Availability

The LC-MS/MS proteomics data are deposited to the ProteomeXchange Consortium^110^ via the PRIDE partner repository with the dataset identifier PXD048813. The RNA-seq and CUT&RUN data are available in dbGaP under the accession number phs004087.v2.p1 [https://dbgap.ncbi.nlm.nih.gov/beta/search/?OBJ=study&TERM=phs004087.v2.p1] under restricted access due to individual privacy concerns related to the patients from which the PDX models were derived. Faculty or senior scientists with laboratory oversight responsibilities can request access through dbGaP. The requests are reviewed by NCI’s Data Access Committee, with access permitted for one year, with option to renew annually. PDX models with genetic perturbation will be made available by contacting David MacPherson with completion of appropriate MTAs. The remaining data are available within the Article, Supplementary Information, Supplementary Data, or Source Data File. Source data are provided with this paper.
