# Supplementary material for: Loss of the USP22 deubiquitylase confers resistance to chemotherapy in small cell lung cancer
Source: Nat Commun. 2026 Jul 8;17:8402. doi: 10.1038/s41467-026-75117-2 (PMC13473660; doi:10.1038/s41467-026-75117-2)
Supplement: Supplementary file 1 [file 41467_2026_75117_MOESM1_ESM.pdf]

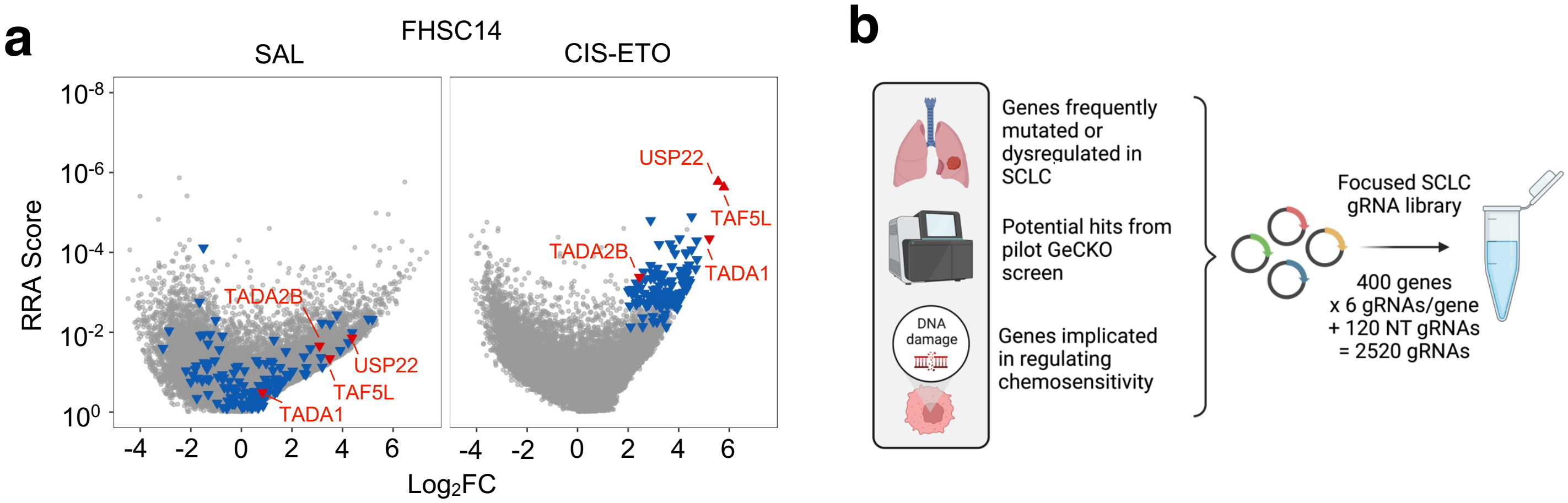

Supplementary Figure 1: Pilot genome-scale CRISPRko screen and design of the focused gRNA library, related to Figure 1

FHSC14 patient-derived xenograft (PDX) tumors were dissociated into single-cell suspensions and transduced with concentrated lentivirus containing the Human CRISPR Knockout Pooled Library (GeCKo v2) ex vivo. After 18 hours, a subset of cells were left in culture for 72 hours to serve as an initial timepoint (P0), while the rest were injected subcutaneously in the flanks of NSG mice at ~$1x10^6$ cells per flank. Once tumors reached 150 mm^3, tumors were treated with either saline or 3 weekly cycles of cis-eto. Saline tumors were collected once they reached 1000 mm^3 and cis-eto tumors were collected upon tumor return. In each treatment group, extracted gDNA from each tumor was pooled prior to sequencing at an equimolar ratio to generate a saline (n=11 pooled tumors) pool and a cis-eto (n=12 pooled tumors) pool. **(a)** Gene-level volcano plots of MAGeCK analyses results from the FHSC14 pilot genome-scale. The positive robust rank aggregation (RRA) score is shown for genes with $Log_2FC > 0$ and the negative RRA is shown for genes with $Log_2FC < 0$ (a smaller RRA score indicates stronger and more consistent enrichment or depletion of gRNAs for the same gene). Genes displayed in blue or red are 130 genes with $Log_2FC \geq 2$ selected amongst the top enriched genes in the CIS-ETO condition for inclusion in a focused sgRNA library. SAGA complex members amongst these 130 genes are displayed in red. FDR < 0.2 for genes with right-side up triangles, and FDR > 0.2 for genes with upside down triangles. **(b)** Schematic showing the source of genes that were selected for the focused SCLC gRNA library. Created in BioRender. Best, S. (2026) https://BioRender.com/22p9q6j.

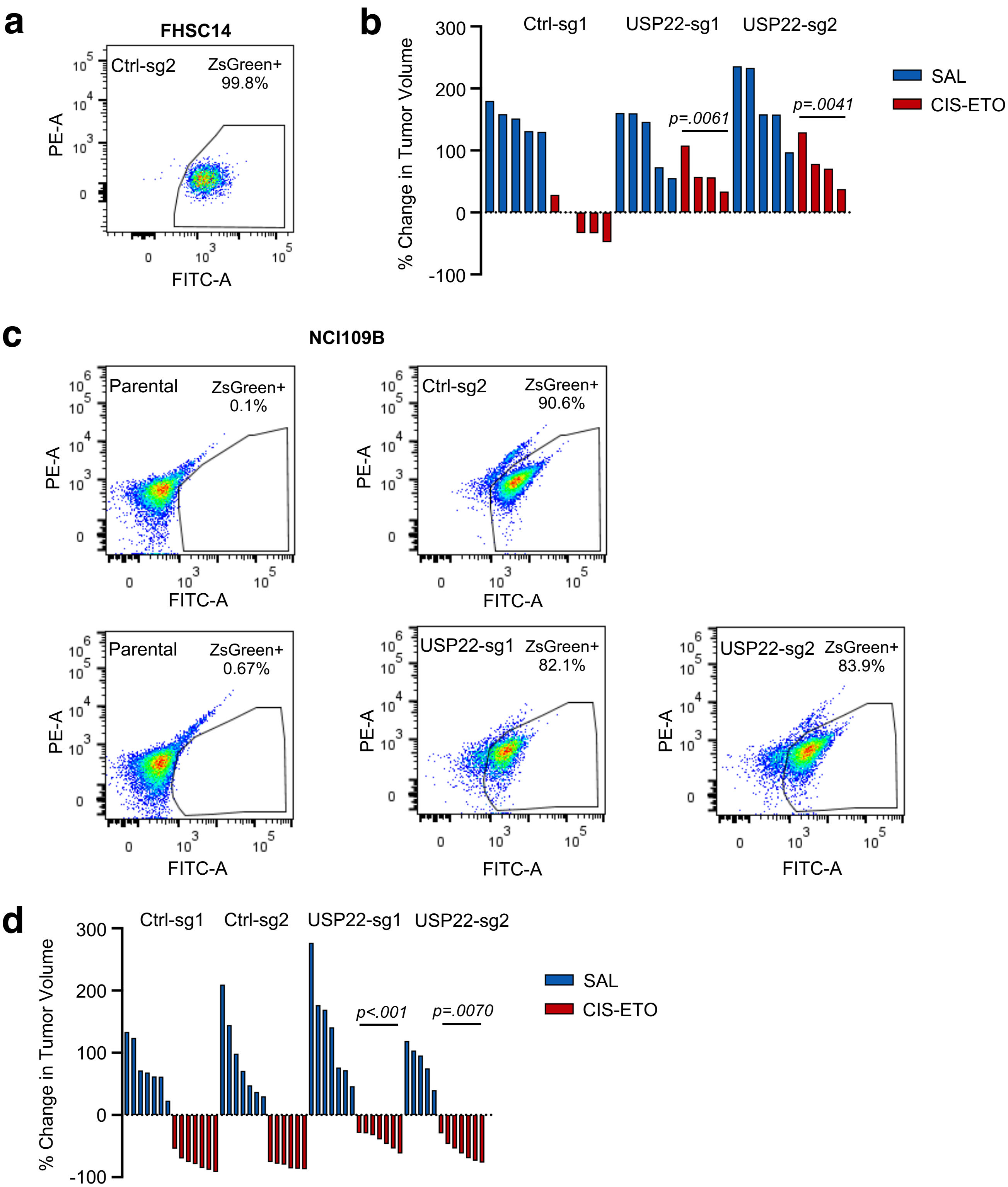

Supplementary Figure 2: Generation of isogenic SCLC PDX models with USP22 deletion and quantification of tumor response to chemotherapy treatment, related to Figure 2

(**a**) ZsGreen$^+$ purity of FHSC14 Ctrl-sg2 PDX models generated after 2 rounds of fluorescence-activated cell sorting (FACS). (**b**) FHSC14 Ctrl-sg1, USP22-sg1, USP22-sg2 PDX models were treated with saline or cis-eto over 10 days. %Change in Tumor Volumes from Day 0 to Day 10 are quantified. Data for mice bearing tumors that did not survive to Day 10 of treatment are omitted. (**c**) ZsGreen$^+$ purity of NCI109B PDX models generated after 2 rounds of FACS sorting. (**d**) NCI109B PDX models were treated with saline or cis-eto over 6 days. %Change in Tumor Volumes from Day 0 to Day 6 are quantified. Data for mice bearing tumors that did not survive to Day 6 of treatment are omitted. Statistical analyses in **b** comparing Ctrl-sg1 vs USP22-sg1 and Ctrl-sg1 vs USP22-sg2 CIS-ETO groups and in **d** comparing Ctrl-sg1 vs USP22-sg1 and Ctrl-sg2 vs USP22-sg2 CIS-ETO groups were performed by unpaired student's two-sided t-test. Unadjusted t-test p-values are shown on figures. Source Data is provided in the Source Data File.

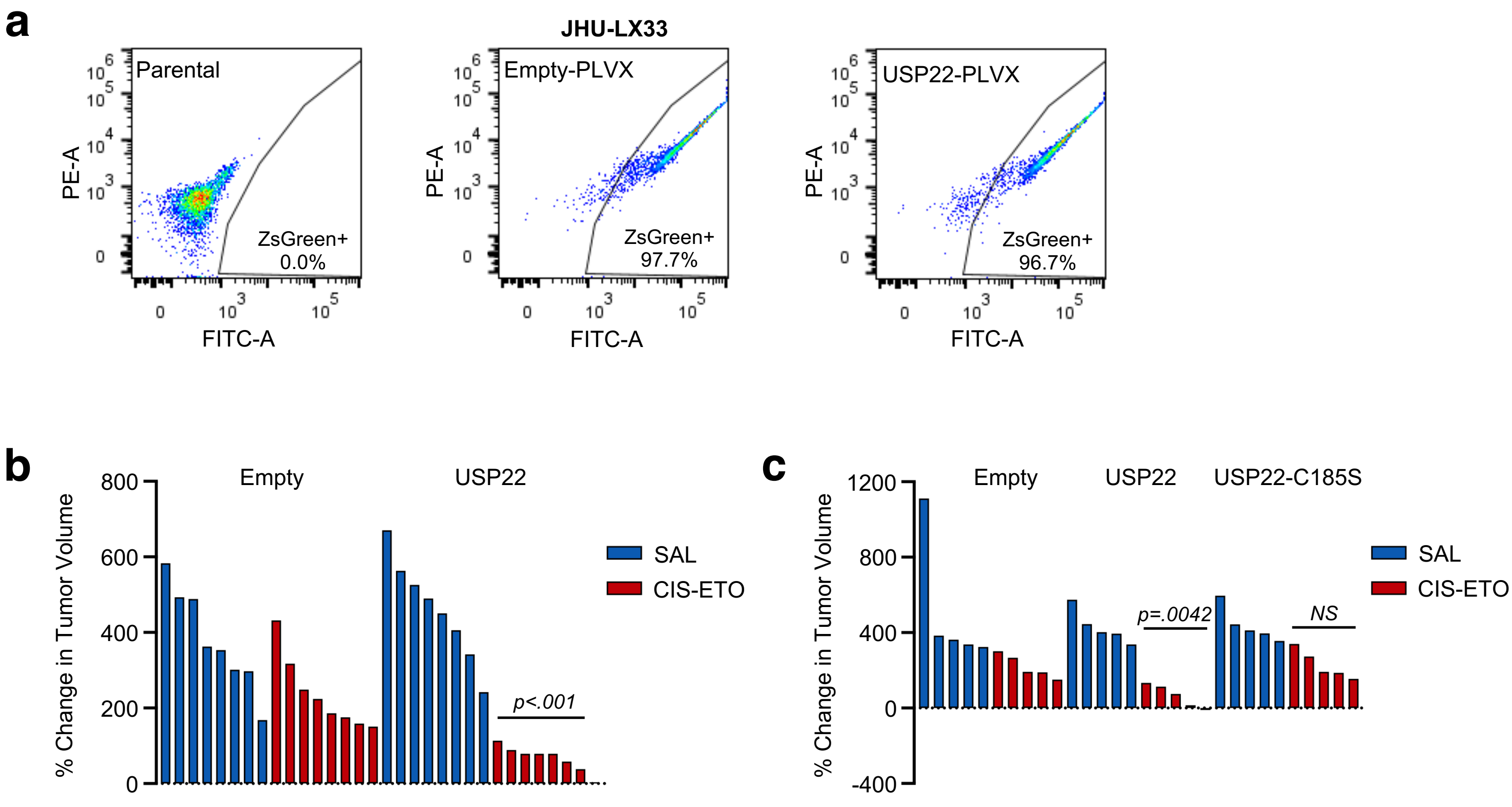

Supplementary Figure 3: Returning USP22 to a USP22-null SCLC PDX model JHU-LX33 and quantification of tumor response to chemotherapy treatment, related to Figure 3
(**a**) ZsGreen⁺ purity of JHU-LX33 Empty vector control vs USP22-overexpressing PDX models generated after 1 round of FACS sorting. (**b**) JHU-LX33 Empty-PLVX and USP22-PLVX models were treated with saline or cis-eto over 10 days. %Change in Tumor Volumes from Day 0 to Day 10 are quantified. Data for mice bearing tumors that did not survive to Day 10 of treatment are omitted. (**c**) JHU-LX33 Empty-PLVX, USP22-PLVX, and USP22-C185S-PLVX cell lines were injected into the flanks of NSG mice. Isogenic models were treated with saline or cis-eto over 10 days. %Change in Tumor Volumes from Day 0 to Day 10 are quantified. Data for mice bearing tumors that did not survive to Day 10 of treatment are omitted. Statistical analyses in **b** comparing Empty vs USP22 CIS-ETO groups and in **c** comparing Empty vs USP22 and Empty vs USP22-C185S CIS-ETO groups were performed by unpaired student's two-sided t-test. Unadjusted t-test p-values are shown on figures; NS=non-significant. Source Data is provided in the Source Data File.

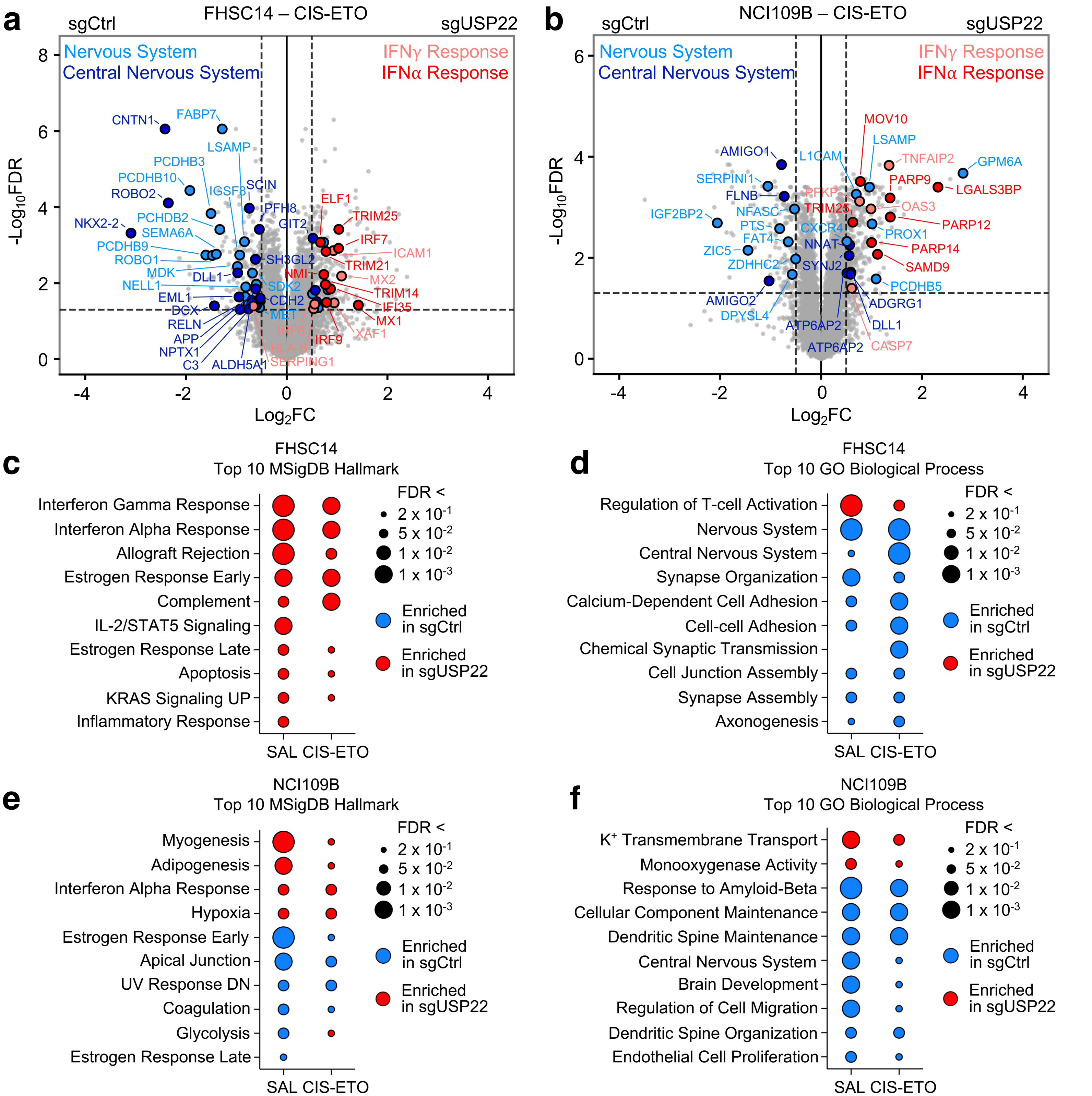

Supplementary Figure 4: Global proteomics and pathway analyses in PDX models with *USP22* deletion, related to Figure 4.

(**a-b**) Volcano plots of global proteomics data from 48 hour cis-eto treated FHSC14 USP22-sg1/2 vs Ctrl-sg1/2 tumors (n=2 tumors per sgRNA for a total of n=4 tumors per treatment group) (a) and NCI109B sgUSP22 vs sgCtrl tumors (n=3 tumors per treatment group) (b). Differentially expressed upregulated ($Log_2FC > 0.5$, $FDR < 0.05$) or downregulated ($Log_2FC < -0.5$, $FDR < 0.05$) proteins belonging to the indicated Gene Ontology Biological Process 2023 and MSigDB Hallmark 2020 pathways are labelled in blue and red, respectively. (**c-f**) Upregulated or downregulated proteins in FHSC14 (c-d) and NCI109B (e-f) sgUSP22 vs sgCtrl tumors treated for 48 hours with saline or cis-eto were independently queried for pathway enrichment using Enrichr. The top 10 significantly enriched/depleted pathways from MSigDB Hallmark and Gene Ontology Biological Process gene set databases for each PDX models with *USP22* deletion are shown. Blue circles indicate pathways enriched in sgCtrl and red circles indicate pathways enriched in sgUSP22 tumors. Point size scaled to FDR.

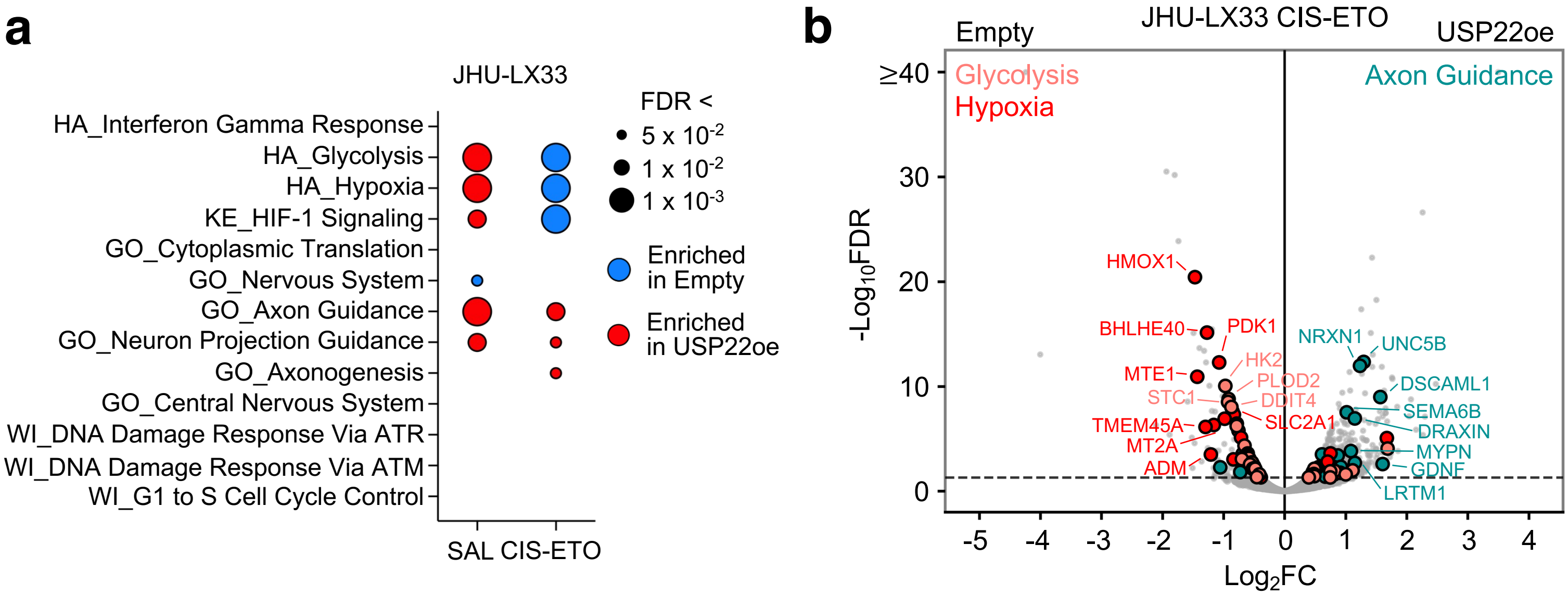

Supplementary Figure 5: RNA-seq analyses in JHU-LX33 model with USP22 add-back, related to Figure 5. (**a**) Differentially expressed upregulated or downregulated genes (FDR < 0.05) from RNA-seq data in JHU-LX33 USP22-PLVX vs Empty-PLVX models (n=5 tumors per treatment group) treated for 10 days with saline or cis-eto were independently queried for pathway enrichment using Enrichr. Pathways from Gene Ontology Biological Process 2023 (GO), MSigDB Hallmark 2020 (HA), KEGG 2021 Human (KE), WikiPathways 2024 Human (WI) gene set databases commonly enriched in or depleted in at least 2 out of 3 PDX models with respect to USP22 status are displayed (see Supplemental Tables 9-12 for all significantly enriched or depleted pathways). Point size scaled to FDR. (**b**) Volcano plot of RNA-seq differential gene expression analysis from 10-day cis-eto treated JHU-LX33 USP22-PLVX vs Empty-PLVX models. Genes belonging to the indicated MSigDB Hallmark 2020 and Gene Ontology Biological Process 2023 pathways are labelled in red and teal, respectively.

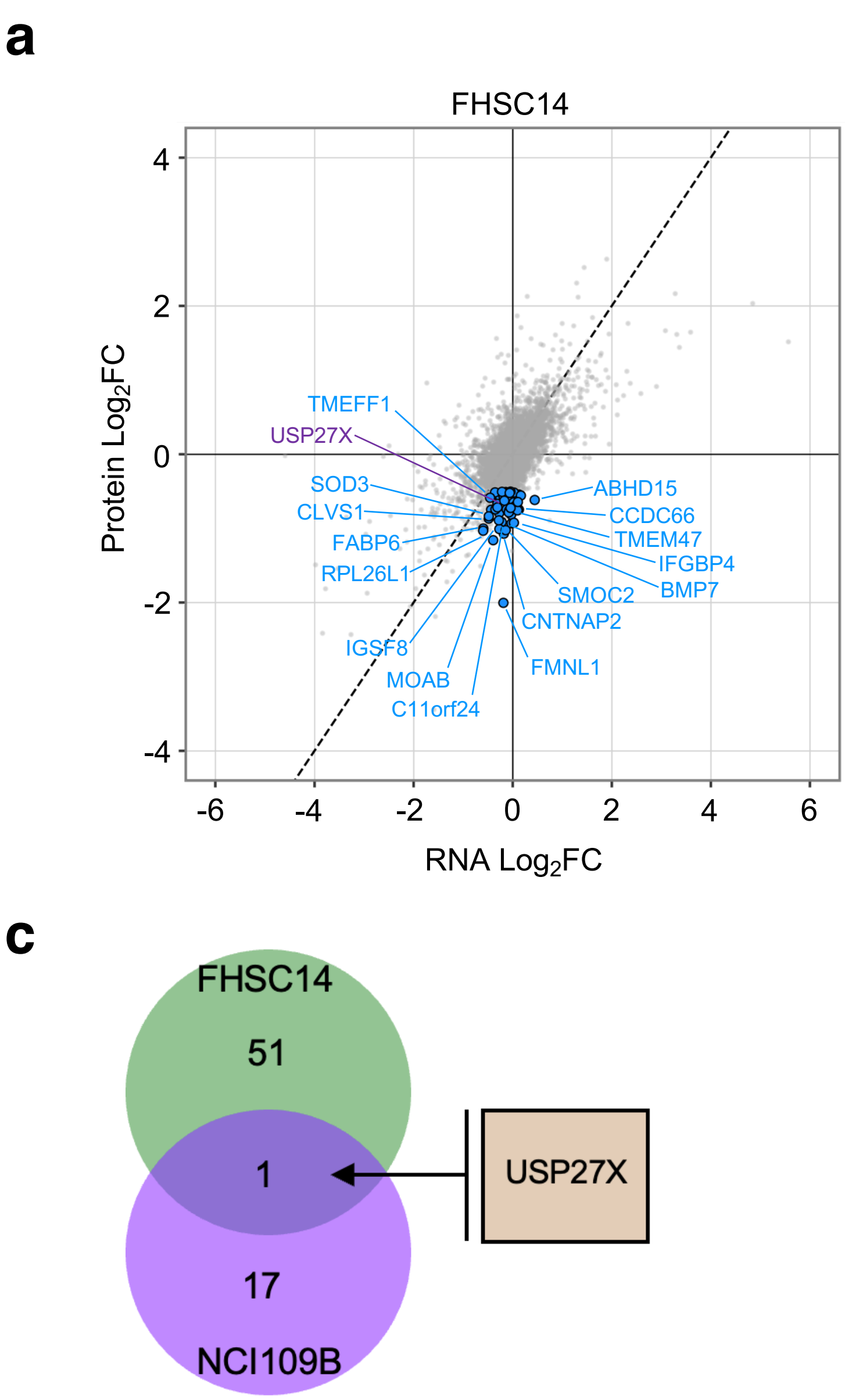

Supplementary Figure 6: Proteomics vs RNA-seq analyses in isogenic SCLC PDX models to identify candidate non-histone substrates of USP22, related to Figure 5.
(**a-b**) Global proteomics vs RNA-seq data from FHSC14 (a) and NCI109B (b) sgUSP22 vs sgCtrl tumors. Genes downregulated at the protein level ($Log_2FC < -0.5$, FDR < 0.05 in GLBL proteomics dataset) but not the RNA level (≠ $Log_2FC < -0.5$ with FDR < 0.05 in RNA-seq dataset) in sgUSP22 tumors are labelled (**c**) Overlap of genes downregulated at the protein level but not the RNA level in FHSC14 and NCI109B sgUSP22 models.

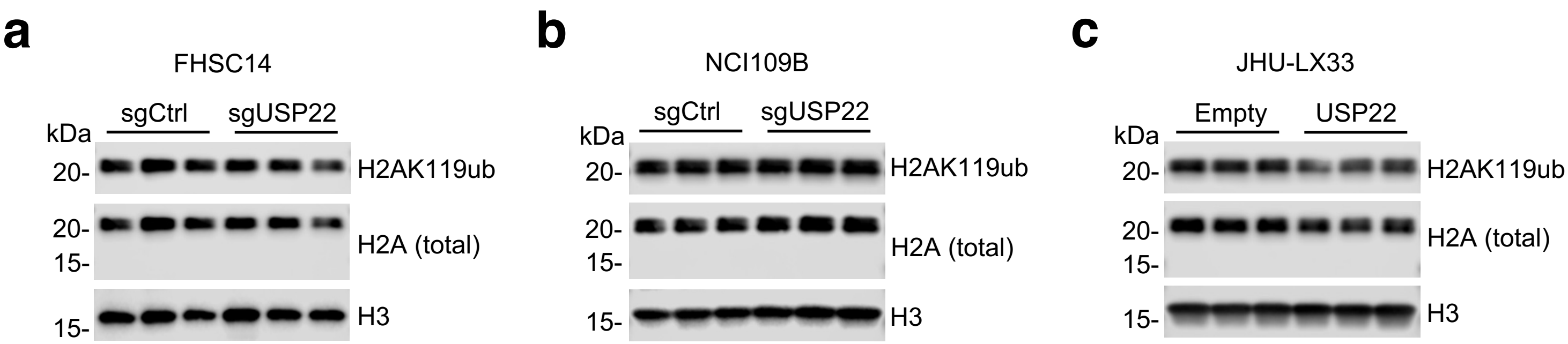

Supplementary Figure 7: Western blot analyses for global H2AK119 ubiquitylation in isogenic SCLC PDX models with *USP22* deletion or add-back, related to Figure 6.

(**a-c**) Western blots for H2AK119ub, H2A, and H3 (loading control) of acid-extracted histones from isogenic SCLC PDX models FHSC14 (a) and NCI109B (b) with *USP22* deletion and JHU-LX33 (c) with USP22 add-back compared to control models. Each lane in an independent tumor (n=3 tumors per group), with each blot representative from two independent experiments. Source Data is provided in the Source Data File.

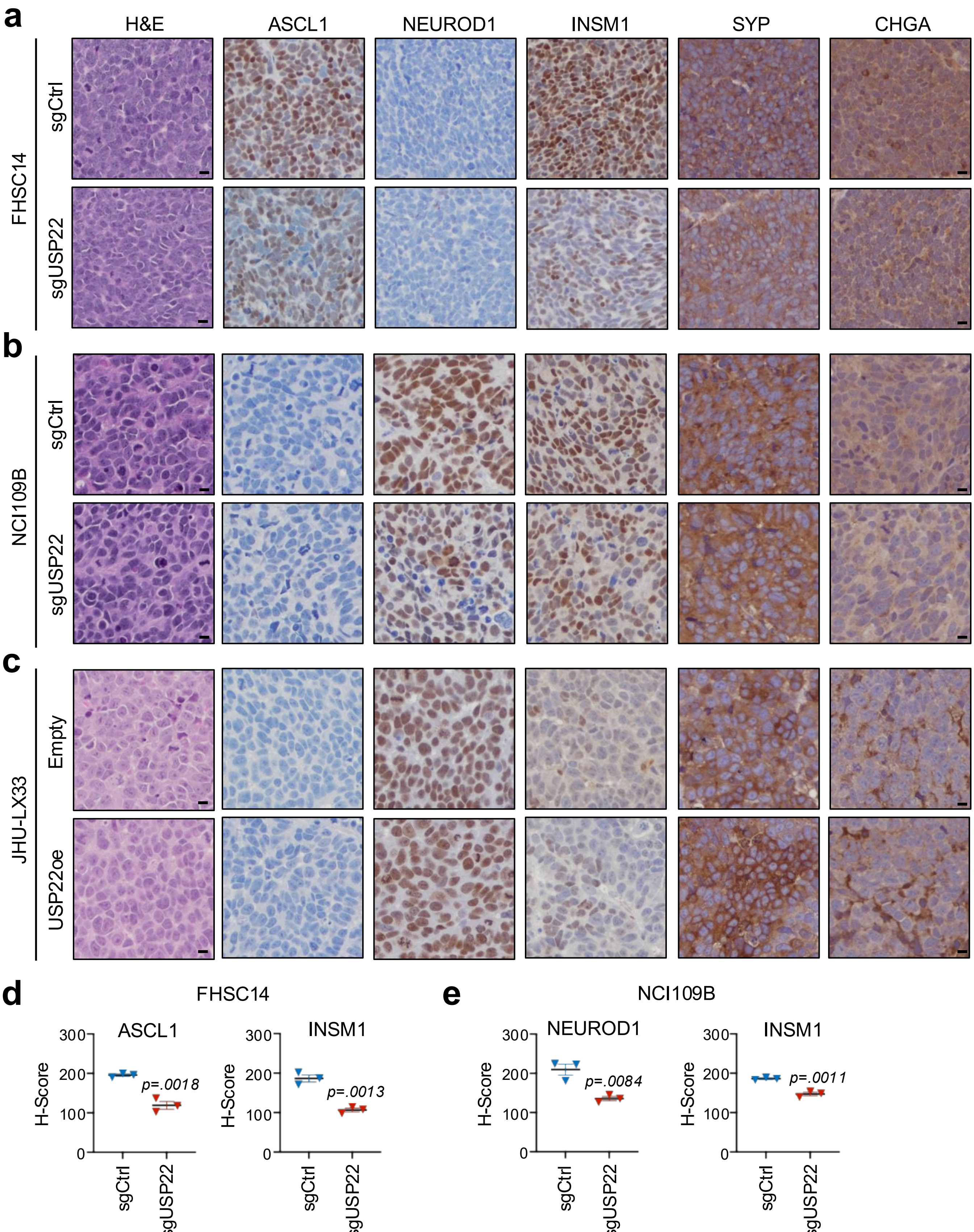

Supplementary Figure 8: Immunohistochemistry (IHC) analyses for neuroendocrine markers in isogenic SCLC PDX models with *USP22* deletion, related to Figure 6.

(**a-c**) H&E (left) and IHC (right) representative images for neuroendocrine markers in FHSC14 sgCtrl vs sgUSP22 (a), NCI109B sgCtrl vs sgUSP22 (b), and JHU-LX33 Empty vs USP22 PLVX (c), with each image representative from three independent experiments. Scale bar, 10 µm. (**d**) Quantification of IHC analyses for ASCL1 and INSM1 in FHSC14 sgUSP22 vs sgCtrl. Data are means ± SEM; n=3 tumors per condition. (**e**) Quantification of IHC analyses for NEUROD1 and INSM1 in NCI109B sgUSP22 vs sgCtrl. Data are means ± SEM, n=3 tumors per group. Statistical analyses in **d**, **e** were performed by unpaired student's two-sided t-test. Unadjusted t-test p-values are shown on figures. Source Data is provided in the Source Data File.

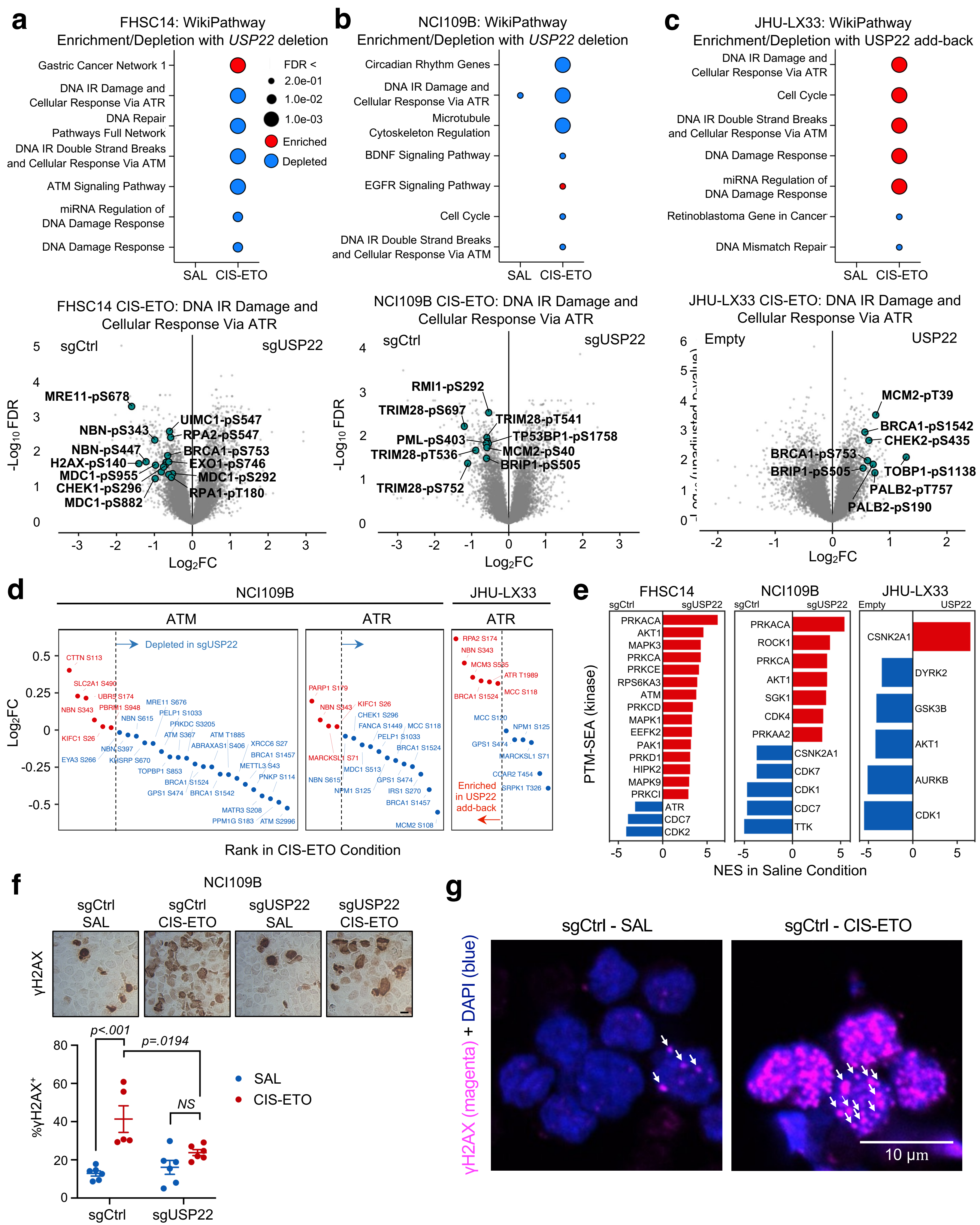

a
FHSC14: WikiPathway
Enrichment/Depletion with USP22 deletion
Gastric Cancer Network 1
DNA IR Damage and Cellular Response Via ATR
DNA Repair Pathways Full Network
DNA IR Double Strand Breaks and Cellular Response Via ATM
ATM Signaling Pathway
miRNA Regulation of DNA Damage Response
DNA Damage Response
SAL
CIS-ETO
FDR <
2.0e-01
1.0e-02
1.0e-03
Enriched
Depleted
b
NCI109B: WikiPathway
Enrichment/Depletion with USP22 deletion
Circadian Rhythm Genes
DNA IR Damage and Cellular Response Via ATR
Microtubule Cytoskeleton Regulation
BDNF Signaling Pathway
EGFR Signaling Pathway
Cell Cycle
DNA IR Double Strand Breaks and Cellular Response Via ATM
SAL
CIS-ETO
c
JHU-LX33: WikiPathway
Enrichment/Depletion with USP22 add-back
DNA IR Damage and Cellular Response Via ATR
Cell Cycle
DNA IR Double Strand Breaks and Cellular Response Via ATM
DNA Damage Response
miRNA Regulation of DNA Damage Response
Retinoblastoma Gene in Cancer
DNA Mismatch Repair
SAL
CIS-ETO
FHSC14 CIS-ETO: DNA IR Damage and Cellular Response Via ATR
sgCtrl
sgUSP22
MRE11-pS678
NBN-pS343
UIMC1-pS547
RPA2-pS547
NBN-pS447
BRCA1-pS753
H2AX-pS140
EXO1-pS746
MDC1-pS955
MDC1-pS292
CHEK1-pS296
RPA1-pT180
MDC1-pS882
-Log10 FDR
Log2FC
NCI109B CIS-ETO: DNA IR Damage and Cellular Response Via ATR
sgCtrl
sgUSP22
RMI1-pS292
TRIM28-pS697
TRIM28-pT541
PML-pS403
TP53BP1-pS1758
TRIM28-pT536
MCM2-pS40
BRIP1-pS505
TRIM28-pS752
-Log10 FDR
Log2FC
JHU-LX33 CIS-ETO: DNA IR Damage and Cellular Response Via ATR
Empty
USP22
MCM2-pT39
BRCA1-pS1542
CHEK2-pS435
BRCA1-pS753
BRIP1-pS505
TOBP1-pS1138
PALB2-pT757
PALB2-pS190
Log2FC
d
NCI109B
JHU-LX33
ATM
ATR
ATR
Depleted in sgUSP22
Enriched in USP22 add-back
Log2FC
Rank in CIS-ETO Condition
CTTN S113
SLC2A1 S490
UBR5 S174
NBN S343
PBRM1 S948
KIFC1 S26
EYA3 S266
MRE11 S676
PELP1 S1033
NBN S615
PRKDC S3205
ATM S367
ATM T1885
NBN S397
ABRAXAS1 S406
XRCC6 S27
KHSRP S670
BRCA1 S1457
METTL3 S43
TOPBP1 S853
PNKP S114
BRCA1 S1524
GPS1 S474
BRCA1 S1542
MATR3 S208
PPM1G S183
ATM S2996
PARP1 S179
NBN S343
KIFC1 S26
CHEK1 S296
FANCA S1449
MCC S118
PELP1 S1033
BRCA1 S1524
MARCKSL1 S71
MDC1 S513
GPS1 S474
NBN S615
NPM1 S125
IRS1 S270
BRCA1 S1457
MCM2 S108
RPA2 S174
NBN S343
MCM3 S535
ATR T1989
BRCA1 S1524
MCC S118
MCC S120
NPM1 S125
GPS1 S474
MARCKSL1 S71
CCAR2 T454
SRPK1 T326
e
FHSC14
sgCtrl
sgUSP22
PRKACA
AKT1
MAPK3
PRKCA
PRKCE
RPS6KA3
ATM
PRKCD
MAPK1
EEFK2
PAK1
PRKD1
HIPK2
MAPK9
PRKCI
ATR
CDC7
CDK2
NCI109B
sgCtrl
sgUSP22
PRKACA
ROCK1
PRKCA
AKT1
SGK1
CDK4
PRKAA2
CSNK2A1
CDK7
CDK1
CDC7
TTK
JHU-LX33
Empty
USP22
CSNK2A1
DYRK2
GSK3B
AKT1
AURKB
CDK1
PTM-SEA (kinase)
NES in Saline Condition
f
NCI109B
sgCtrl SAL
sgCtrl CIS-ETO
sgUSP22 SAL
sgUSP22 CIS-ETO
γH2AX
p<.001
p=.0194
NS
SAL
CIS-ETO
%γH2AX+
sgCtrl
sgUSP22
g
sgCtrl - SAL
sgCtrl - CIS-ETO
γH2AX (magenta) + DAPI (blue)
10 μm

Supplementary Figure 9: Phospho-proteomics, immunohistochemistry (IHC), and γH2AX immunofluorescence (IF) analyses for DNA damage signaling in SCLC PDX models with *USP22* deletion or USP22 add-back, related to Figure 7.

(**a**-**c**) Phospho-proteomic analysis of FHSC14 and NCI109B sgUSP22 vs sgCtrl PDX models, and JHU-LX33 USP22 vs Empty control vector treated with cis-eto or saline for 48 hours. Genes whose corresponding phospho-sites were enriched ($Log_2FC > 0.5$, $FDR < 0.05$) or depleted ($Log_2FC < -0.5$, $FDR < 0.05$) in FHSC14 USP22-sg1/2 vs Ctrl-sg1/2 (a), NCI109B sgUSP22 vs sgCtrl (b), or JHU-LX33 USP22 add-back vs Empty vector control (c) were queried for pathway enrichment using Enrichr. The Top 7 most significant pathways are displayed for each PDX model. For **a**-**c**, point size is scaled to FDR, and volcano plots show all significant phospho-sites (depleted in FHSC14 and NCI109B sgUSP22 vs sgCtrl, enriched in JHU-LX33 USP22 vs Empty vector control) whose respective genes were in the WikiPathway DNA IR Damage and Cellular Response Via ATR pathway. **(d)** PTM signature enrichment analysis (PTM-SEA) showing ATM and ATR kinase signatures for NCI109B and ATR kinase signature for JHU-LX33 in the CIS-ETO condition, with inference of decreased kinase activity upon USP22 loss (NCI109B) and increased kinase activity with USP22 addback (JHU-LX33) based on phosphorylation of kinase substrates. **(e)** Shown are significant kinase signatures ($FDR < 0.05$) in the SAL condition comparing control to USP22-perturbed for each model. (**f**) IHC analyses and representative images for γH2AX in sgCtrl versus sgUSP22 NCI109B at a 48-hour timepoint after treatment with either saline or cis-eto. Data are means $\pm$ SEM; n=6 tumors per treatment group, n=5 tumors for sgCtrl CIS-ETO. Scale bar, 10 μm. (**g**) IF representative images of γH2AX and DAPI for explanted cells from FHSC14 sgCtrl tumors treated in vivo with saline or cis-eto for 48 hours (images taken from Fig. 7e). White arrows point to γH2AX foci within a nucleus. Scale bar, 10 μm. Statistical analyses in **f** were performed by one-way ANOVA with Tukey's multiple comparisons test. Adjusted ANOVA p-values are shown on figure. NS=non-significant. Source Data is provided in the Source Data File.

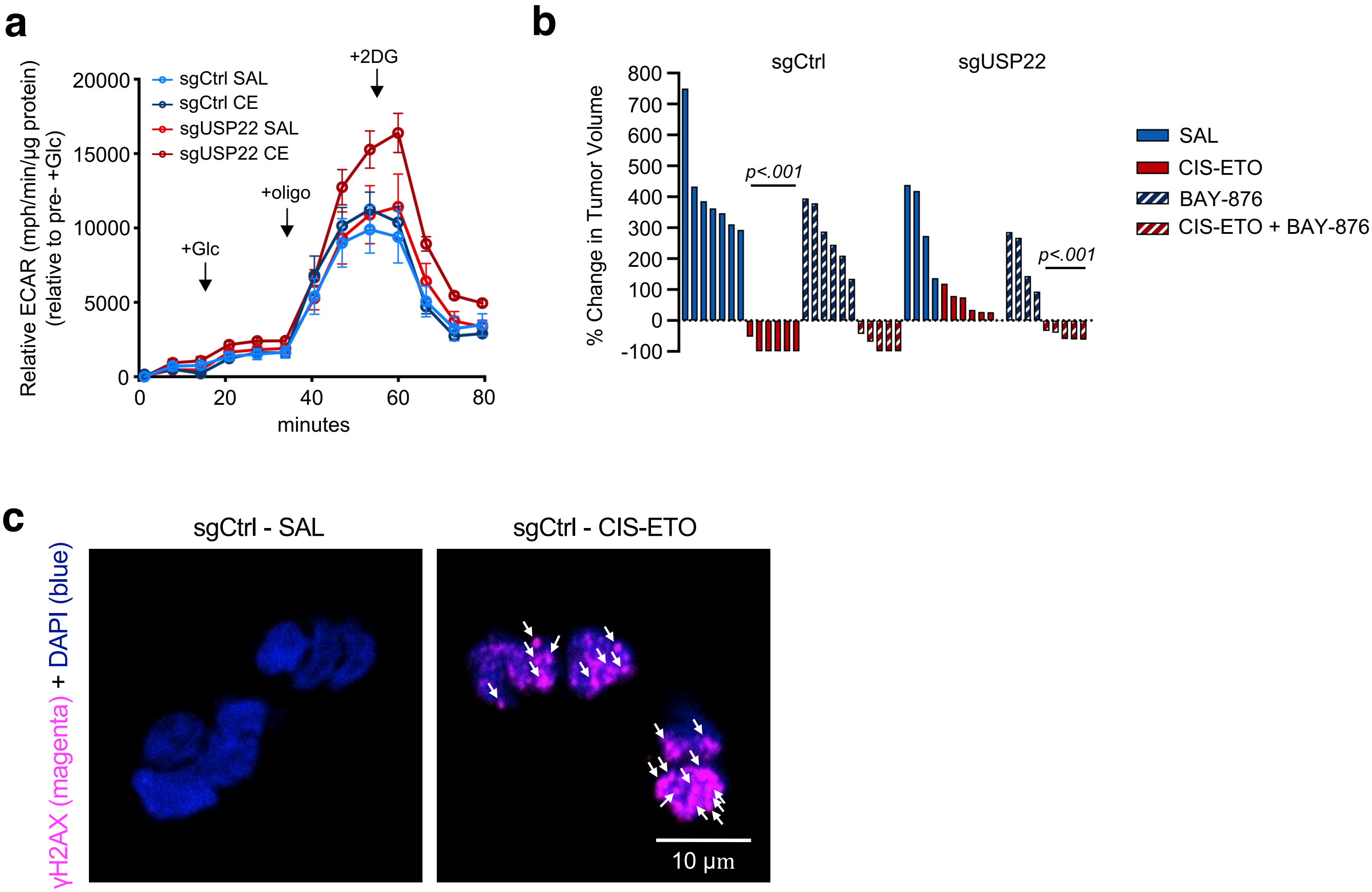

Supplementary Figure 10: Seahorse glycolytic stress test assay, quantification of tumor response to BAY-876 + chemotherapy combination treatment, and γH2AX IF analyses in FHSC14 sgUSP22 vs sgCtrl PDX models, related to Figure 8.

(**a**) Relative extracellular acidification rate (ECAR) traces (relative to pre- +Glc condition) of explanted tumor cells from FHSC14 sgUSP22 vs sgCtrl models treated in vivo with saline or cis-eto for 48 hours. Data are means ± SEM; n=3 tumors per group. Glc=glucose, oligo=oligomycin, 2DG=2-deoxy-D-glucose. (**b**) FHSC14 sgCtrl and sgUSP22 PDX models were treated with saline or cis-eto over 21 days. %Change in Tumor Volumes from Day 0 to Day 21 are quantified. Data for mice bearing tumors that did not survive to Day 21 of treatment are omitted. (**c**) IF representative images of γH2AX and DAPI for explanted cells from FHSC14 sgCtrl tumors treated in vitro with saline or cis-eto for 48 hours (images taken from Fig. 8e). White arrows point to γH2AX foci within nuclei. Scale bar, 10 µm. Statistical analyses in **b** comparing sgUSP22 CIS-ETO vs sgCtrl CIS-ETO groups and sgUSP22 CIS-ETO vs sgUSP22 CIS-ETO + BAY-876 groups were performed by unpaired student's two-sided t-test. Unadjusted t-test p-values are shown on figure. Source Data is provided in the Source Data File.
