# Supplementary material for: Loss of the USP22 deubiquitylase confers resistance to chemotherapy in small cell lung cancer
Source: Nat Commun. 2026 Jul 8;17:8402. doi: 10.1038/s41467-026-75117-2 (PMC13473660; doi:10.1038/s41467-026-75117-2)
Supplement: Supplementary file 2 [file 41467_2026_75117_MOESM2_ESM.pdf]

Title: Supplementary Data 1
Description: FHSC14 GeCKO v2 pilot genome scale sgRNA screen analysis and results. Top 500 genes enriched in the CIS-ETO vs. P0 condition are shown (ranked by positive adjusted p-value). Statistical analyses were performed by differential abundance analysis as described in CRISPRko Screen Analyses section in Methods. P-values are adjusted using Benjamini-Hochberg FDR procedure.

Title: Supplementary Data 2
Description: Gene composition and sgRNA sequences in the focused sgRNA library used for focused CRISPR deletion screens in this study.

Title: Supplementary Data 3
Description: NCI109B focused sgRNA screen gene-level analysis and results. Statistical analyses were performed by differential abundance analysis as described in CRISPRko Screen Analyses section in Methods. P-values are adjusted using Benjamini-Hochberg FDR procedure.

Title: Supplementary Data 4
Description: Enriched sgRNAs (log2FC > 2, FDR < 0.05) from the NCI109B CRISPR deletion screen. Statistical analyses were performed by differential abundance analysis as described in CRISPRko Screen Analyses section in Methods. P-values are adjusted using Benjamini-Hochberg FDR procedure.

Title: Supplementary Data 5
Description: sgRNA sequences used in this study for isogenic model generation

Title: Supplementary Data 6
Description: List of known USP22 mutations in SCLC

Title: Supplementary Data 7
Description: FHSC14 global proteomics analysis. Differentially-expressed proteins (FDR<0.05, Log2FC > 0.5 or Log2FC < -0.5) of sgUSP22 vs. sgCtrl in the CIS-ETO and SAL conditions. Statistical analyses were performed by linear regression model analysis as described in Global proteomics and phospho-proteomics analyses section in Methods. P-values are adjusted using Benjamini-Hochberg FDR procedure.

Title: Supplementary Data 8
Description: NCI109B global proteomics analysis. Differentially-expressed proteins (FDR<0.05, Log2FC > 0.5 or Log2FC < -0.5) of sgUSP22 vs. sgCtrl in the CIS-ETO and SAL conditions. Statistical analyses were performed by linear regression model analysis as described in Global proteomics and phospho-proteomics analyses section in Methods. P-values are adjusted using Benjamini-Hochberg FDR procedure.

Title: Supplementary Data 9
Description: RNA-seq Enrichr pathway analyses: MSigDB Hallmark. Statistical analyses were performed by gene enrichment analysis and fisher's exact test. P-values are adjusted using Benjamini-Hochberg FDR procedure.

Title: Supplementary Data 10
Description: RNA-seq Enrichr pathway analyses: GO Biological Process. Statistical analyses were performed by gene enrichment analysis and fisher's exact test. P-values are adjusted using Benjamini-Hochberg FDR procedure.

Title: Supplementary Data 11
Description: RNA-seq Enrichr pathway analyses: MSigDB WikiPathway. Statistical analyses were performed by gene enrichment analysis and fisher's exact test. P-values are adjusted using Benjamini-Hochberg FDR procedure.

Title: Supplementary Data 12
Description: RNA-seq Enrichr pathway analyses: MSigDB KEGG. Statistical analyses were performed by gene enrichment analysis and fisher's exact test. P-values are adjusted using Benjamini-Hochberg FDR procedure.

Title: Supplementary Data 13
Description: FHSC14 Proteomics vs RNA-seq to identify candidate non-histone substrates of USP22. Genes downregulated at the protein level ($Log2FC < -0.5$, $FDR < 0.05$ in GLBL Proteomics dataset) but not the RNA level ($\neq Log2FC < 0$ with $FDR < 0.05$ in RNA-seq dataset) for each treatment condition in FHSC14 sgUSP22 vs sgCtrl tumors

Title: Supplementary Data 14
Description: NCI109B Proteomics vs RNA-seq to identify candidate non-histone substrates of USP22. Genes downregulated at the protein level ($Log2FC < -0.5$, $FDR < 0.05$ in GLBL Proteomics dataset) but not the RNA level ($\neq Log2FC < 0$ with $FDR < 0.05$ in RNA-seq dataset) for each treatment condition in FHSC14 sgUSP22 vs sgCtrl tumors

Title: Supplementary Data 15
Description: FHSC14 CUT&Tag analysis - H2AK119ub. Differential peaks (FDR<0.05) of sgUSP22 vs. sgCtrl in the CIS-ETO and SAL conditions. Peaks with Log2FC > 0 and FDR < 0.05 are enriched in sgUSP22. Statistical analyses were performed by differential peak analysis as described in CUT&Tag section in Methods. P-values are adjusted using Benjamini-Hochberg FDR procedure.

Title: Supplementary Data 16
Description: FHSC14 CUT&Tag RNA-seq integrated analysis - H2AK119ub. Genes with enriched H2AK119ub peaks (Log2FC > 0, FDR < 0.05) with concurrent downregulated RNA (Log2FC < 0, FDR < 0.05) in sgUSP22 vs. sgCtrl in SAL and CIS-ETO condition.

Title: Supplementary Data 17
Description: FHSC14 phospho-proteomics analysis. Differentially-expressed phospho-sites (FDR<0.05, Log2FC > 0.5 or Log2FC < -0.5) of sgUSP22 vs. sgCtrl in the CIS-ETO and SAL conditions. Statistical analyses were performed by linear regression model analysis as described in Global proteomics and phospho-proteomics analyses section in Methods. P-values are adjusted using Benjamini-Hochberg FDR procedure.

Title: Supplementary Data 18
Description: NCI109B phospho-proteomics analysis. Differentially-expressed phospho-sites (FDR<0.05, Log2FC > 0.5 or Log2FC < -0.5) of sgUSP22 vs. sgCtrl in the CIS-ETO and SAL conditions. Statistical analyses were performed by linear regression model analysis as described in Global proteomics and phospho-proteomics analyses section in Methods. P-values are adjusted using Benjamini-Hochberg FDR procedure.

Title: Supplementary Data 19
Description: JHU-LX33 phospho-proteomics analysis. Differentially-expressed phospho-sites (unadjusted p-value < 0.01, Log2FC > 0.5 or Log2FC < -0.5) of USP22 vs. Empty vector control in the CIS-ETO and SAL conditions. Statistical analyses were performed by linear regression model analysis as described in Global proteomics and phospho-proteomics analyses section in Methods. P-values are adjusted using Benjamini-Hochberg FDR procedure.
