# Supplementary material for: Loss of the USP22 deubiquitylase confers resistance to chemotherapy in small cell lung cancer
Source: Nat Commun. 2026 Jul 8;17:8402. doi: 10.1038/s41467-026-75117-2 (PMC13473660; doi:10.1038/s41467-026-75117-2)
Supplement: Supplementary file 3 [file 41467_2026_75117_MOESM4_ESM.pdf]

nature portfolio

Corresponding author(s): David MacPherson

Last updated by author(s): Jun 12, 2026

Reporting Summary

Nature Portfolio wishes to improve the reproducibility of the work that we publish. This form provides structure for consistency and transparency in reporting. For further information on Nature Portfolio policies, see our Editorial Policies and the Editorial Policy Checklist.

Statistics

For all statistical analyses, confirm that the following items are present in the figure legend, table legend, main text, or Methods section.

n/a	Confirmed	
☐	☒	The exact sample size (*n*) for each experimental group/condition, given as a discrete number and unit of measurement
☐	☒	A statement on whether measurements were taken from distinct samples or whether the same sample was measured repeatedly
☐	☒	The statistical test(s) used AND whether they are one- or two-sided<br>*Only common tests should be described solely by name; describe more complex techniques in the Methods section.*
☒	☐	A description of all covariates tested
☐	☒	A description of any assumptions or corrections, such as tests of normality and adjustment for multiple comparisons
☐	☒	A full description of the statistical parameters including central tendency (e.g. means) or other basic estimates (e.g. regression coefficient) AND variation (e.g. standard deviation) or associated estimates of uncertainty (e.g. confidence intervals)
☐	☒	For null hypothesis testing, the test statistic (e.g. F, t, r) with confidence intervals, effect sizes, degrees of freedom and P value noted<br>*Give P values as exact values whenever suitable.*
☒	☐	For Bayesian analysis, information on the choice of priors and Markov chain Monte Carlo settings
☒	☐	For hierarchical and complex designs, identification of the appropriate level for tests and full reporting of outcomes
☒	☐	Estimates of effect sizes (e.g. Cohen's *d*, Pearson's *r*), indicating how they were calculated

Our web collection on statistics for biologists contains articles on many of the points above.

Software and code

Policy information about availability of computer code

Data collection	Image Studio v5.2 was used for western blot image acquisition. NIS-Elements AR 5.42.01 was used for immunohistochemistry image acquisition. Fusion 2.4.0.14 was used for immunofluorescence image acquisition. Sony MA900 software was used for flow cytometry data acquisition. Seahorse XF96 Analyzer software was used for Seahorse assay data acquisition.
Data analysis	The CRISPRko screen analysis was conducted using MAGeCK version 0.5.7 (pilot genome scale screen) and version 0.5.9 (focused library screen) (https://sourceforge.net/p/mageck/wiki/Home/). The global proteomics and phospho-proteomics analyses were conducted using the R package DreamAI version 0.1.0 (https://github.com/WangLab-MSSM/DreamAI) for imputation and the lm function in the base R package, version 4.2.0 (https://www.r-project.org/). Post-translational modification signature enrichment analysis was conducted using the R package ssGSEA2 version 1.0.0 (https://github.com/nicolerg/ssGSEA2). The PTMsigDB version 2.0.0 database was used to map phospho-sites to kinases. Output from these packages was plotted using the R packages ggplot2 version 3.3.5, ggh4x version 0.2.6, and ggrepel version 0.9.1. The R package dplyr version 1.0.8 and other related packages under the tidyverse package version 1.3.1 were used for basic data processing. R version 4.2.0 was used to run all R packages. ImageJ2 v2.16.0/1.52p was used to analyze and quantify immunohistochemistry, immunoflourescence, and western blot images. FlowJo v10 was used to analyzed flow cytometry.

For manuscripts utilizing custom algorithms or software that are central to the research but not yet described in published literature, software must be made available to editors and reviewers. We strongly encourage code deposition in a community repository (e.g. GitHub). See the Nature Portfolio guidelines for submitting code & software for further information.

Data

Policy information about availability of data

All manuscripts must include a data availability statement. This statement should provide the following information, where applicable:

- Accession codes, unique identifiers, or web links for publicly available datasets
- A description of any restrictions on data availability
- For clinical datasets or third party data, please ensure that the statement adheres to our policy

The LC-MS/MS proteomics data are deposited to the ProteomeXchange Consortium110 via the PRIDE partner repository with the dataset identifier PXD048813 [https://proteomecentral.proteomexchange.org/?pxid=PXD048813]. The RNA-seq data and CUT&Tag data are deposited in dbGAP under the accession number phs004087.v2.p1 [https://dbgap.ncbi.nlm.nih.gov/beta/search/?OBJ=study&TERM=phs004087.v2.p1].

Research involving human participants, their data, or biological material

Policy information about studies with human participants or human data. See also policy information about sex, gender (identity/presentation), and sexual orientation and race, ethnicity and racism.

Reporting on sex and gender	N/A
Reporting on race, ethnicity, or other socially relevant groupings	N/A
Population characteristics	N/A
Recruitment	N/A
Ethics oversight	N/A

Note that full information on the approval of the study protocol must also be provided in the manuscript.

Field-specific reporting

Please select the one below that is the best fit for your research. If you are not sure, read the appropriate sections before making your selection.

☒ Life sciences ☐ Behavioural & social sciences ☐ Ecological, evolutionary & environmental sciences

For a reference copy of the document with all sections, see nature.com/documents/nr-reporting-summary-flat.pdf

Life sciences study design

All studies must disclose on these points even when the disclosure is negative.

Sample size	The sample size for each figure is provided in the Figures or Figure Legends. No sample size calculation was performed. Sample sizes were based on previous experimental experience or previous published studies demonstrated to be sufficient to generate statistically significant results.
Data exclusions	No animals, samples, or other data points were excluded from analyses.
Replication	Biological replicates were used in all mouse experiments.
Randomization	Mice were randomly assigned to treatment groups until pre-determined sample sizes per treatment group were met.
Blinding	Blinding was not used during mouse therapeutic studies. Investigators were blinded during image acquisition and analysis for all immunohistochemistry and immunofluorescence studies.

Reporting for specific materials, systems and methods

We require information from authors about some types of materials, experimental systems and methods used in many studies. Here, indicate whether each material, system or method listed is relevant to your study. If you are not sure if a list item applies to your research, read the appropriate section before selecting a response.

Materials & experimental systems

n/a	Involved in the study
☐	☒ Antibodies
☐	☒ Eukaryotic cell lines
☒	☐ Palaeontology and archaeology
☐	☒ Animals and other organisms
☒	☐ Clinical data
☒	☐ Dual use research of concern
☒	☐ Plants

Methods

n/a	Involved in the study
☐	☒ ChIP-seq
☐	☒ Flow cytometry
☒	☐ MRI-based neuroimaging

Antibodies

Antibodies used

anti-USP22 (Novus, NBP1-49644, 1:500), anti-NKX-2 (Proteintech, 13013-1-AP, 1:1000), anti-SEMA6A (ThermoFisher, PA5-81009, 1:1000), anti-ASCL1 (BD Biosciences, 556604, 1:500), anti-INSM1 (Santa Cruz, sc-271408, 1:1000), anti-phospho-RAD50 S635 (Cell Signaling, 14223, 1:1000), anti-RAD50 (Cell Signaling, 3427, 1:1000), anti-phospho-MRE11 S676 (Cell Signaling, 4859, 1:1000), anti-MRE11 (Cell Signaling, 4847, 1:1000), anti-phospho-Chk1 S345 (Cell Signaling, 2348, 1:1000), anti-Chk1 (Cell Signaling, 2360, 1:1000), anti-HSP90 (Santa Cruz, sc-13119, 1:1000), anti-GAPDH (Santa Cruz, sc-32233, 1:1000), anti-alpha-Tubulin (Cell Signaling, 3873, 1:1000), anti-rabbit IgG HRP-linked (Cell Signaling, 7074, 1:2000), anti-mouse IgG HRP-linked (Cell Signaling, 7076, 1:2000), anti-phospho-histone H3 S10 (Millipore, 06-570, 1:200), anti-cleaved caspase-3 N175 (Cell Signaling, 9661, 1:100), anti-phospho-histone H2A.X S139 (Cell Signaling, 9718, 1:100), biotinylated goat anti-rabbit IgG secondary antibody (Vector Laboratories, BP-9100-50, 1:100), anti-phospho-histone H2A.X S139 (Cell Signaling, 80312, 1:200), anti-mouse AlexaFluor 647 (ThermoFisher, A-31571, 1:1000), ubiquityl-histone H2A K119 (Cell Signaling, 8240, 1:100 or 1:1000), tri-methyl-histone H3 K27 (Cell Signaling, 9733, 1:100), normal Rabbit IgG (Cell Signaling, 2729, 1:100), anti-ASCL1 (Cell Signaling, 10585, 1:200), anti-NEUROD1 (Cell Signaling, 59418, 1:400), anti-SYP (Cell Signaling, 36406, 1:100), anti-CHGA (Novus Biologicals, NB120-15160, 1:500), anti-histone H2A (Cell Signaling, 12349, 1:1000), anti-histone H3 (Cell Signaling, 4499, 1:1000).

Validation

All antibodies were purchased from manufacturers and were validated by manufacturers with a validation statement on manufacturer's website for the application in which they were used for this study.

Antibodies validated for Western Blotting: anti-USP22 (Novus, NBP1-49644), anti-NKX-2 (Proteintech, 13013-1-AP), anti-SEMA6A (ThermoFisher, PA5-81009, anti-ASCL1 (BD Biosciences, 556604), anti-INSM1 (Santa Cruz, sc-271408), anti-phospho-RAD50 S635 (Cell Signaling, 14223), anti-RAD50 (Cell Signaling, 3427), anti-phospho-MRE11 S676 (Cell Signaling, 4859, anti-MRE11 (Cell Signaling, 4847), anti-phospho-Chk1 S345 (Cell Signaling, 2348), anti-Chk1 (Cell Signaling, 2360), anti-HSP90 (Santa Cruz, sc-13119), anti-GAPDH (Santa Cruz, sc-32233, anti-alpha-Tubulin (Cell Signaling, 3873), anti-rabbit IgG HRP-linked (Cell Signaling, 7074), anti-mouse IgG HRP-linked (Cell Signaling, 7076), anti-histone H2A (Cell Signaling, 12349), anti-histone H3 (Cell Signaling, 4499).

Antibodies validates for immunohistochemistry: anti-phospho-histone H3 S10 (Millipore, 06-570), anti-cleaved caspase-3 N175 (Cell Signaling, 9661), anti-phospho-histone H2A.X S139 (Cell Signaling, 9718), biotinylated goat anti-rabbit IgG secondary antibody (Vector Laboratories, BP-9100-50), anti-ASCL1 (Cell Signaling, 10585), anti-NEUROD1 (Cell Signaling, 59418), anti-SYP (Cell Signaling, 36406), anti-CHGA (Novus Biologicals, NB120-15160)
.
Antibodies validated for immunofluorescence: anti-phospho-histone H2A.X S139 (Cell Signaling, 80312), anti-mouse AlexaFluor 647 (ThermoFisher, A-31571)

Antibodies validated for CUT&Tag: ubiquityl-histone H2A K119 (Cell Signaling, 8240), tri-methyl-histone H3 K27 (Cell Signaling, 9733), normal Rabbit IgG (Cell Signaling, 2729).

Eukaryotic cell lines

Policy information about cell lines and Sex and Gender in Research

Cell line source(s)	293FT cell line (System Bioscience, LV900A-1); female
Authentication	293FT cell line was not authenticated.
Mycoplasma contamination	293FT cell line was mycoplasma negative during routine testing.
Commonly misidentified lines (See ICLAC register)	N/A

Animals and other research organisms

Policy information about studies involving animals; ARRIVE guidelines recommended for reporting animal research, and Sex and Gender in Research

Laboratory animals

NOD scid gamma mice (NSG, JAX strain 005557), ages 8-24 weeks. Mice were housed with no more than 5 animals per cage in a temperature and humidity controlled environment with a standard 12-hour dark/light cycle.

Wild animals	N/A
Reporting on sex	Patient-derived xenograft (PDX) models were injected subcutaneously into flanks of male and female NSG mice throughout the study.
Field-collected samples	N/A
Ethics oversight	Animal studies were approved by the Institutional Animal Care and Use Committee at Fred Hutch (protocol #50783).

Note that full information on the approval of the study protocol must also be provided in the manuscript.

Plants

Seed stocks	N/A
Novel plant genotypes	N/A
Authentication	N/A

ChIP-seq

Data deposition

☒ Confirm that both raw and final processed data have been deposited in a public database such as GEO.

☒ Confirm that you have deposited or provided access to graph files (e.g. BED files) for the called peaks.

Data access links
May remain private before publication.

The CUT&Tag data are deposited in dbGAP under the accession number phs004087.v2.p1 [https://dbgap.ncbi.nlm.nih.gov/beta/search/?OBJ=study&TERM=phs004087.v2.p1]

Files in database submission

H14_C1_IgGRab1_S1_R1_001.fastq.gz
H14_C2_IgGRab1_S2_R1_001.fastq.gz
H14_C3_IgGRab1_S3_R1_001.fastq.gz
H14_C1_H3me27_S4_R1_001.fastq.gz
H14_C2_H3me27_S5_R1_001.fastq.gz
H14_C3_H3me27_S6_R1_001.fastq.gz
H14_U1_H3me27_S7_R1_001.fastq.gz
H14_U2_H3me27_S8_R1_001.fastq.gz
H14_U3_H3me27_S9_R1_001.fastq.gz
H14ce_C1_H3me27_S10_R1_001.fastq.gz
H14ce_C2_H3me27_S11_R1_001.fastq.gz
H14ce_C3_H3me27_S12_R1_001.fastq.gz
H14ce_U1_H3me27_S13_R1_001.fastq.gz
H14ce_U2_H3me27_S14_R1_001.fastq.gz
H14ce_U3_H3me27_S15_R1_001.fastq.gz
H14_C1_H2ub119_S16_R1_001.fastq.gz
H14_C2_H2ub119_S17_R1_001.fastq.gz
H14_U1_H2ub119_S19_R1_001.fastq.gz
H14_U2_H2ub119_S20_R1_001.fastq.gz
H14ce_C1_H2ub119_S21_R1_001.fastq.gz
H14ce_C2_H2ub119_S22_R1_001.fastq.gz
H14ce_U1_H2ub119_S24_R1_001.fastq.gz
H14ce_U2_H2ub119_S25_R1_001.fastq.gz
H14_C1_IgGRab1_S1_R2_001.fastq.gz
H14_C2_IgGRab1_S2_R2_001.fastq.gz
H14_C3_IgGRab1_S3_R2_001.fastq.gz
H14_C1_H3me27_S4_R2_001.fastq.gz
H14_C2_H3me27_S5_R2_001.fastq.gz
H14_C3_H3me27_S6_R2_001.fastq.gz
H14_U1_H3me27_S7_R2_001.fastq.gz
H14_U2_H3me27_S8_R2_001.fastq.gz
H14_U3_H3me27_S9_R2_001.fastq.gz
H14ce_C1_H3me27_S10_R2_001.fastq.gz
H14ce_C2_H3me27_S11_R2_001.fastq.gz
H14ce_C3_H3me27_S12_R2_001.fastq.gz
H14ce_U1_H3me27_S13_R2_001.fastq.gz
H14ce_U2_H3me27_S14_R2_001.fastq.gz

H14ce_U3_H3me27_S15_R2_001.fastq.gz
H14_C1_H2ub119_S16_R2_001.fastq.gz
H14_C2_H2ub119_S17_R2_001.fastq.gz
H14_U1_H2ub119_S19_R2_001.fastq.gz
H14_U2_H2ub119_S20_R2_001.fastq.gz
H14ce_C1_H2ub119_S21_R2_001.fastq.gz
H14ce_C2_H2ub119_S22_R2_001.fastq.gz
H14ce_U1_H2ub119_S24_R2_001.fastq.gz
H14ce_U2_H2ub119_S25_R2_001.fastq.gz
H3K27me3.broad_peaks.bed
H2AK119ub.broad_peaks.bed

Genome browser session (e.g. UCSC): N/A

Methodology

Replicates: For CUT&Tag experiments performed in this study, 2-3 biological tumor replicates per treatment condition were sequenced. Peaks used were consensus peaks between biological replicates.

Sequencing depth: For the CUT&Tag experiment in this study, 539,250,558 total reads were sequenced with 14,230,042 Undermined reads. Sequencing depth wast least 3-7 million reads per sample. Reads were paired-end.

Antibodies: ubiquityl-histone H2A K119 (Cell Signaling, 8240, 1:100), tri-methyl-histone H3 K27 (Cell Signaling, 9733, 1:100), normal Rabbit IgG (Cell Signaling, 2729, 1:100)

Peak calling parameters: The nf-core cutandrun pipeline was followed for quality trimming using TrimGalore and alignment to GRCh38 reference genome using Bowtie2. MACS2 was used to call broad peaks with and without IgG controls and an intersection of the two was considered the final peak set for each sample. Read counts for each peak were generated using Subread featureCounts.

Data quality: For quality control, a combination of Samtools and the bedtools suite was used to calculate fragment length and number of reads in peaks.

Software: The following software was used to process, quality control, and analyze CUT&Tag data used in this study: the nf-core cutandrun pipeline, TrimGalore, MACS2, Samtools, Homer, Subread featureCounts. Differential peak expression between treatment groups was performed using Bioconductor edgeR. Integrative Genomics Viewer v2.18.497 was used to visualize peaks. References to each software tool are provided in the Materials and Methods section.

Flow Cytometry

Plots

Confirm that:

☒ The axis labels state the marker and fluorochrome used (e.g. CD4-FITC).

☒ The axis scales are clearly visible. Include numbers along axes only for bottom left plot of group (a 'group' is an analysis of identical markers).

☒ All plots are contour plots with outliers or pseudocolor plots.

☒ A numerical value for number of cells or percentage (with statistics) is provided.

Methodology

Sample preparation: Patient-derived xenograft (PDX) cells were transduced with lentivirus containing fluorescent ZsGreen selection marker. Cells were injected subcutaneously into the flanks of NSG mice. Once tumors formed, tumors were mechanically dissociated and enzymatically digested with Collagenase IV 1mg/mL solution for 30 minutes at 37 degrees Celsius. Red blood cells were lysed using ACK lysing buffer (ThermoFisher, A1049201) and remaining cells were strained to form single-cell suspensions.

Instrument: Sony MA900

Software: Sony MA900 Software; FlowJo 10

Cell population abundance: ZsGreen-positive cells were sorted and purity of post-sort fraction was determined either by immediately re-analyzing post-sort fraction on Sony MA900 (greater than 95% ZsGreen-positive) or analyzing the tumor single-cell suspensions formed from the purified ZsGreen-positive population on Sony MA900 (80-99% ZsGreen-positive).

Gating strategy: Untransduced parental patient-derived xenograft (PDX) cells served as negative controls for ZsGreen fluorescence. Cell population was gated using SSC-A vs FSC-A. Single cells were gated using FSC-H vs FSC-A. ZsGreen+ cells were gated using FITC-A vs PE-A. A figure showing the gating strategy for sorting ZsGreen+ cells using untransduced parental cells as negative controls is provided in the supplement and main figures.

☒ Tick this box to confirm that a figure exemplifying the gating strategy is provided in the Supplementary Information.
