# Supplementary material for: Loss of the USP22 deubiquitylase confers resistance to chemotherapy in small cell lung cancer
Source: Nat Commun. 2026 Jul 8;17:8402. doi: 10.1038/s41467-026-75117-2 (PMC13473660; doi:10.1038/s41467-026-75117-2)
Supplement: Supplementary file 4 [file 41467_2026_75117_MOESM5_ESM.pdf]

nature portfolio

Peer Review File

Loss of the USP22 deubiquitylase confers resistance to chemotherapy in small cell lung cancer

Corresponding Author: Dr David MacPherson

This file contains all reviewer reports in order by version, followed by all author rebuttals in order by version.

Version 0:

Reviewer comments:

Reviewer #1

(Remarks to the Author)
The authors demonstrate convincingly that loss of SAGA complex activity in SCLC PDX models, and specifically USP22, drives resistance to cisplatin plus etoposide, the backbone of first-line therapy for this disease. Furthermore, ectopic USP22 expression in a PDX with an inactivating mutation can restore chemosensitivity. Multiomic comparison of SCLC with or without USP22 demonstrate myriad effects of deletion, including decreased expression of neuroendocrine genes and upregulation of glycolysis. To the best of our knowledge, this is the first in vivo CRISPR screens to be performed in SCLC PDX models, and one of the few screens to be reported for any SCLC model, either in vivo or in vitro. It is an exceptional technical accomplishment to address a question of immediate clinical importance. This is an excellent paper that merits prompt publication.

The question of whether USP22 loss is recurrent in patients is both obvious and beyond the scope of this paper, as it will require a large number of samples from relapsed patients. The authors demonstrate that this can occur during normal tumor evolution with the model JHU-LX33. It would help if they could clarify whether this model was derived before or after the patient received chemotherapy.

Given the variety of individual molecular effects of USP22 loss, including loss of neuroendocrine expression, it is possible that a global shift in lineage is induced. There is precedent for this, as MYC amplification in SCLC has gross effects on cell morphology that drive a “variant” appearance resembling LCNEC, with abundant cytoplasm and more conspicuous nucleoli. Does something similar happen here? Our only request is that the authors compare the histologies of isogenic PDX tumors with or without USP22, preferably with pictures. Immunohistochemistry of the relevant clinical neuroendocrine markers (INSM1, chromogranin, synaptophysin, NCAM), as well as ASCL1 and NEUROD1 (in FHSC14 and NCI109B, respectively) would be helpful, as would comparison with JHU-LX33. Would a thoracic pathologist still diagnose these as USP22-negative tumors as SCLC? This should hopefully not require any additional mice, assuming fixed samples from previously grown xenografts are available. Either answer is useful to classify the type of resistance that USP22 inactivation confers. It would be interesting and surprising if there were such a large change in neuroendocrine gene expression but no effect on histology.

Reviewer #2

(Remarks to the Author)
This manuscript by Best et al. investigates chemotherapy resistance in small cell lung cancer (SCLC) using patient-derived xenograft (PDX) models and in vivo CRISPR-Cas9 screening. The study identifies the deubiquitinase USP22, a core enzymatic component of the SAGA mDUB complex, as a determinant of therapy response in both ASCL1-positive and NeuroD-positive SCLC subtypes.
The in vivo CRISPR screen is carefully designed and statistically robust, and the follow-up experiments convincingly show that USP22 loss confers therapy resistance and alters tumor biology. The work is particularly significant because it challenges the oversimplified view in the literature that USP22 is universally tumor-promoting, instead revealing a context-dependent role in therapy response. Moreover, the data showing that USP22 deficiency impairs activation of DNA damage response (DDR) proteins without changing their expression levels is both mechanistically intriguing and therapeutically relevant. As such the findings are potentially highly interesting and patient relevant.

Overall, the manuscript is very strong and highly interesting, with clear potential for publication in Nature Communications. Its main limitation is that the mechanistic insight into how USP22 mediates these effects remains limited.

Major Strengths
1. Use of highly relevant patient-derived models. The study uses multiple SCLC PDX models representing the ASCL1-positive and NeuroD-positive subtypes, which enhances the translational relevance and ensures the findings reflect patient biology rather than cell-line-specific phenomena.
2. Robust in vivo CRISPR-Cas9 screening. The in vivo screening is carefully executed with strong statistical support, leading to the confident identification of USP22 as a key mediator of therapy resistance. Figures 1–3, in particular, are clear and compelling.
3. Clear link to DNA damage response activation. The authors convincingly demonstrate that USP22 loss impairs the activation of DDR proteins while leaving their total expression unchanged. This adds an important mechanistic layer and highlights how chromatin-associated factors can regulate therapy sensitivity independently of gene expression changes.
4. High-quality data challenging simplistic literature. The work provides a strong counterpoint to the predominantly low-quality literature that describes USP22 as universally oncogenic, often by stabilizing MYC or similar proteins. The present study demonstrates a more nuanced, context-dependent biology supported by rigorous in vivo data.

Major Concerns and Suggestions
1. Mechanistic insight into USP22 function is limited. The mechanistic basis of USP22's effects remains incompletely resolved. The CUT&Tag data for H2A monoubiquitination (Figure 6G) show roughly balanced increases and decreases in peak intensity, with no strong unidirectional pattern. Global H2A monoubiquitination levels are not presented (e.g., by Western blot), and without occupancy data showing where USP22 resides on chromatin, it is difficult to claim a direct transcriptional regulatory role. The current data could be consistent with indirect effects rather than a primary chromatin-driven mechanism.
2. Underutilization of mass spectrometry data and likely indirect mechanism. The mass spectrometry data presented in Figure 4 are highly interesting but remain underexplored. Based on prior experience with USP22 and SAGA biology, it is plausible that USP22's major functional role in this context is not as a direct chromatin regulator but rather through binding and stabilizing specific protein substrates. Such substrates could include transcription factors, cofactors, or other regulatory proteins that in turn mediate the downstream changes in DDR activation, therapy resistance, and possibly even the metabolic effects observed. Many previous attempts to connect USP22's phenotypes directly to H2A/H2B deubiquitination have been inconclusive, suggesting that the primary functional effects may instead arise through non-histone substrates. Mining the mass spectrometry dataset for proteins whose abundance or stability depends on USP22 would likely identify candidate mediators of the phenotypes observed and could provide a coherent mechanistic model.
3. Limited integration with other SAGA/mDUB components. The manuscript would be substantially strengthened by including analyses that compare USP22 loss to the depletion of other mDUB components such as ATXN7L3 or ENY2, or by testing whether inhibition of the SAGA acetyltransferase module yields similar or divergent phenotypes. Even ex vivo or in vitro experiments would help determine whether the observed therapy resistance is specific to USP22 or reflects a broader SAGA/mDUB functional axis.
4. Glycolysis data feel conceptually disconnected. The findings involving glycolytic inhibition in Figures 7 and 8 are interesting but not clearly integrated into the central mechanistic narrative. Connecting the metabolic findings more explicitly to the therapy-resistance phenotype would strengthen the story. For example, demonstrating that BAY876 treatment reproduces the effects observed in Figures 7F–G, or providing a clear rationale linking glycolytic modulation to impaired DDR activation, would help unify the manuscript.
5. Patient data. It is somewhat surprising that, given the very interesting in vivo data and potential patient relevance, that the authors do not provide any patient-derived data related to USP22 in SCLC. It would be interesting to know (either through TCGA analyses or IHC staining of patient samples) whether USP22 may also be lost in a subset of SCLC patients.
Overall, this study provides very interesting and solid in vivo screening results in highly relevant model systems. The findings are generally well presented, clear and convincing. However, the actual mechanism(s) by which USP22 functions in SCLC remain largely unclear. The authors have generated extensive proteome and transcriptome datasets which have only been superficially analyzed, which may, in fact, potentially be exploited to find more detailed mechanistic insights into USP22 biology in SCLC.

Reviewer #3

(Remarks to the Author)
Best et al. performed a targeted in vivo screen in PDX models of small cell lung cancer (SCLC) to understand the mechanisms of chemo-resistance. The authors identified USP22, a part of SAGA complex as a key molecule whose deletion conferred resistance to PDX models. They further show that USP22 loss increased H2A-K119 monoubiquitylation and further attenuated DNA damage driven-phosphorylation events and apoptosis. In addition, they show glycolysis could be key targetable vulnerability in USP22 null tumors.
Overall, the manuscript is well written, and the data is presented clearly with a few draw backs. The use of 3 different PDX lines (USP22 positive and null) adds strong evidence for the role of the protein, both knock out and rescue experiments are well done. The first couple of figures clearly establish the role of USP22 in SCLC chemoresistance with multiple experiments and strong data.
The findings are novel and impactful, linking USP22 loss to chemoresistance through epigenetic and metabolic reprogramming. The large amount of omics data further serve as a valuable resource for the community.

However, despite the strong data, the paper may also need mechanistic depth and better integration of stories. In light of the standards of Nature Communications, a few experiments and clarifications are needed to strengthen the mechanistic

insights and translational relevance. In brief, the authors can further transform it from a "USP22 is important" study to a "USP22 connects epigenetics, metabolism, and DDR in chemoresistance" breakthrough.

Major Concerns and Required Experiments

1. General Storyline – Lack of Cohesive Narrative Between Key Findings

Issue: The paper presents several independent observations (e.g., USP22 loss → H2AK119ub → neuronal gene suppression; glycolysis upregulation; DDR impairment), but these stories feel disconnected. The authors could mechanistically link these pathways to show how they collectively drive chemoresistance. This reviewer also acknowledges that sometimes connecting these different mechanisms could require more experiments and could be beyond the scope of this study. As such, a strong discussion paragraph should be added about the disconnect/shortcomings of the study. Alternatively if the authors could provide a cohesive connection and narrative, the manuscript will be strengthened.

2. Mechanistic Link Between USP22 Loss and Chemoresistance

Issue: While the study demonstrates that USP22 loss leads to chemoresistance, the direct mechanistic link between USP22's deubiquitylase activity and the observed phenotypic changes (e.g., H2AK119ub, glycolysis, DNA damage response) needs further specific validation. For example, rescue experiments with USP22 catalytic mutant USP22-C185S to show that catalytic activity is required for chemosensitivity is well done. However, additional mutants (e.g., USP22 lacking H2A/H2B deubiquitylation specificity) could be tested to dissect whether H2AK119ub or other substrates are critical. Alternatively, functional validation of H2AK119ub targets by using CRISPR/dCas9 to tether ubiquitin ligases (e.g., RING1B) or deubiquitylases to neuronal gene promoters in USP22-null cells to test if H2AK119ub deposition alone drives chemoresistance. There are other possible experiments to show and connect these by functional validation. The reviewer also acknowledges that not all experiments are required. Showing one experiment to connect the specificity could also address the shortcomings of cohesive narrative (point 1).

2. Glycolysis as a Therapeutic Vulnerability

Issue: The GLUT1 inhibitor BAY-876 resensitises USP22-null tumors, but the metabolic dependency is not fully characterized. Metabolomic profiling could be used to compare metabolite levels (e.g., lactate, ATP, TCA intermediates) in USP22-null vs. wild-type tumors ± cisplatin/etoposide to confirm glycolytic flux changes. Alternatively, genetic inhibition of glycolysis by Knockdown or knockout of HK2 or SLC2A1 (GLUT1) in USP22-null tumors to validate that glycolytic genes are essential for chemoresistance. Other alternative could be testing epigenetic-metabolic crosstalk: Treat USP22-null cells with H2AK119ub inhibitors (e.g., PRC1/2 inhibitors like GSK126 or UNC1999) and measure glycolytic gene expression (e.g., GLUT1, HK2) to test if H2AK119ub directly regulates metabolic reprogramming. Another possibility to test DDR-glycolysis connection: Treat USP22-null cells with 2-deoxyglucose (2-DG, glycolysis inhibitor) and measure γH2AX or RAD51 foci to test if glycolysis suppression restores DNA damage sensitivity. Alternatively, Test if LDHA knockdown (blocks lactate production) rescues cisplatin sensitivity in USP22-null cells.

These are multiple experiments and reviewer acknowledges that some of these could be beyond the scope of the current work. However, performing at least one of the proposed experiments that is feasible for authors in a quick span of time and show the connection could strengthen the manuscript and also connect the narrative cohesively.

4. Clinical Relevance and Translation: The study could be strengthened by showing USP22 loss occurs in relapsed SCLC patients or correlates with poor outcomes. It could be done by analyzing clinical cohorts (if available): Perform IHC or targeted sequencing for USP22 mutations/loss in pre- and post-chemotherapy SCLC patient samples (e.g., from TCGA or institutional cohorts). Correlation with survival: If patient data is available, assess whether USP22 loss/mutation correlates with progression-free or overall survival. Single-cell RNA-seq: Analyse published datasets (e.g., from George et al., 2024) to determine if USP22-low cells are enriched in relapsed SCLC. From any of the above 3 proposed analysis, the feasible one could be done and it could strengthen the clinical relevance.

Minor Concerns and Clarifications

1. Figure 1: May please clarify how sgRNA enrichment was normalised across pools. E.g. were batch effects considered?
2. Figure 2: Include quantification of tumour regression (e.g., % complete response) in addition to growth curves.
3. Figure 5: Provide RNA-seq validation (e.g., qPCR) for key neuronal genes (ASCL1, NKX2-2).
4. Figure 7: Show representative images for γH2AX foci in immunofluorescence assays.
5. Discussion: Address why USP22 loss has stronger effects in FHSC14 (ASCL1-high) vs. NCI109B (NEUROD1-high). Is this subtype-specific?

Conclusion

The manuscript identifies USP22 as a critical regulator of SCLC chemoresistance with compelling in vivo data. However, the mechanistic gaps and lack of clinical validation limit its impact. Addressing these issues would solidify the study's significance for *Nature Communications*. Specifically:

1. Prove USP22's direct role in H2AK119ub and gene silencing.
2. Validate glycolysis as a targetable vulnerability with orthogonal approaches.
3. Link USP22 loss to clinical chemoresistance in patient samples.

Reviewer #4

(Remarks to the Author)
I co-reviewed this manuscript with one of the reviewers who provided the listed reports. This is part of the Nature Communications initiative to facilitate training in peer review and to provide appropriate recognition for Early Career Researchers who co-review manuscripts.

Version 1:

Reviewer comments:

Reviewer #1

(Remarks to the Author)
This was already an excellent manuscript on initial submission, and the authors have improved it substantially. They have now shown that USP22-negative tumors remain histologically consistent with SCLC despite reduced expression of neurodevelopmental transcription factors such as ASCL1, NEUROD1, and INSM1. This is a significant mechanistic insight that distinguishes acquired chemoresistance through loss of SAGA complex activity from other alterations, such as MYC amplification, that induce substantial histologic transformation. Of course, there are further questions to answer, such as the role of other SAGA complex components and the critical targets of USP22 for chemosensitivity, but without an in vitro model that fully recapitulates clinical chemoresistance, which does not exist for SCLC, these experiments will need to be performed in vivo in PDX models and will take years. This story is of general interest and is ready to be reported in its current form to the lung cancer research community.

Reviewer #2

(Remarks to the Author)

Overall, my enthusiasm for this manuscript remains. The study provides very interesting and solid data. However, the authors side stepped and still have not addressed one of my major concerns. In my first comment I specifically mentioned that it would be important to analyze global H2A monoubiquitination levels (i.e. by Western blot). Given the strong emphasis placed on this modification in the manuscript (and little consideration of altnernative mechanisms), I would consider this to be important (regardless of the outcome). Furthermore, it does not seem unreasonable to also test the extent to which other SAGA components may be involved. For example, given the authors' findings, one may expect that enzymatic inhibition of GCN5 (e.g., using CPTH6) may elicit similar effects as USP22. And even if not, this would be interesting.

Note: In comment 3, reviewer 3 mentioned inhibition of EZH2 (PRC2 component) as "H2K119ub inhibitors". Although PRC2 and PRC1 are clearly intertwined, I would certainly not characterize EZH2 inhibition as "H2K119ub inhibition".

Reviewer #3

(Remarks to the Author)
Dear Editor,

I thank the authors for their thoughtful and comprehensive response to the comments and suggestions. I appreciate the significant efforts they have made to address the points raised, and I am pleased with the additional data and clarifications they have provided.

I am satisfied with the revisions, particularly the strengthened connection between glycolysis upregulation and impaired DNA damage response in USP22-null models, along with the added histological and immunohistochemical data, and expanded analysis of proteomic and transcriptomic datasets that further solidify the clinical relevance of the findings. I understand the practical considerations and time constraints involved in generating additional genetic models or performing certain mechanistic experiments in PDX systems, and I agree that the new experiments they have included meaningfully enhance the narrative and mechanistic insight of the study. I appreciate the expanded discussion regarding the potential subtype-specific effects and the limitations of linking H2AK119ub directly to gene silencing in this context.

I thank author’s effort to improve the previously fragmented core mechanistic narrative linking USP22 loss to chemoresistance. Although connections between glycolysis and DDR impairment have been strengthened, my very minor concern remains as the seemingly absent unified model explaining how USP22’s deubiquitinase activity coordinately regulates neuroendocrine identity, metabolic reprogramming, and DNA damage response. While I completely understand the practical constraints, to be critical, the absence of rescue experiments using substrate-specific USP22 mutants or direct modulation of H2AK119ub at target gene loci leaves open the question of whether the observed phenotypes are direct consequences of USP22 catalytic activity or indirect effects of SAGA complex destabilization.

That being said, I believe that the paper meets the rigor of Nature Communications, provided the limitations are transparently acknowledged in the discussion, as the authors have begun to do.

Overall, I believe the manuscript has been substantially improved and now presents a more cohesive and compelling story.

Reviewer #4

(Remarks to the Author)
I co-reviewed this manuscript with one of the reviewers who provided the listed reports. This is part of the Nature Communications initiative to facilitate training in peer review and to provide appropriate recognition for Early Career Researchers who co-review manuscripts.

Open Access This Peer Review File is licensed under a Creative Commons Attribution 4.0 International License, which permits use, sharing, adaptation, distribution and reproduction in any medium or format, as long as you give appropriate credit to the original author(s) and the source, provide a link to the Creative Commons license, and indicate if changes were made.
In cases where reviewers are anonymous, credit should be given to 'Anonymous Referee' and the source.
The images or other third party material in this Peer Review File are included in the article's Creative Commons license, unless indicated otherwise in a credit line to the material. If material is not included in the article's Creative Commons license and your intended use is not permitted by statutory regulation or exceeds the permitted use, you will need to obtain permission directly from the copyright holder.
To view a copy of this license, visit https://creativecommons.org/licenses/by/4.0/

RESPONSE to REVIEWER COMMENTS

We thank the reviewers for their thorough and supportive review of our manuscript address the major points raised below:

Reviewer #1 - SCLC resistance (Remarks to the Author):

The authors demonstrate convincingly that loss of SAGA complex activity in SCLC PDX models, and specifically USP22, drives resistance to cisplatin plus etoposide, the backbone of first-line therapy for this disease. Furthermore, ectopic USP22 expression in a PDX with an inactivating mutation can restore chemosensitivity. Multiomic comparison of SCLC with or without USP22 demonstrate myriad effects of deletion, including decreased expression of neuroendocrine genes and upregulation of glycolysis. To the best of our knowledge, this is the first in vivo CRISPR screens to be performed in SCLC PDX models, and one of the few screens to be reported for any SCLC model, either in vivo or in vitro. It is an exceptional technical accomplishment to address a question of immediate clinical importance. This is an excellent paper that merits prompt publication.

The question of whether USP22 loss is recurrent in patients is both obvious and beyond the scope of this paper, as it will require a large number of samples from relapsed patients. The authors demonstrate that this can occur during normal tumor evolution with the model JHU-LX33. It would help if they could clarify whether this model was derived before or after the patient received chemotherapy.

We thank the reviewer for the enthusiastic assessment of the strengths of our manuscript and now clarify on page 17 that the LX33 model was from a chemonaïve patient, citing Hann et al.

Given the variety of individual molecular effects of USP22 loss, including loss of neuroendocrine expression, it is possible that a global shift in lineage is induced. There is precedent for this, as MYC amplification in SCLC has gross effects on cell morphology that drive a "variant" appearance resembling LCNEC, with abundant cytoplasm and more conspicuous nucleoli. Does something similar happen here?

This is an important point raised by the reviewer. Hemeatoxylin and Eosin (H+E) stained tumors from our three genetically perturbed SCLC PDX models with *USP22* deletion or re-expression (NEW Supplemental Fig. 7a-c) were reviewed by Dr. Haodong Xu, a clinical lung cancer pathologist, and were all found to be diagnostic of small cell lung carcinoma. Differences in histological features with USP22 perturbation, including shift to a variant histology were not apparent. Dr. Xu has been added to the author list.

Our only request is that the authors compare the histologies of isogenic PDX tumors with or without USP22, preferably with pictures. Immunohistochemistry of the relevant clinical neuroendocrine markers (INSM1, chromogranin, synaptophysin, NCAM), as well as ASCL1 and NEUROD1 (in FHSC14 and NCI109B, respectively) would be helpful, as would comparison with JHU-LX33.

We have added H+Es for all three of our genetically perturbed SCLC PDX models, along with IHC data where we tested impact of USP22 loss on expression of neuroendocrine markers ASCL1, NEUROD1, INSM1 and SYP (NEW Supplemental Fig. 7a-e). In the ASCL1-high expressing FHSC14 model, both ASCL1 and INSM1 expression were decreased in sgUSP22 tumors, consistent with our previous RNA-seq and Western blot data showing decreased expression of *ASCL1* and *INSM1*. Similarly, in the NEUROD1-high expressing model NCI109B, we observed a decrease in NEUROD1 and INSM1 associated with *USP22* deletion. However, no difference in NEUROD1 or INSM1 expression was observed between the JHU-LX33 Empty vector (USP22-null) vs the USP22 add-back models. Markers SYP and CHGA were unchanged with respect to USP22 status.

Would a thoracic pathologist still diagnose these as USP22-negative tumors as SCLC?

As indicated above, the USP22-negative tumors are diagnosed as small cell lung carcinoma by thoracic pathologist Dr. Xu.

This should hopefully not require any additional mice, assuming fixed samples from previously grown xenografts are available. Either answer is useful to classify the type of resistance that USP22 inactivation confers. It would be interesting and surprising if there were such a large change in neuroendocrine gene expression but no effect on histology.

Reviewer #2 - SAGA complex (Remarks to the Author):

This manuscript by Best et al. investigates chemotherapy resistance in small cell lung cancer (SCLC) using patient-derived xenograft (PDX) models and in vivo CRISPR-Cas9 screening. The study identifies the deubiquitinase USP22, a core enzymatic component of the SAGA mDUB complex, as a determinant of therapy response in both ASCL1-positive and NeuroD-positive SCLC subtypes.

The in vivo CRISPR screen is carefully designed and statistically robust, and the follow-up experiments convincingly show that USP22 loss confers therapy resistance and alters tumor biology. The work is particularly significant because it challenges the oversimplified view in the literature that USP22 is universally tumor-promoting, instead revealing a context-dependent role in therapy response. Moreover, the data showing that USP22 deficiency impairs activation of DNA damage response (DDR) proteins without changing their expression levels is both mechanistically intriguing and therapeutically relevant. As such the findings are potentially highly interesting and patient relevant.

Overall, the manuscript is very strong and highly interesting, with clear potential for publication in Nature Communications. Its main limitation is that the mechanistic insight into how USP22 mediates these effects remains limited.

Major Strengths

1. Use of highly relevant patient-derived models. The study uses multiple SCLC PDX models representing the ASCL1-positive and NeuroD-positive subtypes, which enhances the translational relevance and ensures the findings reflect patient biology rather than cell-line-specific phenomena.

2. Robust in vivo CRISPR-Cas9 screening. The in vivo screening is carefully executed with strong statistical support, leading to the confident identification of USP22 as a key mediator of therapy resistance. Figures 1–3, in particular, are clear and compelling.

3. Clear link to DNA damage response activation. The authors convincingly demonstrate that USP22 loss impairs the activation of DDR proteins while leaving their total expression unchanged. This adds an important mechanistic layer and highlights how chromatin-associated factors can regulate therapy sensitivity independently of gene expression changes.

4. High-quality data challenging simplistic literature. The work provides a strong counterpoint to the predominantly low-quality literature that describes USP22 as universally oncogenic, often by stabilizing MYC or similar proteins. The present study demonstrates a more nuanced, context-dependent biology supported by rigorous in vivo data.

Major Concerns and Suggestions

1. Mechanistic insight into USP22 function is limited. The mechanistic basis of USP22's effects remains incompletely resolved. The CUT&Tag data for H2A monoubiquitination (Figure 6G) show roughly balanced increases and decreases in peak intensity, with no strong unidirectional pattern. Global H2A monoubiquitination levels are not presented (e.g., by Western blot), and without occupancy data showing where USP22 resides on chromatin, it is difficult to claim a direct transcriptional regulatory role. The current data could be consistent with indirect effects rather than a primary chromatin-driven mechanism.

We agree with the reviewer's assessment of the strengths as well as the major weakness including incompletely resolving the mechanistic basis of USP22's effects. We have attempted USP22 CUT+RUN occupancy analyses,

but without success, likely owing to the quality of the antibodies available. We cannot rule out indirect effects and mention this in the Discussion on page 19.

2. Underutilization of mass spectrometry data and likely indirect mechanism. The mass spectrometry data presented in Figure 4 are highly interesting but remain underexplored. Based on prior experience with USP22 and SAGA biology, it is plausible that USP22's major functional role in this context is not as a direct chromatin regulator but rather through binding and stabilizing specific protein substrates. Such substrates could include transcription factors, cofactors, or other regulatory proteins that in turn mediate the downstream changes in DDR activation, therapy resistance, and possibly even the metabolic effects observed. Many previous attempts to connect USP22's phenotypes directly to H2A/H2B deubiquitination have been inconclusive, suggesting that the primary functional effects may instead arise through non-histone substrates. Mining the mass spectrometry dataset for proteins whose abundance or stability depends on USP22 would likely identify candidate mediators of the phenotypes observed and could provide a coherent mechanistic model.

The reviewer raises an important point. To identify proteins whose stability depends on USP22, we have now mined both proteomics and RNA-seq datasets from two of our isogenic SCLC PDX models FHSC14 and NCI109B with or without *USP22* deletion (NEW Supplemental Fig 6a-c). Also in Supplemental Tables 13-14, we now list all genes in sgUSP22 models downregulated at the protein level but not the RNA level (52 genes in FHSC14 and 18 genes in NCI109B). We could not immediately link any of these hits with the observed phenotypes or pathway alterations our USP22-null models. However, with these new analyses, we observe that SAGA complex deubiquitinase (DUB) members ATXN7L3 and ATXN7L2 were depleted at the protein level but not the RNA level in NCI109B (NEW Supplemental Fig. 6b), suggesting a destabilization effect of USP22 loss on the DUB module. Furthermore, USP27X, a related deubiquitinase that competes with USP22 to form SAGA-independent complexes with ATXN7L3 and ENY2, was the only hit shared between the USP22-null PDX models as being suppressed at the protein level upon USP22 deletion (NEW Supplemental Fig. 6c).

3. Limited integration with other SAGA/mDUB components. The manuscript would be substantially strengthened by including analyses that compare USP22 loss to the depletion of other mDUB components such as ATXN7L3 or ENY2, or by testing whether inhibition of the SAGA acetyltransferase module yields similar or divergent phenotypes. Even ex vivo or in vitro experiments would help determine whether the observed therapy resistance is specific to USP22 or reflects a broader SAGA/mDUB functional axis.

While we believe that testing effects of additional DUB, HAT, or SAGA core module perturbations on SCLC chemoresistance would be highly interesting, generating these isogenic PDX models will take substantial (at least 6-12+ months) time due to the fact that these PDX are limited to growing only in mice during model generation. We note in the point above that USP22 deletion also leads to decreased protein abundance of ATXN7L3, at least in model NCI-109B. We also note that in addition to USP22, other SAGA components including TAF5L, TADA1, TADA6L, and TADA2B were strong hits in our genetic knockout screens (Fig. 1d-f), suggesting that loss of function of multiple SAGA components, apart from USP22, may additionally confer SCLC chemoresistance, although this remains to be validated.

4. Glycolysis data feel conceptually disconnected. The findings involving glycolytic inhibition in Figures 7 and 8 are interesting but not clearly integrated into the central mechanistic narrative. Connecting the metabolic findings more explicitly to the therapy-resistance phenotype would strengthen the story. For example, demonstrating that BAY876 treatment reproduces the effects observed in Figures 7F–G, or providing a clear rationale linking glycolytic modulation to impaired DDR activation, would help unify the manuscript.

Thank you for this suggestion. To strengthen the connection between increased glycolytic programs and reduced DDR signaling in USP22-null models and given our previous results showing BAY-876 treatment restoring chemosensitivity to USP22-null models (Fig. 8d), we have now performed IF on explanted cells from FHSC14 sgCtrl vs sgUSP22 PDX tumors treated in vitro with saline, cisplatin, BAY-876, or combination to test if Glut1inhibition would increase γH2AX in FHSC14 sgUSP22 tumors (NEW Fig. 8e, NEW Supplemental Fig. 9c). In accordance with our previous results, cisplatin treatment robustly induced γH2AX in sgCtrl cells but not in sgUSP22 cells. While BAY-876 treatment did not increase γH2AX in sgUSP22 cells alone as a single-agent,

combination treatment of BAY-876 with cisplatin significantly increased γH2AX in sgUSP22 cells compared to cisplatin-only treatment. These results demonstrate increased cisplatin-induced DNA damage signaling occurring in FHSC14 sgUSP22 models during the re-sensitization to chemotherapy by GLUT1 inhibition.

5. Patient data. It is somewhat surprising that, given the very interesting in vivo data and potential patient relevance, that the authors do not provide any patient-derived data related to USP22 in SCLC. It would be interesting to know (either through TCGA analyses or IHC staining of patient samples) whether USP22 may also be lost in a subset of SCLC patients.

In NEW Supplemental Table 6, we mined multiple genomic SCLC datasets from Rudin et al (2012), Gardner et al (2017), George et al (2015), and George et al (2024) to identify USP22 mutations in SCLC patients. (SCLC was not among the tumor types included in TCGA). This analysis uncovered 2 missense mutations and 4 different loss-of-function mutations (1 frame shift and 1 in-frame deletion from a single patient, 1 nonsense mutation from another patient, and 1 nonsense mutation in the PDX model JHU-LX33 used in our current study). As mentioned in the Discussion, the vast majority of the genomic information available for SCLC are from untreated patients, and USP22 is not included in most targeted panels that are more often applied in the clinic. Thus, studies that rely on such panels and include therapy relapsed patients do not report on USP22 status.

We also mined transcriptional data for USP22 expression from unpaired treatment-naïve vs chemorefractory patient tumor samples (George et al, 2024). This analysis revealed a decrease in USP22 expression in chemorefractory samples relative to treatment-naïve samples (see NEW Reviewer Fig. 1 below). We decided not to include these data in the current manuscript owing to the heterogeneity in tumor material, with some samples from PDX models and thus lacking key immune cells, and others from primary or metastatic tumors. We include the data for the reviewer below:

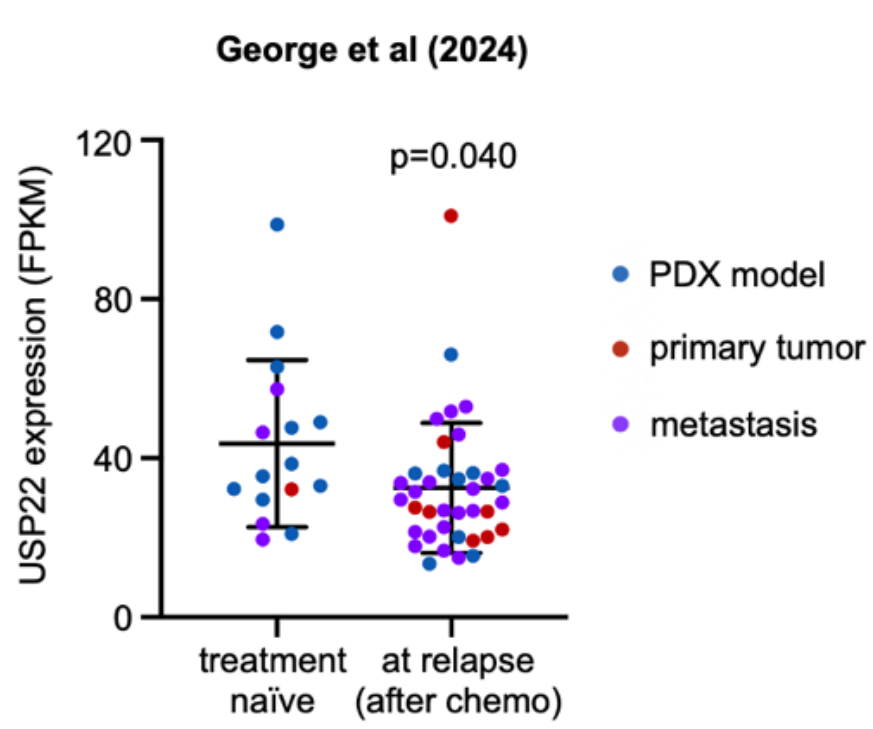

Reviewer Figure 1. Expression data for USP22 in treatment naïve vs at relapse (after chemo) patient tumors from George et al, Nature (2024) dataset.

Overall, this study provides very interesting and solid in vivo screening results in highly relevant model systems. The findings are generally well presented, clear and convincing. However, the actual mechanism(s) by which USP22 functions in SCLC remain largely unclear. The authors have generated extensive proteome and transcriptome datasets which have only been superficially analyzed, which may, in fact, potentially be exploited to find more detailed mechanistic insights into USP22 biology in SCLC.

We hope the changes that include deeper mining of proteomic and RNA-seq data help improve our story. USP22 loss has pleiotropic effects and future studies will build from this work to better define the mechanisms through which USP22 functions and regulates response to chemotherapy, and others will be able to mine our extensive genomic and proteomic data.

Reviewer #3 - in vivo CRISPR, cancer (Remarks to the Author):

Best et al. performed a targeted in vivo screen in PDX models of small cell lung cancer (SCLC) to understand the mechanisms of chemo-resistance. The authors identified USP22, a part of SAGA complex as a key molecule

whose deletion conferred resistance to PDX models. They further show that USP22 loss increased H2A-K119 monoubiquitylation and further attenuated DNA damage driven-phosphorylation events and apoptosis. In addition, they show glycolysis could be key targetable vulnerability in USP22 null tumors.

Overall, the manuscript is well written, and the data is presented clearly with a few draw backs. The use of 3 different PDX lines (USP22 positive and null) adds strong evidence for the role of the protein, both knock out and rescue experiments are well done. The first couple of figures clearly establish the role of USP22 in SCLC chemoresistance with multiple experiments and strong data.

The findings are novel and impactful, linking USP22 loss to chemoresistance through epigenetic and metabolic reprogramming. The large amount of omics data further serve as a valuable resource for the community.

However, despite the strong data, the paper may also need mechanistic depth and better integration of stories. In light of the standards of Nature Communications, a few experiments and clarifications are needed to strengthen the mechanistic insights and translational relevance. In brief, the authors can further transform it from a "USP22 is important" study to a "USP22 connects epigenetics, metabolism, and DDR in chemoresistance" breakthrough.

Major Concerns and Required Experiments

1. General Storyline – Lack of Cohesive Narrative Between Key Findings

Issue: The paper presents several independent observations (e.g., USP22 loss → H2AK119ub → neuronal gene suppression; glycolysis upregulation; DDR impairment), but these stories feel disconnected. The authors could mechanistically link these pathways to show how they collectively drive chemoresistance. This reviewer also acknowledges that sometimes connecting these different mechanisms could require more experiments and could be beyond the scope of this study. As such, a strong discussion paragraph should be added about the disconnect/shortcomings of the study. Alternatively if the authors could provide a cohesive connection and narrative, the manuscript will be strengthened.

Thank you for this comment. During these revisions, we have provided a link between glycolysis upregulation and DDR impairment in USP22-null models (please see response to Point 3 below). We recognize that we have not established precise mechanisms through which the pleiotropic phenotypes associated with USP22 loss are connected, however, we have fleshed out the narrative about USP22 regulation of glycolysis, which we show clearly impacts chemotherapy response.

2. Mechanistic Link Between USP22 Loss and Chemoresistance

Issue: While the study demonstrates that USP22 loss leads to chemoresistance, the direct mechanistic link between USP22's deubiquitylase activity and the observed phenotypic changes (e.g., H2AK119ub, glycolysis, DNA damage response) needs further specific validation. For example, rescue experiments with USP22 catalytic mutant USP22-C185S to show that catalytic activity is required for chemosensitivity is well done. However, additional mutants (e.g., USP22 lacking H2A/H2B deubiquitylation specificity) could be tested to dissect whether H2AK119ub or other substrates are critical. Alternatively, functional validation of H2AK119ub targets by using CRISPR/dCas9 to tether ubiquitin ligases (e.g., RING1B) or deubiquitylases to neuronal gene promoters in USP22-null cells to test if H2AK119ub deposition alone drives chemoresistance. There are other possible experiments to show and connect these by functional validation. The reviewer also acknowledges that not all experiments are required. Showing one experiment to connect the specificity could also address the shortcomings of cohesive narrative (point 1).

Thank you for this comment. Generating overexpression or genetic knockouts in PDX models is a process that takes a minimum of 6+ months due to the nature of these models needing to grow in vivo during the entire selection/purification process. However, we have provided further mechanistic link between the central findings of glycolysis regulation and DDR impairment in Point 3 showing that inhibition of GLUT1 leads to increased levels of cisplatin-induced DNA damage in chemoresistant USP22-null PDX models.

3. Glycolysis as a Therapeutic Vulnerability

Issue: The GLUT1 inhibitor BAY-876 resensitises USP22-null tumors, but the metabolic dependency is not fully characterized. Metabolomic profiling could be used to compare metabolite levels (e.g., lactate, ATP, TCA intermediates) in USP22-null vs. wild-type tumors ± cisplatin/etoposide to confirm glycolytic flux changes. Alternatively, genetic inhibition of glycolysis by Knockdown or knockout of HK2 or SLC2A1 (GLUT1) in USP22-null tumors to validate that glycolytic genes are essential for chemoresistance. Other alternative could be testing epigenetic-metabolic crosstalk: Treat USP22-null cells with H2AK119ub inhibitors (e.g., PRC1/2 inhibitors like GSK126 or UNC1999) and measure glycolytic gene expression (e.g., GLUT1, HK2) to test if H2AK119ub directly regulates metabolic reprogramming. Another possibility to test DDR-glycolysis connection: Treat USP22-null cells with 2-deoxyglucose (2-DG, glycolysis inhibitor) and measure γH2AX or RAD51 foci to test if glycolysis suppression restores DNA damage sensitivity. Alternatively, Test if LDHA knockdown (blocks lactate production) rescues cisplatin sensitivity in USP22-null cells.

These are multiple experiments and reviewer acknowledges that some of these could be beyond the scope of the current work. However, performing at least one of the proposed experiments that is feasible for authors in a quick span of time and show the connection could strengthen the manuscript and also connect the narrative cohesively.

Thank you for these suggestions. To further characterize the metabolic findings in our USP22-null models, we treated FHSC14 sgCtrl and sgUSP22 tumors saline or cis-eto in vivo for 48 hours and performed a glycolytic stress test Seahorse assay on explanted tumor cells (NEW Fig. 8c). We observed that Maximum Glycolytic Rate was significantly increased in cis-eto treated sgUSP22 cells compared to cis-eto treated sgCtrl cells.

We further strengthened the connection between our metabolic and DDR findings by performing IF for γH2AX on explanted FHSC14 sgCtrl vs sgUSP22 tumor cells treated in vitro for 48 hours with saline, cisplatin, BAY-876, and in combination (NEW Fig. 8e). While sgUSP22 cells exhibited decreased cisplatin-induced γH2AX relative to sgCtrl cells (in agreement with our previous in-vivo treatment data in Fig. 7e), we found that inhibiting GLUT1 significantly increased cisplatin-induced γH2AX signal in sgUSP22 cells. These results establish a link between the increased glycolytic programs and blunted DDR signaling observed in our SCLC PDX models with *USP22* deletion.

4. Clinical Relevance and Translation: The study could be strengthened by showing USP22 loss occurs in relapsed SCLC patients or correlates with poor outcomes. It could be done by analyzing clinical cohorts (if available): Perform IHC or targeted sequencing for USP22 mutations/loss in pre- and post-chemotherapy SCLC patient samples (e.g., from TCGA or institutional cohorts). Correlation with survival: If patient data is available, assess whether USP22 loss/mutation correlates with progression-free or overall survival. Single-cell RNA-seq: Analyse published datasets (e.g., from George et al., 2024) to determine if USP22-low cells are enriched in relapsed SCLC. From any of the above 3 proposed analysis, the feasible one could be done and it could strengthen the clinical relevance.

We did not find single-cell RNA seq data in George et al. 2024. However, from this study we did mine transcriptional data for USP22 expression in treatment-naïve vs treatment relapsed patient or patient-derived samples. Treatment relapsed samples exhibited a decrease in USP22 expression relative to treatment-naïve samples (see Response to Reviewer 1 and Reviewer Fig. 1 above). We decided against including this data in the current version of the manuscript, however, owing due to the heterogeneity in sample type (PDX vs tumor) and sites of collection.

Minor Concerns and Clarifications

1. Figure 1: May please clarify how sgRNA enrichment was normalised across pools. E.g. were batch effects considered?

We first randomly combined samples into pools due to sparse counts, and then gRNA counts were median normalized after pooling for each pool separately, which is the standard normalization method provided by

MAGeCK. Since normalization happens after randomized pooling, each pool effectively has the same distribution and any batch effects would be mitigated by the random pooling. Adjustment for batch effects pre-pooling would be difficult due to sparse counts.

2. Figure 2: Include quantification of tumour regression (e.g., % complete response) in addition to growth curves.

Quantifications of tumor responses (% Change in Tumor Volume from initial to final measurement) for each growth curve study are now provided in NEW Supplemental Fig. 2b,d; NEW Supplemental Fig. 3b,c; and NEW Supplemental Fig. 9b.

3. Figure 5: Provide RNA-seq validation (e.g., qPCR) for key neuronal genes (ASCL1, NKX2-2).

Using approaches orthogonal to RNA-seq, we demonstrate that key neuronal markers such as ASCL1 and INSM1 are decreased in sgUSP22 models at the protein level by western blot (Fig. 6h) and IHC (NEW Supplemental Fig. 6a-e). NKX2-2 levels are similarly shown to be decreased at the protein level (Fig. 4a, Fig. 6h). We suggest that these orthogonal approaches provide much stronger validation vs qPCR.

4. Figure 7: Show representative images for γH2AX foci in immunofluorescence assays.

White arrows pointing to γH2AX foci in representative images for immunofluorescence assays are now provided in NEW Supplemental Fig. 8g and Supplemental Fig. 9c.

5. Discussion: Address why USP22 loss has stronger effects in FHSC14 (ASCL1-high) vs. NCI109B (NEUROD1-high). Is this subtype-specific?

NCI109B is one of the most chemosensitive PDX models of SCLC we use, exhibiting a much higher degree of chemosensitivity compared to FHSC14 (ASCL1-high) or JHU-LX33 (NEUROD1-high). A greater number of PDX models from ASCL1-high and NEUROD1-high subtypes will need to be tested before determining whether the magnitude of resistance upon USP22 loss is subtype-specific. We were struck by the observation that all 3 models examined exhibited some degree of resistance with USP22 loss despite widespread differences in their biology. We now mention some of these points on page 20.

Conclusion

The manuscript identifies USP22 as a critical regulator of SCLC chemoresistance with compelling in vivo data. However, the mechanistic gaps and lack of clinical validation limit its impact. Addressing these issues would solidify the study's significance for *Nature Communications*. Specifically:

1. Prove USP22's direct role in H2AK119ub and gene silencing.

2. Validate glycolysis as a targetable vulnerability with orthogonal approaches.

3. Link USP22 loss to clinical chemoresistance in patient samples.

We have not proven USP22's direct role in H2AK119up and gene silencing, which is challenging using constitutive gene deletions, and now comment on this (page 19). We have shown that USP22 is indeed a critical regulator of SCLC chemoresistance and have provided new insights into USP22 control of DNA damage response, neuroendocrine gene expression programs and glycolysis.

Most of the whole genome or whole exome sequencing genomic data available for SCLC are from untreated patients, and while extensive targeted panel data are available from treated patients, these panels do not include USP22. We and other groups are now using ctDNA to examine the genomics of relapsed SCLC and we have incorporated USP22 into our own custom targeted panels that we are applying so SCLC patient samples. However, it will take some time to perform these analyses. We focus this study on the biology of USP22

perturbation in SCLC, while future work, beyond the scope of the present study, will address the genomics of relapsed SCLC, including whether or not genetic alteration of USP22 or other SAGA members contributes.

Finally, we have expanded our investigation of USP22 in regulation of glycolysis in SCLC.

Reviewer #4 (Remarks to the Author):

I co-reviewed this manuscript with one of the reviewers who provided the listed reports. This is part of the Nature Communications initiative to facilitate training in peer review and to provide appropriate recognition for Early Career Researchers who co-review manuscripts.

REVIEWER COMMENTS

Reviewer #1 (Remarks to the Author):
This was already an excellent manuscript on initial submission, and the authors have improved it substantially. They have now shown that USP22-negative tumors remain histologically consistent with SCLC despite reduced expression of neurodevelopmental transcription factors such as ASCL1, NEUROD1, and INSM1. This is a significant mechanistic insight that distinguishes acquired chemoresistance through loss of SAGA complex activity from other alterations, such as MYC amplification, that induce substantial histologic transformation. Of course, there are further questions to answer, such as the role of other SAGA complex components and the critical targets of USP22 for chemosensitivity, but without an in vitro model that fully recapitulates clinical chemoresistance, which does not exist for SCLC, these experiments will need to be performed in vivo in PDX models and will take years. This story is of general interest and is ready to be reported in its current form to the lung cancer research community.

Reviewer #2 (Remarks to the Author):
Overall, my enthusiasm for this manuscript remains. The study provides very interesting and solid data. However, the authors side stepped and still have not addressed one of my major concerns. In my first comment I specifically mentioned that it would be important to analyze global H2A monoubiquitination levels (i.e. by Western blot). Given the strong emphasis placed on this modification in the manuscript (and little consideration of altnernative mechanisms), I would consider this to be important (regardless of the outcome). Furthermore, it does not seem unreasonable to also test the extent to which other SAGA components may be involved. For example, given the authors' findings, one may expect that enzymatic inhibition of GCN5 (e.g., using CPTH6) may elicit similar effects as USP22. And even if not, this would be interesting.

> To address the request to analyze global H2A mono-ubiquitination, we performed western blot analysis for histone H2AK119, total H2A, and H3 (as an additional loading control) on acid-extracted histones from tumor samples of all 3 SCLC PDX models used in the current study with or without *USP22* deletion. Across the 3 PDX models we did not observe any robust change in H2AK119ub in USP22-null models relative to control models (New Supplemental Figure 7). We agree with the reviewer that this is an important addition to the manuscript.
>
> While it may be interesting to test if enzymatic inhibition of GCN5 would elicit similar effects as USP22, we note that GCN5 was not a hit in our genetic screens. Our study focuses on in vivo experiments and we lack cell lines that recapitulate the impact of SAGA complex loss on responses to chemotherapy in culture. We performed some studies of short-term ex vivo cultures of PDX models in our manuscript, but even to perform ex vivo experiments would take months, as we do not have the PDX models growing in mice. We also note that Reviewer 1 has weighed in on this issue and concurs with this assessment.
>
> After consultation with the editor, for this revision, we have prioritized the H2A western blot experiments that the reviewer requested.

Note: In comment 3, reviewer 3 mentioned inhibition of EZH2 (PRC2 component) as "H2K119ub inhibitors". Although PRC2 and PRC1 are clearly intertwined, I would certainly not characterize EZH2 inhibition as "H2K119ub inhibition".

> We agree that EZH2 inhibition is not equivalent to H2BK119ub inhibition.

Reviewer #3 (Remarks to the Author):

Dear Editor,

I thank the authors for their thoughtful and comprehensive response to the comments and suggestions. I appreciate the significant efforts they have made to address the points raised, and I am pleased with the additional data and clarifications they have provided.

I am satisfied with the revisions, particularly the strengthened connection between glycolysis upregulation and impaired DNA damage response in USP22-null models, along with the added histological and immunohistochemical data, and expanded analysis of proteomic and transcriptomic datasets that further solidify the clinical relevance of the findings. I understand the practical considerations and time constraints involved in generating additional genetic models or performing certain mechanistic experiments in PDX systems, and I agree that the new experiments they have included meaningfully enhance the narrative and mechanistic insight of the study. I appreciate the expanded discussion regarding the potential subtype-specific effects and the limitations of linking H2AK119ub directly to gene silencing in this context.

I thank author's effort to improve the previously fragmented core mechanistic narrative linking USP22 loss to chemoresistance. Although connections between glycolysis and DDR impairment have been strengthened, my very minor concern remains as the seemingly absent unified model explaining how USP22's deubiquitinase activity coordinately regulates neuroendocrine identity, metabolic reprogramming, and DNA damage response. While I completely understand the practical constraints, to be critical, the absence of rescue experiments using substrate-specific USP22 mutants or direct modulation of H2AK119ub at target gene loci leaves open the question of whether the observed phenotypes are direct consequences of USP22 catalytic activity or indirect effects of SAGA complex destabilization.

We thank the reviewer for their positive comments on our initial revision. While experiments in Figure 3E, using a catalytically dead version of USP22 for add-back experiments provide some support for the notion that the enzymatic activity of USP22 is important for response to chemotherapy, we agree that it is a limitation of our study that we have not provided a unified model and we agree that indirect effects of USP22 loss could contribute to changes in H2AK119ub levels and other phenotypes observed. We have noted these limitations in the discussion.

Page 19 **"However, we note that with constitutive *USP22* deletion it can be challenging to discern direct from indirect effects of USP22 loss. Future studies to link USP22 genomic occupancy to sites of altered H2A-K119ub and testing the impact of modulating H2A-K119ub on observed phenotypes would help to overcome this limitation to the study."**

That being said, I believe that the paper meets the rigor of Nature Communications, provided the limitations are transparently acknowledged in the discussion, as the authors have begun to do.

Overall, I believe the manuscript has been substantially improved and now presents a more cohesive and compelling story.

Reviewer #4 (Remarks to the Author):
I co-reviewed this manuscript with one of the reviewers who provided the listed reports. This is part of the Nature Communications initiative to facilitate training in peer review and to provide appropriate recognition for Early Career Researchers who co-review manuscripts.
